# ALIGNED Network for rare cerebrovascular diseases: methodology and preliminary results

**DOI:** 10.1007/s10072-026-09183-1

**Published:** 2026-06-22

**Authors:** Carolina De Toma, Antonella Potenza, Nicola Rifino, Camilla Strazzabosco, Benedetta Storti, Giulia Marinoni, Tatiana Carrozzini, Gemma Gorla, Giuliana Pollaci, Elisabetta Pasella, Alice Mallia, Isabella Canavero, Giorgio Battista Boncoraglio, Irene Scala, Esteban Zacarias, Giulia Fusi, Francesca De Giorgi, Matteo Foschi, Simona Sacco, Massimo Caulo, Cristina Banfi, Laura Gatti, Anna Bersano

**Affiliations:** 1 Cerebrovascular Unit, Fondazione IRCCS Istituto Neurologico Carlo Besta, Milan, Italy; 2 Department of Pharmacological and Biomolecular Sciences, University of Milan, Milan, Italy; 3 Unit of Functional Proteomics, Metabolomics and Network Analysis, Centro Cardiologico Monzino IRCCS, Milan, Italy; 4 Department of Neuroscience, Università Cattolica del Sacro Cuore, Largo Francesco Vito 1, Rome, Italy; 5 Unit of Informative Services, Fondazione IRCCS Istituto Neurologico Carlo Besta, Milan, Italy; 6 Department of Applied Clinical Sciences and Biotechnology, University of L’Aquila, L’Aquila, Italy; 7 Department of Neurosciences, Neurology Unit, S.Maria Delle Croci Hospital, AUSL Romagna, Ravenna, Italy; 8 Department of Neuroscience, Imaging and Clinical Sciences, G. d’Annunzio University, Chieti, Italy

**Keywords:** CADASIL, COL4A1 syndrome, Fabry disease, Sneddon’s syndrome, Moyamoya angiopathy, Rare cerebrovascular diseases

## Abstract

**Introduction:**

Cerebrovascular diseases are a major cause of morbidity/mortality worldwide, yet more than 30% of strokes remain of undetermined origin. Rare cerebrovascular diseases (rCVDs) contribute to this burden but are often underdiagnosed due to limited awareness, clinical heterogeneity, and fragmented diagnostic access. The ALIGNED project was established to address these gaps through a nationwide multidisciplinary network.

**Methods:**

ALIGNED was aimed to improve clinical/molecular characterization of rCVDs through standardized data collection, assess level of care of rCVDs by an online/on-site survey and reduce the geographical gap across Italy, by a virtual model of rCVDs care. In the first phase we conducted a survey to evaluate the availability of diagnostic/therapeutic pathways. Preliminary molecular profiling (i.e., transcriptomic/proteomic analyses) has been carried out on middle-cerebral artery and plasma samples from Moyamoya angiopathy (MMA) patients.

**Results:**

Fourty-nine centers adhered to the project. Initially, we collected a cohort of 308 subjects with rCVDs: CADASIL (162), COL4A1/A2-related disease (9), Sneddon syndrome (25), Fabry disease (32), and MMA (80). Web-based survey and site visits provided a representative overview of national capabilities in rCVD diagnosis and care. Transcriptomic analysis showed a peculiar expression profile of angiogenesis growth factors/inhibitors in MMA cerebral vessels. Plasma proteomic profiles of MMA patients highlighted circulating proteins expressed in dysfunctional angiogenesis.

**Discussion:**

Preliminary data suggested a substantial variability in the quality of rCVDs management and in the availability of diagnostic tools across Italy. Thus, mapping Italian expertise and facilities emerged as a crucial target for pinpointing gaps, improving resource allocation, standardizing rCVD care to guarantee equitable access for patients.

**Supplementary Information:**

The online version contains supplementary material available at 10.1007/s10072-026-09183-1.

## Introduction

Cerebrovascular diseases (CVDs) are one of the leading causes of morbidity and mortality worldwide [1]. Despite intensive investigations, more than 30% of strokes remain of undetermined origin. Rare CVDs (rCVDs), including heritable and acquired conditions, account for a proportion of these strokes. Heritable forms include: CADASIL (Cerebral Autosomal Dominant Arteriopathy with Subcortical Infarcts and Leukoencephalopathy), the most common monogenic small-vessel disease, characterized by recurrent ischemic strokes, cognitive decline, and migraines [2]; COL4A1/A2 syndrome, a disorder affecting the vascular basement membrane, leading to brain hemorrhages, ischemic strokes, and leukoencephalopathy [3] and Fabry disease, a X-Linked lysosomal storage disorder that causes progressive vascular damage, increasing the risk of stroke, particularly in young patients [4]. Acquired rCVDs include Sneddon’s syndrome, a non-inflammatory thrombotic vasculopathy manifesting with ischemic strokes and livedo reticularis and Moyamoya angiopathy (MMA), a progressive occlusive disease of the cerebral arteries that leads to chronic ischemia and hemorrhagic complications [5, 6].

Due to their rarity and clinical complexity, rCVDs are often misdiagnosed, as they require specialized knowledge and specific diagnostic tools. As a result, the diagnosis and management of these conditions is largely referred to experts making it difficult for the patients to have an equal access to the optimal diagnostic and therapeutic pathways. In Italy, most specialized centers are localized in the Northern regions, forcing many patients from Central and Southern areas to travel long distances to receive appropriate evaluation and treatment [7]. Indeed, according to the annual report by the Ministry of Health on Healthcare Mobility in Italy, the regions of Southern Italy are characterized by more pronounced migration flows when compared to those of the Northern and Central regions [8].

Moreover, the paucity of reported cases and limited literature on the phenotype, clinical course, and molecular features of rCVDs has hindered the development of effective therapies [9, 10]. Thereby, these conditions have remained ‘orphan’, as their rarity has led to insufficient support from both the scientific community and the healthcare system. However, early and accurate identification of these conditions is crucial, not only to define the most appropriate management strategies, including genetic counseling and available targeted therapies, but also to foster research progress.

Thus, the creation of a clinical and research network and the improvement of diagnostic pathways for rCVDs are critical to fill the current unmet needs. In fact, the limited number of expert centers across the country still represents a barrier to timely diagnosis and equitable access to care. To address this challenge, the ALIGNED project (RAre, but not aLone: a large Italian network to empower the impervious diaGNostic pathway of rare cerEbrovascular Diseases) was launched within the program *Next Generation EU—NRRP M6C2—Investment 2.1 Enhancement and strengthening of biomedical research in the NHS, Rare diseases (Call 2022)*.

By integrating a Virtual Hospital system [11] and optimizing resource allocation, ALIGNED aims to enhance diagnostic precision, improve understanding of rCVDs, and ensure equitable access to specialized care across the country, thereby reducing the need for costly and burdensome patient travel for consultations. As the first Italian multicenter cohort study on rCVDs, this project represents a crucial step toward reducing disease burden and advancing both clinical and molecular knowledge in the field. Thus, the purpose of the present work is to describe the methodological framework and preliminary results of the ALIGNED project.

### Study objectives and expected endpoints

The ALIGNED project aims to create a nationwide Italian network of clinical centers to collect a large cohort of rCVD patients, to be clinically and molecularly characterized and stratified for a better understanding of rCVD phenotype and clinical course. Additionally, this project has also the objective to homogenize and standardize diagnostic and therapeutic methodologies and pathways in Italy, thereby optimizing as much as possible the use of diagnostic resources across participating centers. Ultimately, ALIGNED is designed to develop a dedicated, multi-specialist, multicenter virtual model of care, with the dual purpose of reducing the burden and costs of patients travel for consultation and translating knowledge into improved diagnosis and management of rCVDs overall Italy.

## Methods

### Study design

**Fig. 1:**
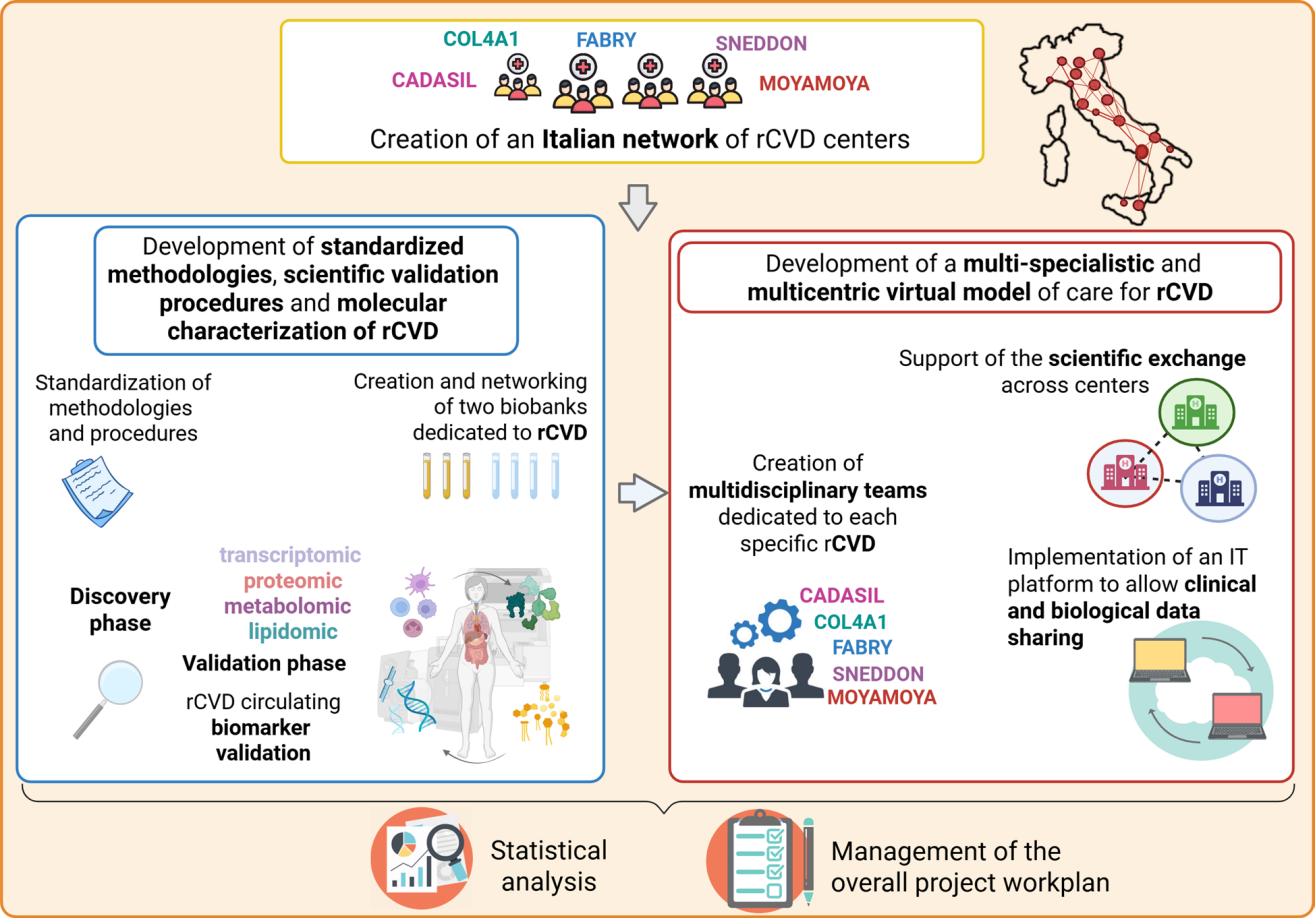
Representative framework of the ALIGNED project study design

This study is a multicenter, observational, retrospective and prospective national cohort study with a planned duration of 36 months (from May 2023 to May 2026). The study develops in two phases. The first phase of the project focused on the creation of a shared database to collect clinical and neuroimaging data of patients with rCVDs among participating centers using a virtual platform that guarantees anonymity and data protection (REDcap- https://project-redcap.org) [12]. The database is coordinated by the Cerebrovascular Unit, Fondazione IRCCS Istituto Neurologico “C. Besta”, Milan (FINCB), in collaboration with the FINCB Information Technology Unit (SIA). Through this infrastructure, clinical and molecular data on patients with rCVDs (CADASIL, COL4A1/2 syndrome, Fabry disease, Sneddon's syndrome and MMA) are being collected and shared between two referral hubs, the first in the north-center (FINCB), and the second in the south of Italy (Università dell’Aquila, UnivAQ). In parallel, an online REDCap survey was distributed to all participating clinical centers to assess the presence of diagnostic tools/techniques and the presence of structured diagnostic/therapeutic pathways. The survey is being validated through cross-sectional on-site visits and will provide a comprehensive overview of current practice in Italian hospitals managing rCVDs. This will inform optimization of existent resources and the implementation of shared diagnostic and therapeutic protocols. In the second phase of the study, a specific platform for second opinion consultations has been created by using the FINCB pre-existing telemedicine platform (TiCuro-http://telemedicina-besta.ticuro.io/besta/), specifically improved and adapted for ALIGNED by an external IT company in collaboration with SIA [13]. This platform enables multidisciplinary consultations on complex rCVD cases. A dedicated panel of specialists, including neurologists, neurosurgeons, neuro-radiologists, interventional radiologists, neuropsychiatrists, geneticists, nephrologists, cardiologists and/or neurophysiologists has been assembled. The expert team will hold virtual meetings to discuss challenging cases and provide patients with personalized diagnostic and management recommendations. A schematic representation of the ALIGNED study design is reported in Fig. 1.

### Study population

The present study includes patients of either sex, aged 18 years or older, with a clinical, genetic, and/or neuroradiological diagnosis of specific rCVDs (CADASIL, COL4A1/2 syndrome, Fabry disease, Sneddon's syndrome or MMA).

Inclusion criteria are:

1. The diagnosis of a rCVD, according to the following criteria:
2. CADASIL: presence of a *NOTCH3* gene variant [14];
3. COL4A1/A2 syndrome: a *COL4A1/A2* gene mutation [15];
4. Fabry disease: reduced α-galactosidase A (GLA) enzyme activity in males, or a *GLA* gene variant in females [16];
5. Sneddon's syndrome: presence of livedo reticularis confirmed by skin biopsy associated with a history of ischemic strokes [5];
6. MMA: conventional angiography (DSA) or MR angiography demonstrating unilateral or bilateral stenosis/occlusion of the terminal internal carotid arteries with compensatory collateral vessels, according with literature criteria published in 2021[17].
7. At least one brain MRI study with a minimal set of neuroradiological data (T1 and T2 weight and/or FLAIR, T2* weighted sequences and/or Gradient Echo (GE) and/or Susceptibility Weighted images (SWI), Diffusion Weighted Images (DWI)) is requested to be enrolled.

Patients with other potential causes explaining the cerebrovascular pathology are excluded. Additionally, patients unable to provide informed consent due to phasic or cognitive impairments, or those with contraindications to MRI (e.g., pacemaker, incompatible mechanical valves, claustrophobia), are excluded. Of note, the recruitment period refers to 35 months (May 2023 – April 2026).

### Study coordination and participating centers

Cerebrovascular Unit (UO1) of Fondazione IRCCS Istituto Neurologico Carlo Besta, Milan, Italy, (FINCB) coordinates the present study. Project partners are Centro Cardiologico Monzino IRCCS (Milan, UO2), Azienda Sanitaria Locale ASL2 Abruzzo Lanciano-Vasto-Chieti (UO3) and Università dell’Aquila (L’Aquila, U04).

UO1 and UO4 are referral centers for clinical data and biological samples collection originating from the North and the South area hospitals, respectively. All the Italian neurological and/or cerebrovascular units dedicated to the rCVDs diagnosis and care have been invited to participate to the study.

### Data collection

#### Clinical data

All patients underwent a standardized clinical assessment including a detailed collection of medical history (i.e., family history of neurological pathology, and of rCVD, cardiovascular risk factors, medications taken, comorbidities, recent or previous head injuries) and a complete neurological examination. For each patient, demographic and clinical data, including symptoms leading to neurological evaluation (i.e., type of neurological deficit, date of onset), and brain MRI reports were collected in the REDCap database, specifically dedicated to each rCVD.

#### Online and on-site survey

A REDCap-based online survey was distributed to participating centers to establish the availability of diagnostic instruments, genetic screenings, tools, facilities, dedicated outpatient services, diagnostic/therapeutic pathways, and human resources.

Randomly selected centers were then visited on-site to validate survey results (i.e., three hospitals from Northern Italy and two from Southern Italy). The eCFR for each disease and the survey is provided in the Supplementary Material (Supplementary files 2, 3, 4, 5, 6).

#### Biological data collection and analysis

After specific informed consent, biological samples (e.g., DNA, plasma, cerebrospinal fluid/CSF, cells and tissue specimens) of rCVD patients had been acquired for research purposes in all centers throughout Italy. According to local and national ethical rules, biological samples had been deposited in two biorepositories, located in the north (UO1) and south (UO4) of Italy, respectively. The samples had been scored anonymously according to data protection rules and all regulatory requirements for the handling and use of biological specimens, sample ownership and custody.

### Transcriptomic analysis

Identification of the molecular profiles of rCVDs was developed by transcriptomic approaches. Transcriptomic analyses were performed on middle-cerebral artery (MCA) fragments collected from patients with a confirmed diagnosis of MMA, undergoing neurosurgical bypass between superficial temporal artery (STA) and MCA at UO1. The collected biological samples were stored at a temperature of −80 °C and subsequently processed for total RNA extraction and for reverse transcription to cDNA. Using the Human Angiogenic Growth Factors & Angiogenesis Inhibitors RT2 Profiler PCR Array (QIAGEN, Hilden, Germany), a transcriptomic analysis (*discovery phase*) was conducted to compare the modulation of gene expression in cerebral vessels between Moyamoya patients and a cohort of subjects (CTRL) affected by unrelated CVDs that required an analogous neurosurgical bypass procedure (e.g., unruptured intracranial aneurysms). Statistical significance of the data was calculated by the non-parametric Mann–Whitney U test, using GraphPad Prism 8 software (GraphPad, Inc., San Diego, CA). The relative mRNA expression was calculated by the 2^−ΔΔCt^ comparative method using ACTB, β2M, GAPDH, HPRT1, and RPLP0 as the housekeeping genes. RNA samples extracted from MCA specimens of CTRL were chosen as the calibrators.

### Proteomic analysis

Plasma samples were collected from rCVD patients recruited by all the centers involved in ALIGNED. In order to carry out an exploratory proteomic investigation, the “Proximity Extension Assay” (PEA, Olink® Proteomics, Uppsala, Sweden) technology was used (*discovery phase*). This approach is based on the use of paired pairs of antibodies labeled with tags complementary to DNA oligonucleotide sequences [18]. Following binding to the target analyte, the oligonucleotide sequences, in proximity to each other, hybridize and are extended through a standard polymerase chain reaction (PCR). The portion of “DNA barcode” created is therefore unique and specific for each pair of antibodies. This portion is then amplified through quantitative PCR reactions. A total of 184 proteins were analyzed using two Olink® panels (Target 96 “Neurology” and Target 96 “Neuroexploratory” panels, respectively). Plasma samples of healthy donors (HD) have been used as controls. Statistical significance of the data was calculated by multiple Mann–Whitney U test using GraphPad Prism 8 software (GraphPad, Inc., San Diego, CA).

### Ethical issues

The study protocol was approved by the Ethical Committee of UO1 (FINCB, report n. 72; May 6, 2020), and by each participating center. The study was also performed in accordance with the current version of the Declaration of Helsinki. All procedures followed local clinical practice and disease guidelines. Written informed consent for study participation, including data and sample collection, was mandatory.

### Statistical consideration and analysis plan

It has been estimated that a total number of 500 patients may allow a realist overview of the natural history of rCVDs. Data analysis will be carried out centrally from the coordinating centers, using the full dataset. As the outcome is descriptive, an estimation of the statistical sample was not performed.

Descriptive statistics will be reported in terms of absolute numbers and percentages for categorical data, and using means with standard deviations (SDs), and medians with value ranges for continuous data. Several indicators will be established to assess the reliability of applied procedures and guidelines as well as to assess the efficacy of the network: (1) the number of patients obtaining a definite diagnosis, (2) the reduction of (2a) hospital admissions and (2b) time to diagnosis, (3) the number of cases needing a diagnostic revision and/or implementation of further investigation and/or intervention (pharmacological and/or neurosurgical). Statistical significance for binary categorical variables presented in the main tables of the manuscript was evaluated using the Chi-square test. To assess consistency between data collected via the online survey and during on-site visits, inter-rater agreement was calculated using Cohen’s kappa (κ).

## Results

### Study adherence and online survey participation

**Fig. 2:**
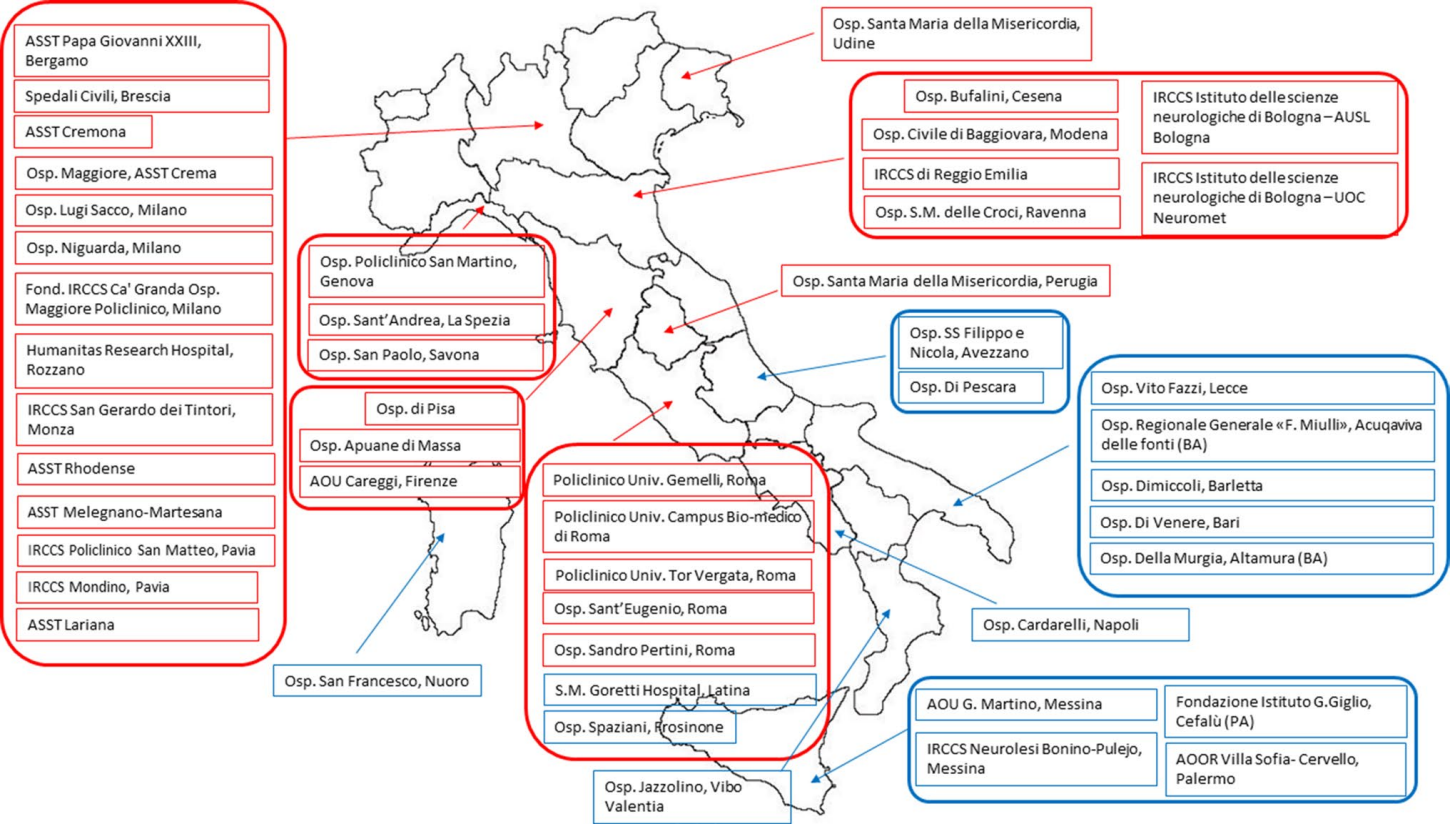
Representative map of Italy referring to ALIGNED participating centers, grouped based on region of provenance. Red color indicates centers from north of Italy, while blue color refers to centers from south of Italy.

Initially, 52 centers joined the project. Of these three centers were subsequently excluded due to non-adherence to scheduled deadlines. Therefore, 49 centers are actively participating and recruiting patients. In detail, the network includes 35 hospitals from Northern Italy and 14 from Southern Italy, as illustrated in Fig. 2.

In the first phase of the study, we collected a comprehensive cohort of 308 subjects with rCVDs, including patients affected by CADASIL (162), COL4A1/A2-related disease (9), Sneddon syndrome (25), Fabry disease (32), and MMA (80).

Fourty-four out of 49 (89.7%) participating centers (31 from Northern Italy and 13 from Southern Italy), with established expertise in rCVD fulfilled the proposed survey. Despite differences in the number of participating centers, nearly 100% of Northern centers and 92.3% of Southern centers are able to provide clinical-biochemistry analysis, including autoimmunity (e.g., ENA, ANA parameters) and thrombophilia (e.g., LAC, anti-cardiolipin antibodies) values. Regarding other immunological markers (i.e., anti-AchR, anti-MUSK and anti-cerebellar antibodies; autoimmune encephalitis panel) and neoplastic markers, heterogeneous availability was observed across all centers, independently from geographical area. RNF213 genetic screening was poorly available overall Italy: 25.8% of Northern centers and nearly 23.1% of Southern centers. In contrast, NOTCH3 genetic screening was accessible in 61.3% of Northern centers and 76.9% of Southern centers. Similarly, COL4A1/A2 genetic screening was available in 48.4% of Northern centers and 69.2% of Southern centers. Finally, GLA genetic screening was reported in 54.8% of Northern centers and 30.8% of Southern centers, while HTRA1 testing was available only in 41.9% of Northern centers and 23.1%% of Southern centers. The most relevant survey findings regarding biochemistry and genetic screening are reported in Supplementary file 7.

Analysis of the main available consultancies showed that first level specialist assessments (including neurological, cardiological, dermatological, and hematological evaluations) are provided in nearly all participating centers (Northern *vs* Southern regions: neurological 90.3% *vs* 84.6%; dermatological 96.8% *vs* 92.3%; hematological 100% *vs* 85.6%) as shown in Table 2. Notably, 25 out of 31 (80.6%) in the Northern regions reported providing genetic consultations, compared with only 8 out of 13 centers (61.5%) in the Southern centers. Remarkably, access to a nutritionist consultancy is possible in 96.8% of centers in the Central-Northern regions, compared to 53.8% of centers in the Southern region (p = 0.000382). Similarly, hepatology consultations are accessible in 96.8% of centers in the Central-Northern regions *vs* 76.9% of centers in the Southern region (p = 0.036636). Additionally, the availability of neuropsychological evaluations has been reported in 28 out 31 centres (90,3%) in the Northern regions, compared with 10 out 13 (76.9%) in the Southern centers. The most relevant survey findings regarding the main available consultancies are reported in Supplementary file 8.

Lastly, second-tier assessments such as advanced MR techniques (64.5% *vs* 30.8%), brain CT perfusion (87.1% *vs* 69.2%), cerebral diagnostic angiography (80.6% *vs* 69.2%), brain SPECT imaging with acetazolamide challenge (19.4% *vs* 7.7%), and transcranial doppler studies (96.8% *vs* 85.6%), are predominantly available in centers from the Northern regions when compared to Southern ones, as reported in Table 1. Of note, the availability of specialized assessments such as MR perfusion (p = 0.002594), vasomotor reserve study via transcranial doppler (p = 0.025748), autonomic nervous system evaluation (p = 0.003707), polysomnogram execution (p = 0.010377), skin biopsy (superficial and deep, p = 0.001653) and muscle biopsy (p = 0.025748) was greater in centers from Central-Northern regions as compared to those of Southern ones.

**Table 1:** List of the main instrumental examination available at the 31 centers from the Northern regions of Italy and in the thirteen centers located in the Southern ones

	CENTERS FROM CENTRAL-NORTHERN REGIONS OF ITALY. N = 31		CENTERS FROM SOUTHERN REGIONS OF ITALY. N = 13		
Instrumental examinations available in the centre	Yes (n/N; %)	No (n/N; %)	Yes (n/N; %)	No (n/N; %)	p-value
Comprehensive echocardiographic assessment	31; (100)	0; (0)	13; (100)	0; (0)	-
Heart MR	26; (83.9)	5; (16.1)	9; (69.2)	4; (30.8)	0.272019
Comprehensive CT and angio-CT assessment	31; (100)	0; (0)	13; (100)	0; (0)	-
Brain MR and cerebral angio-MRI	31; (100)	0; (0)	13; (100)	0; (0)	-
MR perfusion	26; (83.9)	5; (16.1)	5; (38.5)	8; (61.5)	**0.002594**
Advanced MR techniques*^.a^	20; (64.5)	11; (35.5)	4; (30.8)	8; (61.5)	0.064765
Brain CT perfusion ^b^	27; (87.1)	4; (12.9)	9; (69.2)	3; (23.1)	0.335159
Cerebral diagnostic angiography	25; (80.6)	6; (19.4)	9; (69.2)	4; (30.8)	0.409762
Vasomotor reserve study via transcranial Doppler	16; (51.6)	15; (48.4)	2; (15.4)	11; (85.6)	**0.025748**
Transcranial doppler study	30; (96.8)	1; (3.2)	11; (85.6)	2; (15.4)	0.144323
Epiaortic vessel echocolordoppler	31; (100)	0; (0)	13; (100)	0; (0)	-
Autonomic nervous system evaluation	17; (54.8)	14; (45.2)	1; (7.7)	12; (92.3)	**0.003707**
Comprehensive neurophysiological assessment	30; (96.8)	1; (3.2)	12; (92.3)	1; (7.7)	0.516375
Polysomnogram execution	26; (83.9)	5; (16.1	6; (46.2)	7; (53.8)	**0.010377**
EEG	31; (100)	0; (0)	13; (100)	0; (0)	-
Gastro and colonscopy	30; (96.8)	1; (3.2)	12; (92.3)	1; (7.7)	0.516375
Skin biopsy (superficial and deep)	23; (74.2)	8; (25.8)	3; (23.1)	10; (76.9)	**0.001653**
Muscle biopsy	16; (51.6)	15; (48.4)	2; (15.4)	11; (84.6)	**0.025748**
SPECT acetazolamide	6; (19.4)	25; (80.6)	1; (7.7)	12; (92.3)	0.334551
PET total body	24; (77.4)	7; (22.6)	9; (69.2)	4; (30.8)	0.567109
Cerebral PET with amyloid tracer	16; (51.6)	15; (48.4)	6; (46.2)	7; (53.8)	0.741078

One value is missing in the survey, therefore its status (available or unavailable) is unknown for centres from Southern regions (^a.b^). Chi Square test has been used to assess statistical significance, when applicable, P value < 0.05 are considered statistically relevant. Abbreviations: Computed Tomography, CT; Electroencephalogram, EEG; Magnetic Resonance, MR; Single Photon Emission Computed Tomography, SPECT; Positron Emission Tomography, PET. The asterisk (*) in the table refers to two advanced MRI techniques: ASL (Arterial Spin Labeling) and BOLD (Blood Oxygen Level Dependent).

**Fig. 3:**
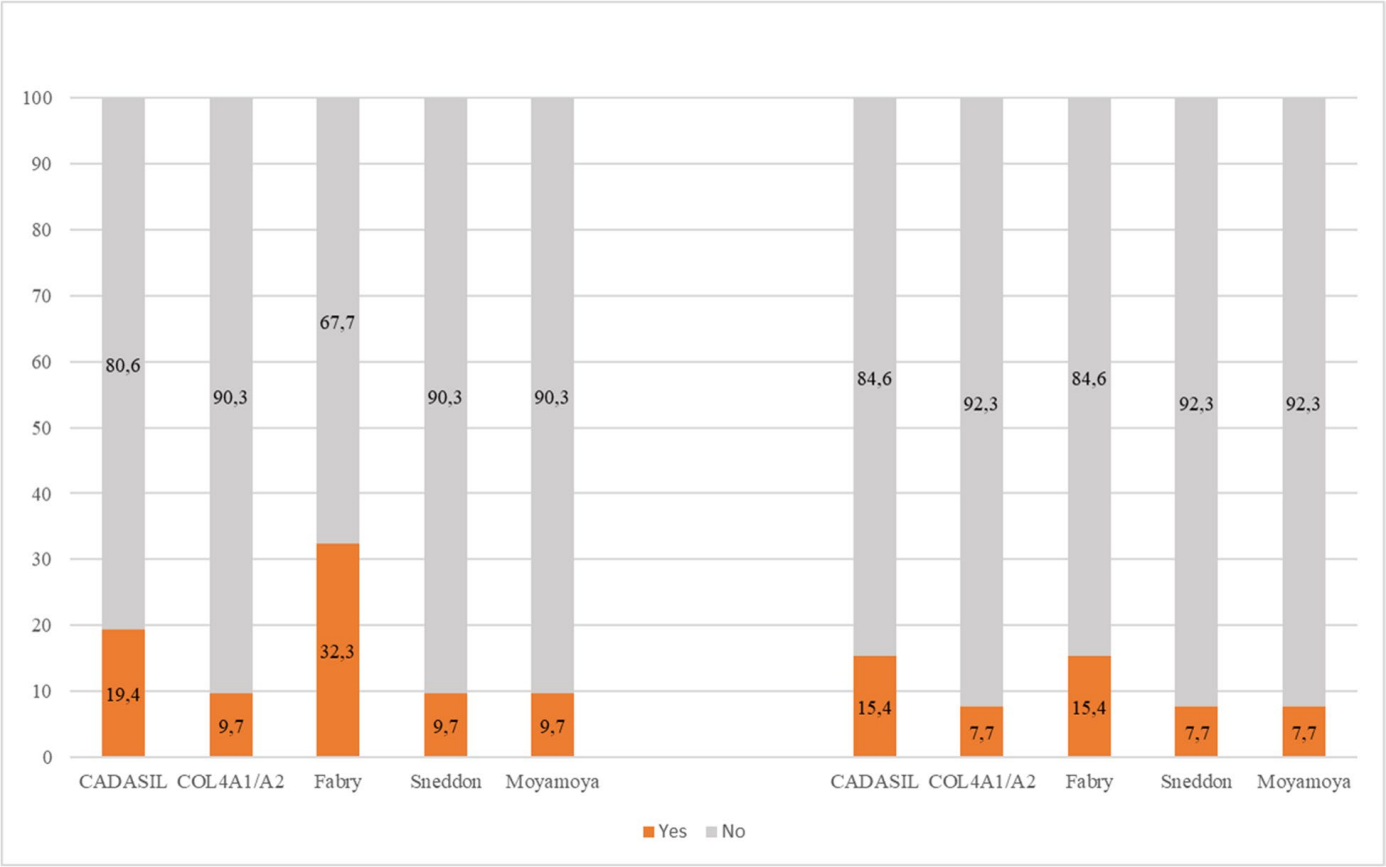
Figure 3: Representative panel illustrating the availability of diagnostic and treatment protocols currently adopted by centers fulfilling the survey. Data are reported in terms of percentage. Orange bars represent those centers that referred to the accessibility of established programs whereas grey bars indicate those centers that declared the absence of specific diagnostic and treatment protocols.

In addition, analysis of the availability of standardized diagnostic and therapeutic protocols among centers completing the survey showed that Northern regions tend to provide more specific care programs, compared with Southern centers, as illustrated in Fig. 3. In particular, a higher proportion of Northern centers follow dedicated protocols for Fabry disease care (32.3% *vs* 15.4% in Northern and Southern regions, respectively). Similarly, a slightly higher percentage of Northern centers declared having specific protocols for CADASIL (19.4% *vs* 15.4%), COL4A1/A2 (9.7% *vs* 7.7%) and Sneddon’s syndrome (9.7% *vs* 7.7%), when compared to those from the south. No statistically significant differences were observed in the availability of diagnostic and treatment protocols between centers located in the Northern and Southern regions, as shown in Supplementary file 9.

Finally, three centers from the northern-central and two centers from the southern part of Italy were randomly selected in order to validate the presented results: on-site visits demonstrated that the centers visited complied with the information provided in the survey and the existing equipment in their centers. Specifically, when comparing data obtained through the online survey and the on-site visits, inter-rater agreement was excellent in 4 out of 5 centers (Cohen’s κ = 0.93, 0.96, 0.95, and 0.90) and good in the remaining center (Cohen’s κ = 0.66).

### Molecular profiles of rCVD patients

In order to highlight novel and unpredictable mechanistic, diagnostic and prognostic markers, an explorative analysis has been accomplished in plasma and tissue specimens of rCVD patients, to identify key molecular signatures and dissect relevant mechanisms across omics scales.

#### Tissue transcriptomic analysis

**Fig. 4:**
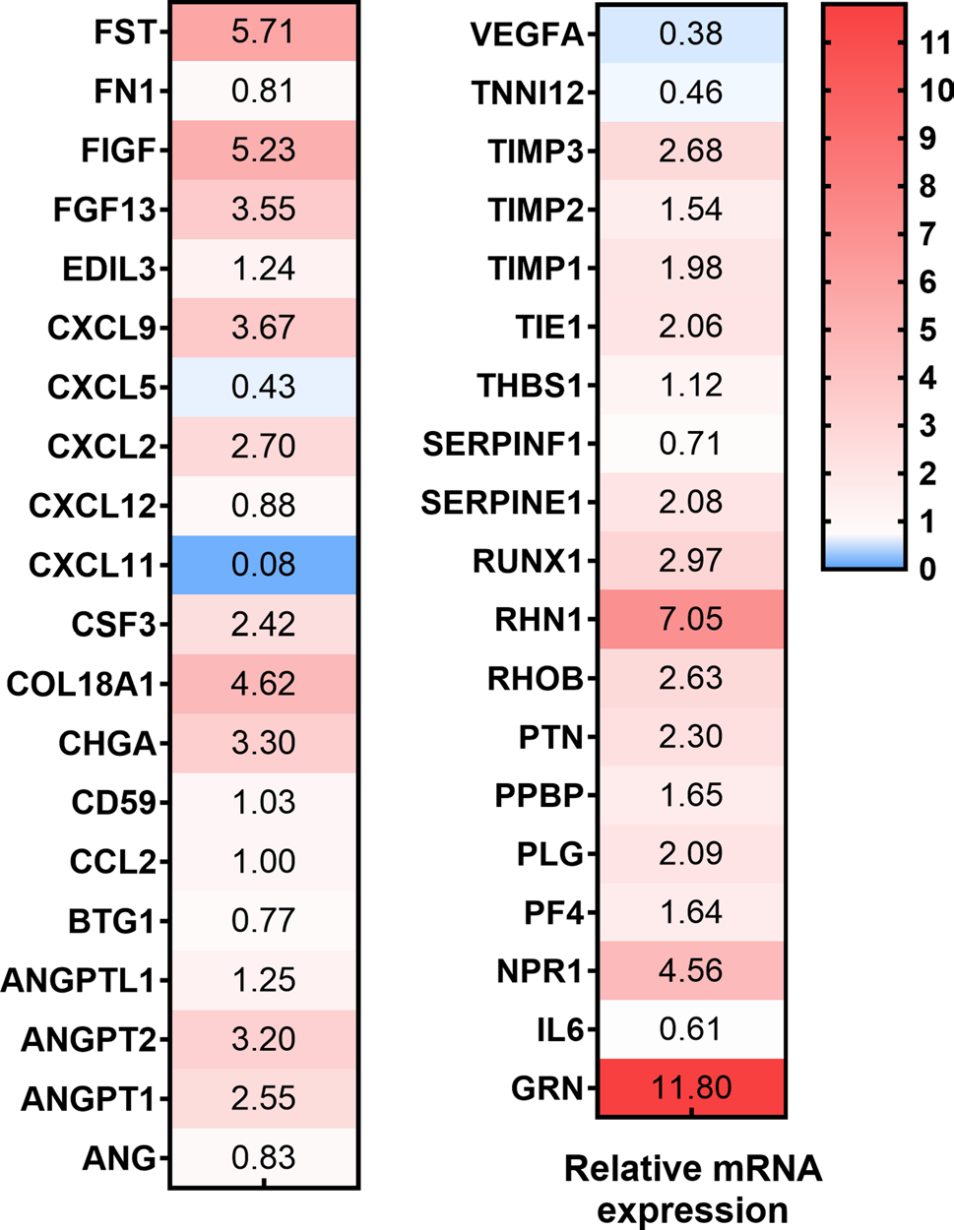
Transcriptomic analysis of MMA-derived MCA samples. Heatmap representation of gene expression profiles of MMA-derived MCA samples. The relative mRNA expression level was calculated by the 2^−ΔΔCt^ comparative method using β2M and GAPDH as housekeeping genes. Control MCA specimens were chosen as the calibrator samples. The color scale distinguishes gene expression values resulting as up-regulated (2 − ΔΔCt > 2; red), unmodulated (white), and down-regulated (2 − ΔΔCt < 0.5; blue), respectively

Gene expression analysis was performed by UO1 in MCA samples from 10 MMA patients and 5 CTRL subjects. These analyses allowed to determine a peculiar gene expression profile in MCA samples from rCVD patients. In particular, a deregulated gene expression of several angiogenic growth factors and angiogenesis inhibitors was observed. Specifically, 39 out of 84 analyzed genes (shown in Fig. 4) were found to be expressed at a detectable level in MCA tissue samples. Of these, four genes were found to be significantly down-regulated (2^−ΔΔCt^ < 0.5) (CXCL5, CXCL11, VEGFA and TNNI12), while 20 transcripts showed a significant up-regulation of their expression (2^−ΔΔCt^ > 2).

#### Plasma proteomic analysis

Proteomic analyses were performed by UO2 on plasma collected from MMA patients (n = 24) compared with age/sex matched HD (n = 25). A number of circulating proteins, such as Growth/differentiation factor 8 (GDF 8), Brevican core protein (BCAN), Carboxypeptidase A2 (CPA2), Epiregulin (EREG), Repulsive guidance molecule A and B (RGMA, RGMB) and Neurocan core protein (NCAN) were differentially expressed between MMA patients and HD (*data not shown, validation analyses are underway).*

## Discussion

In the present study, we reported the preliminary results of the ALIGNED project, a large Italian network on rCVDs aimed at enhancing the currently fragmented diagnostic pathway for these disorders and promoting the development of a multidisciplinary and multi-centric model of care for rCVD patients. The patient recruitment is still ongoing (we collected so far 308 rCVD patients), therefore the results of patients’ data and complete molecular analysis data will be published separately.

Specifically, herein we reported the results of a survey among all participating centers to assess routine rCVDs management and the availability diagnostic tools at each site. We obtained a large adherence to the survey completion since 89.7% fulfilled the survey. Although the number of participating centers represents only a sample and may not fully capture all Italian rCVDs care centers, the wide coverage enhanced by the combination of web-based surveys and site visits provides a useful representative overview of the capabilities of Italian centers in providing rCVD diagnosis and care. The interrater agreement between online collected data and on-site visits supports the consistency and validity of the results of the survey.

The survey results indicate that rCVD management remains highly heterogeneous across centers, with important gaps between hospitals in Northern and Southern regions. Differences are particularly evident in genetic screening for less common rCVDs, such as Fabry disease (54.8% *vs* 30.8%) and HTRA1 (41.9% *vs* 23.1%), whereas for more common genetic screening (e.g., CADASIL or COL4A1/A2) is comparable or even slightly higher in Southern centers.

Moreover, whether extra-neurological consultations are available in almost every center, exceptions include access to nutritionists and neuropsychological evaluations that are more frequent in norther-central centers. Advanced neuroimaging techniques, including BOLD (Blood Oxygenation Level Dependent) and ASL (Arterial Spin Labeling) MRI, brain CT perfusion, cerebral diagnostic angiography, brain SPECT imaging with acetazolamide challenge and transcranial doppler studies, are mainly performed in Northern centers compared with Southern ones, enhancing how research programs from rCVDs are more developed in Northern areas. Furthermore, survey analysis showed that Northern regions tend to provide also more specific and standardized diagnostic and therapeutic pathways, compared with Southern centers.

The discrepancy in the management of rCVDs observed between Northern and Southern region could reflect the uneven distribution of socioeconomic resources and different quality in health care, including stroke, among regions. A study on the coverage of first and second level stroke unit in the national territory showed a shortage of dedicated beds to cerebrovascular patients in central and Southern Italy [19].

Data from the Global Burden of Diseases, Injuries, and Risk Factors Study 2021 showed that Northern regions were observed to have better outcomes life expectancy and disability, reflecting systemic challenges in health care access and quality for Southern patients [1, 20]. This discrepancy is probably even higher in the field of rCVDs, that are often considered orphan, since poorly recognized.

Although our findings need to be validated through more extensive surveys, ideally supported by national programs and scientific societies (e.g., ISA-AAII, SIN), they underscore the need to improve and harmonize the quality of care for cerebrovascular diseases, including rCVDs. Particularly, our preliminary results, could enhance the areas of intervention which require investment and implementation by stakeholders.

Interestingly, preliminary transcriptomic analyses performed on MMA tissue specimens revealed a modulation of several angiogenic growth factors and angiogenesis inhibitors. Among the downregulated genes, CXCL5 and CXCL11 regulate immune responses to cerebrovascular injury [21, 22]. VEGFA contributes to stroke by increasing vascular permeability and blood–brain barrier (BBB) disruption [23], while TNNI2 reflects systemic inflammation by exacerbating stroke outcomes [24]. Regarding the most upregulated gene, GRN encodes a potent anti‑inflammatory and neuroprotective factor (progranulin), which suppresses microglial activation and cytokine release [25]. Furthermore, progranulin also supports angiogenesis by promoting vascular remodeling in ischemic tissues [26].

Moreover, the preliminary investigation of MMA plasma proteome indicated a differential expression of proteins associated with vascular remodeling and endothelial dysfunction. Of note, proteomic analysis of a larger number of plasma and CSF samples -collected from all the enrolled rCVD patients- is currently undergoing, and it will provide a snapshot of the molecular alterations related to the pathogenesis of the rCVDs. Accordingly, a dedicated database has facilitated the collection of biological and clinical data of the patients, in order to accelerate the research on these diseases, to understand the clinical history of rCVD and to enable the enrolling in clinical trials for new and more suitable treatment options. The integration of a strongly phenotype-oriented approach with experimental data originated by innovative and highly sensitive/specific multi-omic techniques will maximize the chance of identifying the most critical biological disease drivers to develop patient-specific predictive models useful for tailored interventions. Molecular profiling will contribute to discovering putative biomarkers with the ultimate goal of identifying novel therapeutic targets/new targeted molecules to limit rCVD progression.

## Limitations

Although the survey provided valuable insights into the effective relevance of Italian centers dedicated to rCVDs, several limitations should be recognized. First, the representativeness of the sample is limited. Not all Italian regions were included, as no hospitals from Piemonte, Valle d’Aosta, Trentino Alto Adige, Veneto, Marche (Northern Italy), nor from Molise, Calabria and Basilicata (Southern Italy), jointed the ALIGNED project. This geographical gap limits the generalizability of the findings to the entire country. Moreover, the participating centers may represent a more engaged and higher-performing subset, thus introducing a potential bias and limiting the extent to which our results reflect the broader national context.

Second, the number of centers from Northern Italy was markedly higher compared to those from the South. This may have introduced a selection bias, whereby only a limited number of renowned neurological centers participated in the survey, while smaller or more isolated centers may not have been recruited. In addition, the observed discrepancy between the availability of genetic consultations and of genetic testing may be explained by the different local organization. In many cases, hospitals rely on external laboratories or they are able to perform locally the genetic analysis but in the absence of a specific diagnostic pathway, including genetic counseling and multidisciplinary evaluation. Moreover, since our sample included only a few centers from Southern regions, it is possible that we arbitrarily enrolled larger or better-equipped institutions, which may have influenced the observed screening rates. This imbalance makes it difficult to provide a true comparison between the two areas. As a result, it is likely that more peripheral or remote centers were not sufficiently reached, which could influence the generalizability of our findings.

## Conclusions

Overall, the ALIGNED survey highlights a substantial variability in the quality of rCVDs management and in the availability of diagnostic tools across Italian centers. Our findings confirm that expertise and resources are mostly located in the Central-Northern regions of Italy, likely due to a higher volume of assisted cases, greater clinical experience, and the wider availability of advanced diagnostic techniques, such as neuroimaging [8, 1, 20]. Mapping the actual distribution of expertise and facilities in Italy is an important step toward addressing existing gaps by: (i) optimizing local resources and points of strategic and effective intervention at regional and national level, (ii) standardizing diagnostic and therapeutic approaches to rCVDs and (iii) ensuring equitable access to high-quality care for all patients, regardless of geographical setting. Future quality measurement and improvement efforts should account for these structural differences when designing efficient rCVD systems of care. Procedural efforts of data sharing and the adoption of decision support system like periodic teleconsultations with expert panels (Virtual Hospitals) could further enhance awareness of unmet needs and support clinical decision-making. The ALIGNED project, in this regard, has the potential to improve training and decision-making processes at local level, and empower stroke centers in the management of rCVDs. Moreover, telehealth solutions, such as the NOVHO project [11], are increasingly recognized as valuable resources across Italy and may contribute to reducing healthcare costs. Indeed, our project may reduce unnecessary travel, and foster the implementation of advanced local care.

## Supplementary Information

Below is the link to the electronic supplementary material.

Supplementary file1 (PDF 765 KB)

Supplementary file2 (PDF 818 KB)

Supplementary file3 (PDF 893 KB)

Supplementary file4 (PDF 949 KB)

Supplementary file5 (PDF 848 KB)

Supplementary file6 (PDF 65.2 KB)

Supplementary file7 (PDF 290 KB)

Supplementary file8 (PDF 193 KB)

Supplementary file9 (PDF 182 KB)

Supplementary file10 (PDF 292 KB)

## Data Availability

The datasets generated and/or analysed during the current study are available from the corresponding author on reasonable request.
