# Supplementary material for: ALIGNED Network for rare cerebrovascular diseases: methodology and preliminary results
Source: Neurol Sci. 2026 Jun 22;47(7):584. doi: 10.1007/s10072-026-09183-1 (PMC13287270; doi:10.1007/s10072-026-09183-1)
Supplement: Supplementary file 2 — Supplementary file2 (PDF 818 KB) [file 10072_2026_9183_MOESM2_ESM.pdf]

Data Dictionary Codebook

04-10-2025 14:38

#	Variable / Field Name	Field Label *Field Note*	Field Attributes (Field Type, Validation, Choices, Calculations, etc.)
**Instrument: Anagrafica (anagrafica)**			
1	[record_id]	Record ID	text
2	[nome_ospedale]	Nome Ospedale	dropdown (autocomplete)

1	IRCCS ISNB UOC Neurologia e Rete Stroke- Ospedale Maggiore
2	Policlinico Universitario Campus Bio-medico di Roma
3	Ospedale di Pisa
4	Ospedale Apuane di Massa
5	IRCCS ISNB UOC Neuromet
6	Ospedale della Murgia, Altamura (Ba)
7	Ospedale San Francesco - ASL Nuoro
8	AUSL IRCCS di Reggio Emilia
9	Ospedale S. Eugenio ASL ROMA 2
10	Ospedale "Jazzolino" - Azienda Sanitaria Provinciale di Vibo Valentia
11	Fondazione Policlinico Universitario Agostino Gemelli, IRCCS, Roma
12	Udine University Hospital
13	AORN A. Cardarelli, Napoli
14	S.M. Goretti Hospital - Latina
15	Ospedale Vito Fazzi, Lecce
16	AOOR Villa Sofia- Cervello, Palermo
17	Ospedale Di Venere Bari
18	Ospedale Santa Maria delle Croci, Ravenna
19	IRCCS Neurolesi Bonino-Pulejo, Messina. U.O. Neurologia
20	Ospedale Santa Maria della Misericordia, Perugia
21	A.O. San Giovanni Addolorata - Roma
22	Ospedale Careggi -Firenze
23	Ospedale Dimiccoli - Barletta
24	Azienda Ospedaliera Universitaria di Modena
25	AOU G. Martino - Messina
26	SS Filippo & Nicola Hospital - Avezzano (L'Aquila)
27	ASST Papa Giovanni XXIII, Bergamo
28	IRCCS Humanitas Research Hospital, Rozzano

			29 IRCCS Mondino, Pavia<br>30 ASST degli Spedali Civili, Brescia<br>31 ASST Ospedale Maggiore di Crema<br>32 IRCCS Ospedale Policlinico San Martino di Genova<br>33 ASST Lariana<br>34 IRCCS Policlinico San Matteo, Pavia<br>35 Policlinico Tor Vergata, UOSD Stroke Unit<br>36 Ospedale Morgagni-Pierantoni, Forlì<br>37 Ospedale Bufalini, Cesena<br>38 PO Levante Asl 2 Savonese- Ospedale San Paolo Savona<br>39 ASST Rhodense<br>40 Ospedale Sant'Andrea, La Spezia<br>41 Fondazione IRCCS Ca' Granda Ospedale Maggiore Policlinico di Milano<br>42 Castrovillari ASP- Cosenza<br>43 Ospedale San Gerardo- Monza<br>44 Ospedale Sandro Pertini - ASL Roma2<br>45 Ospedale "Spaziani" di Frosinone<br>46 Ospedale di Pescara<br>47 Ospedale Luigi Sacco, ASST Fatebenefratelli Sacco<br>48 Fondazione IRCCS "Casa Sollievo della Sofferenza" - San Giovanni Rotondo (FG)<br>49 ASST Melegnano Martesana<br>50 Fondazione Istituto G. Giglio, Cefalù<br>51 ASST di Cremona<br>52 Ospedale Regionale Generale "F. Miulli", Acquaviva delle Fonti (BA)<br>53 ASST Grande Ospedale Metropolitano Niguarda<br>54 IRCCS Istituto Neurologico Carlo Besta
3	[spec_ospedale]<br>Show the field ONLY if:<br>[nome_ospedale] = '4'	Specificare	text
4	[mese_nascita]	Mese di nascita	dropdown<br>1 Gennaio<br>2 Febbraio<br>3 Marzo<br>4 Aprile<br>5 Maggio<br>6 Giugno<br>7 Luglio

			8 Agosto<br>9 Settembre<br>10 Ottobre<br>11 Novembre<br>12 Dicembre
5	[anno_nascita]	Anno di nascita	text (integer, Min: 1924)
6	[data_visita]	Data visita<br>*Si intende la prima visita per sospetta patologia cerebrovascolare presso il centro di riferimento e che ha implicato la raccolta dei dati (può quindi non coincidere con la data di compilazione del presente form)*	text (date_dmy)
7	[data_diagnosi]	Data di diagnosi<br>*Qualora non sia possibile datare con esattezza né il giorno né il mese della diagnosi, si utilizzi convenzionalmente la data 2 luglio dell'anno della diagnosi.*	text (date_dmy)
8	[eta_reclutamento]	Età al reclutamento<br>*Si intende l'età al momento in cui il paziente è giunto all'attenzione del centro per accertamenti in merito a patologia cerebrovascolare e si è provveduto a valutazione medica con raccolta dei dati inseriti in REDCap.*	text (integer)
9	[sesso]	Sesso	radio<br>1 F<br>2 M<br><br>Custom alignment: RH
10	[regione_nascita]	Regione di nascita	dropdown<br>1 Abruzzo<br>2 Basilicata<br>3 Calabria<br>4 Campania<br>5 Emilia Romagna<br>6 Friuli Venezia Giulia<br>7 Lazio<br>8 Liguria<br>9 Lombardia<br>10 Marche<br>11 Molise<br>12 Piemonte<br>13 Puglia<br>14 Sardegna<br>15 Sicilia<br>16 Toscana<br>17 Trentino Alto Adige<br>18 Umbria<br>19 Val d'Aosta<br>20 Veneto<br><br>Custom alignment: RH
11	[regione_residenza]	Regione di residenza	dropdown<br>1 Abruzzo

			2 Basilicata<br>3 Calabria<br>4 Campania<br>5 Emilia Romagna<br>6 Friuli Venezia Giulia<br>7 Lazio<br>8 Liguria<br>9 Lombardia<br>10 Marche<br>11 Molise<br>12 Piemonte<br>13 Puglia<br>14 Sardegna<br>15 Sicilia<br>16 Toscana<br>17 Trentino Alto Adige<br>18 Umbria<br>19 Val d'Aosta<br>20 Veneto<br><br>Custom alignment: RH
12	[etnia]	Gruppo etnico	dropdown<br>1 White<br>2 Black or African American<br>3 Asian<br>4 Other<br><br>Custom alignment: RH
13	[tipo_visita]	Tipo di visita	radio<br>1 Valutazione reparto<br>2 Controllo ambulatoriale
14	[scolarita]	Scolarità<br>*Numero di anni*	text (integer)
15	[anagrafica_complete]	Section Header: *Form Status*<br>Complete?	dropdown<br>0 Incomplete<br>1 Unverified<br>2 Complete
**Instrument: Evento Indice (evento_indice)**			
16	[paziente_asintomatico]	Paziente asintomatico	radio<br>1 Sì<br>0 No<br><br>Custom alignment: RH
17	[data_evento]	Data evento Per uniformità di compilazione, con "evento indice" si è deciso di considerare la condizione medica che ha indotto all'esecuzione di RMN encefalo. Se quindi il paziente presenta	text (date_dmy)

		cefalea dal 2018,ma ha eseguito RMN encefalo nel 2021 per un TIA, l'evento indice è da ritenersi il TIA e la data dell'evento il 2021. La cefalea verrà inserita nel paragrafo successivo "sintomi e segni associati". Qualora non sia possibile datare con esattezza il giorno dell'evento indice, si utilizzi convenzionalmente il giorno 15 del mese in cui l'evento sarebbe occorso; qualora non sia possibile datare con esattezza né il giorno né il mese dell'evento indice, si utilizzi convenzionalmente la data 2 luglio.	
18	[evento_indice]	Tipo di evento indice neurologico	dropdown<br>1 TIA<br>2 Ictus ischemico<br>3 Emorragia cerebrale<br>4 Decadimento cognitivo<br>5 Epilessia<br>6 Cefalea<br>7 Altro
19	[spec_evento_indice]<br>Show the field ONLY if:<br>[evento_indice] = '7'	Specificare	text
20	[nihss_acuta]	NIHSS in fase acuta	text
21	[mrs_prima]	Modified Rankin Scale (MRS) prima dell'evento indice	dropdown<br>0 0: Nessun sintomo<br>1 1: Nessuna inabilità significativa, pur manifestando sintomi: svolge ogni funzione e attività usuali<br>2 2: Leggera inabilità: incapace di svolgere tutte le attività precedenti, ma capace di occuparsi di sé senza assistenza<br>3 3: Inabilità moderata: richiede aiuto, ma cammina senza assistenza<br>4 4: Inabilità moderatamente severa: cammina con assistenza e necessità di assistenza per i propri bisogni corporei<br>5 5: Inabilità severa: allettamento, incontinenza, totalmente dipendente<br>6 6: Deceduto
22	[mrs_valutazione]	Modified Rankin Scale (MRS) alla valutazione	dropdown<br>0 0: Nessun sintomo<br>1 1: Nessuna inabilità significativa, pur manifestando sintomi: svolge ogni funzione e attività usuali<br>2 2: Leggera inabilità: incapace di svolgere tutte le attività precedenti, ma capace di occuparsi di sé senza assistenza<br>3 3: Inabilità moderata: richiede aiuto, ma cammina senza assistenza

			4 | 4: Inabilità moderatamente severa: cammina con assistenza e necessità di assistenza per i propri bisogni corporei<br>5 | 5: Inabilità severa: allettamento, incontinenza, totalmente dipendente<br>6 | 6: Deceduto
23	[eta_esordio]	Età all'esordio	text (integer)
24	[altri_eventi]	Altri eventi Per uniformità di compilazione, con "altri precedenti" si è deciso di considerare esclusivamente gli eventi neurologici acuti, quali TIA, ictus ischemico, emorragia cerebrale, occorsi prima, durante o dopo l'evento indice. Se ad esempio il paziente avesse avuto un TIA a gennaio 2021, quindi una emorragia cerebrale a maggio 2022, e si fosse giunti a RMN diagnostica a seguito di quest'ultima, "TIA" sarà da intendersi quale "altro evento".	radio<br>1 | Sì<br>0 | No<br>2 | NA
25	[numero_altri_eventi]<br>Show the field ONLY if:<br>[altri_eventi] = '1'	Se sì, numero di eventi precedenti	radio<br>1 | 1<br>2 | 2<br>3 | 3<br>4 | Più di 3
26	[tipo_altri_eventi]<br>Show the field ONLY if:<br>[altri_eventi] = '1'	Tipo di eventi precedenti	checkbox<br>1 | tipo_altri_eventi___1 | TIA<br>2 | tipo_altri_eventi___2 | Ictus ischemico<br>3 | tipo_altri_eventi___3 | Emorragia cerebrale
27	[evento_indice_complete]	Section Header: *Form Status*<br>Complete?	dropdown<br>0 | Incomplete<br>1 | Unverified<br>2 | Complete
**Instrument: Sintomi e segni associati (sintomi_e_segni_associati)**			
28	[segni_associati]	Per uniformità di compilazione, si è deciso di comprendere quali "eventi e segni associati" le condizioni presenti nel corso della malattia.	radio<br>1 | Visita diretta<br>2 | Dedotto da cartella clinica
29	[cefalea]	Cefalea	radio<br>1 | Sì<br>0 | No<br>Custom alignment: RH
30	[anno_esordio_cefalea]<br>Show the field ONLY if:<br>[cefalea] = '1'	Anno di esordio	text (integer)
31	[tipo_cefalea]<br>Show the field ONLY if:<br>[cefalea] = '1'	Tipo di cefalea	radio<br>1 | Emicrania con aura<br>2 | Emicrania senz'aura<br>3 | Cefalea tensiva

			4 Altro tipo
32	[aneurismi]	Aneurismi	radio<br>1 Sì<br>0 No<br><br>Custom alignment: RH
33	[depressione]	Depressione	radio<br>1 Sì<br>0 No<br><br>Custom alignment: RH
34	[anno_esordio_depre]<br>Show the field ONLY if:<br>[depressione] = '1'	Anno di esordio	text (integer, Min: 1924)
35	[quadro_cognitivo]	Quadro cognitivo	radio<br>1 Cognitivamente integro<br>2 MCI<br>3 Decadimento cognitivo
36	[valutazioni_cogni]	Valutazioni	checkbox<br>1 valutazioni_cogni___1 MMSE<br>2 valutazioni_cogni___2 MOCA
37	[punteggio_mmse]<br>Show the field ONLY if:<br>[valutazioni_cogni(1)] = '1'	Punteggio MMSE<br>*da 0 a 30*	text (integer, Min: 0, Max: 30)<br>Field Annotation: @FORCE-MINMAX
38	[data_mmse]<br>Show the field ONLY if:<br>[valutazioni_cogni(1)] = '1'	Eseguito in data (MMSE)	text (date_dmy)
39	[punteggio_moca]<br>Show the field ONLY if:<br>[valutazioni_cogni(2)] = '1'	Punteggio MOCA<br>*da 0 a 30*	text (integer, Min: 0, Max: 30)<br>Field Annotation: @FORCE-MINMAX
40	[data_moca]<br>Show the field ONLY if:<br>[valutazioni_cogni(2)] = '1'	Eseguito in data (MOCA)	text (date_dmy)
41	[vasoreattivita_cerebrale]	Alterata vasoreattività cerebrale allo studio doppler transcranico?Per alterata vasoreattività cerebrale si intende il riscontro di valori dibreath holding index (BHI) al doppler transcranico inferiori a 0.69	radio<br>1 Si bilateralmente<br>2 Si monolateralmente<br>0 No
42	[cataratta]	Cataratta	radio<br>1 Sì<br>0 No<br><br>Custom alignment: RH
43	[emorragia_retinica]	Emorragia retinica	radio<br>1 Sì

			0 No<br><br>Custom alignment: RH
44	[anomalia_axenfeld_rieger]	Anomalia Axenfeld-Rieger	radio<br>1 Sì<br>0 No<br><br>Custom alignment: RH
45	[nefropatia]	Nefropatia	radio<br>1 Sì<br>0 No<br><br>Custom alignment: RH
46	[crampi_muscolari]	Crampi muscolari	radio<br>1 Sì<br>0 No<br><br>Custom alignment: RH
47	[prolasso_valvola_mitralica]	Prolasso valvola mitralica	radio<br>1 Sì<br>0 No<br><br>Custom alignment: RH
48	[aritmia]	Aritmia	radio<br>1 Sì<br>0 No<br><br>Custom alignment: RH
49	[fenomeno_di_raynaud]	Fenomeno di Raynaud phenomenon	radio<br>1 Sì<br>0 No<br><br>Custom alignment: RH
50	[sintomi_e_segni_associati_complete]	Section Header: *Form Status*<br>Complete?	dropdown<br>0 Incomplete<br>1 Unverified<br>2 Complete
**Instrument: Fattori di Rischio (fattori_di_rischio)**			
51	[fattori_di_rischio]	Fattori di rischio	checkbox<br>1 fattori_di_rischio___1 Ipertensione arteri[illegible]<br>2 fattori_di_rischio___2 Diabete mellito<br>3 fattori_di_rischio___3 Dislipidemia<br>4 fattori_di_rischio___4 Cardiopatia ischemi[illegible] (Attuale o pregress[illegible] angina e/o trattame[illegible] endovascolare coro[illegible]<br>5 fattori_di_rischio___5 Fibrillazione atriale [illegible] considerarsi paros[illegible] persistente o perm[illegible] presente anche se [illegible]

			<table><tr><td></td><td></td><td>una singola occasio durante la vita del p</td></tr><tr><td>6</td><td>fattori_di_rischio___6</td><td>Uso attuale di estroprogestinici (sostitutiva/anticon</td></tr><tr><td>7</td><td>fattori_di_rischio___7</td><td>Abuso di sostanze</td></tr></table>
52	[ipertensione_arteriosa]	Ipertensione arteriosa	radio<br><table><tr><td>1</td><td>Sì</td></tr><tr><td>0</td><td>No</td></tr></table><br>Custom alignment: RH
53	[diabete_mellito]	Diabete mellito	radio<br><table><tr><td>1</td><td>Sì</td></tr><tr><td>0</td><td>No</td></tr></table><br>Custom alignment: RH
54	[dislipidemia]	Dislipidemia	radio<br><table><tr><td>1</td><td>Sì</td></tr><tr><td>0</td><td>No</td></tr></table><br>Custom alignment: RH
55	[cardiopatia_ischemica]	Cardiopatia ischemicaAttuale o pregresso: IMA e/o angina e/o trattamento endovascolare coronarico	radio<br><table><tr><td>1</td><td>Sì</td></tr><tr><td>0</td><td>No</td></tr></table><br>Custom alignment: RH
56	[fibrillazione_atriale]	Fibrillazione atrialeDa considerarsi parossistica, persistente o permanente e presente anche se solo in una singola occasione durante la vita del paziente	radio<br><table><tr><td>1</td><td>Sì</td></tr><tr><td>0</td><td>No</td></tr></table><br>Custom alignment: RH
57	[fumo]	Fumo di sigarettaPer uniformità di compilazione, si definisce "attivo" chi fuma regolarmente ogni giorno (anche una sola sigaretta) oppure ha smesso da meno di 12 mesi. Si considera "non fumatore" chi non ha mai fumato (ESC)	radio<br><table><tr><td>1</td><td>Sì, attivo</td></tr><tr><td>2</td><td>Sì, pregresso</td></tr><tr><td>0</td><td>No</td></tr></table><br>Custom alignment: RH
58	[peso]	Peso<br>*kg*	text (number)
59	[altezza]	Altezza<br>*cm*	text (integer)
60	[bmi]	BMI	text (number)
61	[sedentarieta]	SedentarietàPer "persona sedentaria" si è deciso di utilizzare la definizione dell'Istituto Superiore di Sanità: "è una persona che non fa un lavoro pesante e che, nel tempo libero, non svolge attività fisica moderata o intensa".	radio<br><table><tr><td>1</td><td>Sì</td></tr><tr><td>0</td><td>No</td></tr><tr><td>2</td><td>NA</td></tr></table><br>Custom alignment: RH
62	[estroprogestinici]	Uso attuale di estroprogestinici (sostitutiva/anticoncezionale)	radio<br><table><tr><td>1</td><td>Sì</td></tr></table>

			0 No<br><br>Custom alignment: RH
63	[alcol]	Abuso di alcol (> 3 unità / die per ♂ e > 2 unità / die per ♀)	radio<br>1 Sì, attuale<br>2 Sì, pregresso<br>0 No<br><br>Custom alignment: RH
64	[iperomocisteinemia]	Iperomocisteinemia (omocisteina ≥ 15 micromol/L)	radio<br>1 Sì<br>0 No<br>2 NA<br><br>Custom alignment: RH
65	[abuso_sostanze]	Abuso di sostanze	radio<br>1 Sì<br>0 No<br><br>Custom alignment: RH
66	[riscontro_pfo]	Riscontro di forame ovale pervio (PFO)?Per forame ovale pervio si intende il riscontro di highintensity transient signals allo studio doppler transcranicocon bubble test. Si intenda per grado "lieve" (1-10 microbolle),"moderato" (11-30 microbolle), "severo" (>30 microbolle) inbasale o dopo manovra di Valsalva.	radio<br>0 No<br>1 Si lieve<br>2 Si moderato<br>3 Si severo
67	[fattori_di_rischio_complete]	Section Header: *Form Status*<br>Complete?	dropdown<br>0 Incomplete<br>1 Unverified<br>2 Complete
**Instrument: Comorbidità (comorbidit)**			
68	[deficit_coagulazione]	Deficit di coagulazione	radio<br>1 Sì<br>0 No<br>2 NA<br><br>Custom alignment: RH
69	[deficit_proteina_c]	Deficit di proteina C	radio<br>1 Sì<br>0 No<br>2 NA<br><br>Custom alignment: RH
70	[deficit_proteina_s]	Deficit di proteina S	radio<br>1 Sì<br>0 No<br>2 NA<br><br>Custom alignment: RH

71	[malattia_autoimmune]	Malattia autoimmune	radio<br>1 Sì<br>0 No<br><br>Custom alignment: RH
72	[quale_malattia_autoimm]<br><br>Show the field ONLY if:<br>[malattia_autoimmune] = '1'	Quale	text
73	[mav_angioma_cavernoso] 	MAV, angioma cavernoso	radio<br>1 Sì<br>0 No<br><br>Custom alignment: RH
74	[comorbidit_complete]	Section Header: *Form Status*<br><br>Complete?	dropdown<br>0 Incomplete<br>1 Unverified<br>2 Complete
**Instrument: Familiarità (familiarit)**			
75	[col4a1]	COL4A1	radio<br>1 Sì<br>0 No<br><br>Custom alignment: RH
76	[parentela_col4a1]<br><br>Show the field ONLY if:<br>[col4a1] = '1'	Grado di parentela	checkbox<br>1 parentela_col4a1___1 Madre<br>2 parentela_col4a1___2 Padre<br>3 parentela_col4a1___3 Zio o zia o zii materni<br>4 parentela_col4a1___4 Zio o zia o zii paterni<br>5 parentela_col4a1___5 Nonna o nonno o nonni materni<br>6 parentela_col4a1___6 Nonna o nonno o nonni paterni<br>7 parentela_col4a1___7 fratello o sorella o fratelli
77	[ictus_ischemico]	Section Header:<br><br>Ictus ischemico	radio<br>1 Sì<br>0 No<br><br>Custom alignment: RH
78	[parentela_ictus_ische]<br><br>Show the field ONLY if:<br>[ictus_ischemico] = '1'	Grado di parentela	checkbox<br>1 parentela_ictus_ische___1 Madre<br>2 parentela_ictus_ische___2 Padre<br>3 parentela_ictus_ische___3 Zio o zia o zii materni

			4 parentela_ictus_ische___4 Zio o zia o zii paterni<br>5 parentela_ictus_ische___5 Nonna o nonno o nonni materni<br>6 parentela_ictus_ische___6 Nonna o nonno o nonni paterni<br>7 parentela_ictus_ische___7 fratello o sorella o fratelli
79	[ictus_emorragico]	Section Header:<br>Ictus emorragico	radio<br>1 Sì<br>0 No<br><br>Custom alignment: RH
80	[parentela_ictus_emorr]<br><br>Show the field ONLY if:<br>[ictus_emorragico] = '1'	Grado di parentela	checkbox<br>1 parentela_ictus_emorr___1 Madre<br>2 parentela_ictus_emorr___2 Padre<br>3 parentela_ictus_emorr___3 Zio o zia o zii materni<br>4 parentela_ictus_emorr___4 Zio o zia o zii paterni<br>5 parentela_ictus_emorr___5 Nonna o nonno o nonni materni<br>6 parentela_ictus_emorr___6 Nonna o nonno o nonni paterni<br>7 parentela_ictus_emorr___7 fratello o sorella o fratelli
81	[cefalea_emicrania]	Section Header:<br>Cefalea / emicrania con o senza aura	radio<br>1 Sì<br>0 No<br><br>Custom alignment: RH
82	[parentela_cefalea]<br><br>Show the field ONLY if:<br>[cefalea_emicrania] = '1'	Grado di parentela	checkbox<br>1 parentela_cefalea___1 Madre<br>2 parentela_cefalea___2 Padre<br>3 parentela_cefalea___3 Zio o zia o zii materni<br>4 parentela_cefalea___4 Zio o zia o zii paterni<br>5 parentela_cefalea___5 Nonna o nonno o nonni materni

			6 | parentela_cefalea___6 | Nonna o nonno o nonni paterni<br>7 | parentela_cefalea___7 | fratello o sorella o fratelli
83	[decadimento_cognitivo]	Section Header:<br>Decadimento cognitivo	radio<br>1 | Sì<br>0 | No<br><br>Custom alignment: RH
84	[parentela_decadimento]<br><br>Show the field ONLY if:<br>[decadimento_cognitivo] = '1'	Grado di parentela	checkbox<br>1 | parentela_decadimento___1 | Madre<br>2 | parentela_decadimento___2 | Padre<br>3 | parentela_decadimento___3 | Zio o zia o zii materni<br>4 | parentela_decadimento___4 | Zio o zia o zii paterni<br>5 | parentela_decadimento___5 | Nonna o nonno o nonni materni<br>6 | parentela_decadimento___6 | Nonna o nonno o nonni paterni<br>7 | parentela_decadimento___7 | fratello o sorella o fratelli
85	[disturbo_psichiatrico]	Section Header:<br>Disturbo psichiatrico	radio<br>1 | Sì<br>0 | No<br><br>Custom alignment: RH
86	[parentela_dist_psic]<br><br>Show the field ONLY if:<br>[disturbo_psichiatrico] = '1'	Grado di parentela	checkbox<br>1 | parentela_dist_psic___1 | Madre<br>2 | parentela_dist_psic___2 | Padre<br>3 | parentela_dist_psic___3 | Zio o zia o zii materni<br>4 | parentela_dist_psic___4 | Zio o zia o zii paterni<br>5 | parentela_dist_psic___5 | Nonna o nonno o nonni materni<br>6 | parentela_dist_psic___6 | Nonna o nonno o nonni paterni<br>7 | parentela_dist_psic___7 | fratello o sorella o fratelli

87	[epilessia]	Section Header:<br>Epilessia	radio<br>1 Sì<br>0 No<br><br>Custom alignment: RH
88	[parentela_epilessia]<br><br>Show the field ONLY if:<br>[epilessia] = '1'	Grado di parentela	checkbox<br>1 parentela_epilessia___1 Madre<br>2 parentela_epilessia___2 Padre<br>3 parentela_epilessia___3 Zio o zia o zii materni<br>4 parentela_epilessia___4 Zio o zia o zii paterni<br>5 parentela_epilessia___5 Nonna o nonno o nonni materni<br>6 parentela_epilessia___6 Nonna o nonno o nonni paterni<br>7 parentela_epilessia___7 fratello o sorella o fratelli
89	[familiarit_complete]	Section Header: *Form Status*<br>Complete?	dropdown<br>0 Incomplete<br>1 Unverified<br>2 Complete
**Instrument: Terapia Medica (terapia_medica)**			
90	[acido_acetilsalicilico]	Acido acetilsalicilico	radio<br>1 Sì<br>0 No<br><br>Custom alignment: RH
91	[specifica_acido_acetil]<br><br>Show the field ONLY if:<br>[acido_acetilsalicilico] = '1'	Specifica	text
92	[clopidogrel]	Section Header:<br>Clopidogrel	radio<br>1 Sì<br>0 No<br><br>Custom alignment: RH
93	[altro_antiaggregante]	Section Header:<br>Altro antiaggregante	radio<br>1 Sì<br>0 No<br><br>Custom alignment: RH
94	[tipo_antiaggreg]<br><br>Show the field ONLY if:<br>[altro_antiaggregante] = '1'	Tipo	text

95	[dose_antiaggreg]<br>Show the field ONLY if: [altro_antiaggregante] = '1'	Dose	text (number)
96	[antipertensivi]	Section Header:<br>Antipertensivi	radio<br>1 Sì<br>0 No<br>Custom alignment: RH
97	[tipo_antipertens]<br>Show the field ONLY if: [antipertensivi] = '1'	Tipo	text
98	[dose_antipertens]<br>Show the field ONLY if: [antipertensivi] = '1'	Dose	text (number)
99	[nao]	Section Header:<br>NAO	radio<br>1 Sì<br>0 No<br>Custom alignment: RH
100	[nome_nao]<br>Show the field ONLY if: [nao] = '1'	Specificare il nome	text
101	[tao]	Section Header:<br>TAO	radio<br>1 Sì<br>0 No<br>Custom alignment: RH
102	[altro_anticoagulante]	Section Header:<br>Altro anticoagulante	radio<br>1 Sì<br>0 No<br>Custom alignment: RH
103	[statina]	Section Header:<br>Statina	radio<br>1 Sì<br>0 No<br>Custom alignment: RH
104	[tipo_statina]<br>Show the field ONLY if: [statina] = '1'	Tipo	text
105	[dose_statina]<br>Show the field ONLY if: [statina] = '1'	Dose	text (number)
106	[antiepilettico]	Section Header:<br>Antiepilettico	radio<br>1 Sì<br>0 No<br>Custom alignment: RH
107	[tipo_antipilettico]	Tipo	text

	Show the field ONLY if: [antiepilettico] = '1'		
108	[dose_antipilettico]<br>Show the field ONLY if: [antiepilettico] = '1'	Dose	text (number)
109	[ssri]	Section Header: SSRI	radio<br>1 Sì<br>0 No<br>Custom alignment: RH
110	[tipo_ssri]<br>Show the field ONLY if: [ssri] = '1'	Tipo	text
111	[dose_ssri]<br>Show the field ONLY if: [ssri] = '1'	Dose	text (number)
112	[snri]	Section Header: SNRI	radio<br>1 Sì<br>0 No<br>Custom alignment: RH
113	[tipo_snri]<br>Show the field ONLY if: [snri] = '1'	Tipo	text
114	[dose_snri]<br>Show the field ONLY if: [snri] = '1'	Dose	text (number)
115	[triptano]	Section Header: Triptano	radio<br>1 Sì<br>0 No<br>Custom alignment: RH
116	[tipo_triptano]<br>Show the field ONLY if: [triptano] = '1'	Tipo	text
117	[dose_triptano]<br>Show the field ONLY if: [triptano] = '1'	Dose	text (number)
118	[profilassi_cefalea_cron]	Section Header: Profilassi per cefalea cronica?	radio<br>1 Sì<br>0 No<br>Custom alignment: RH
119	[tipo_profilassi]<br>Show the field ONLY if: [profilassi_cefalea_cron] = '1'	Tipo	text
120	[dose_profilassi]<br>Show the field ONLY if:	Dose	text (number)

	[profilassi_cefalea_cron] = '1'		
121	[terapia_medica_complete]	Section Header: *Form Status*<br>Complete?	dropdown<br>0 Incomplete<br>1 Unverified<br>2 Complete

Instrument: MRI Encefalo (mri_encefalo)

122	[data_mri]	Section Header: *Si intende l'ultima RMN encefalo disponibile al momento della valutazione*<br>Data di esecuzione	text (date_dmy)
123	[tipo_rmn]	Tipo di RMN	radio<br>1 1 Tesla<br>2 1.5 Tesla<br>3 3 Tesla<br>Custom alignment: RH
124	[sequenze_rmn]	Sequenze presenti nella RMN	checkbox<br>1 sequenze_rmn___1 T2<br>2 sequenze_rmn___2 SWI<br>3 sequenze_rmn___3 FLAIR<br>Custom alignment: RH
125	[rmn_encefalo]	RMN encefalo	checkbox<br>1 rmn_encefalo___1 Emorragia lobare (l-ICH)<br>2 rmn_encefalo___2 Microemorragie lobari (CMBs)<br>3 rmn_encefalo___3 Iperintenistà della sostanza bianca multifocali (White Matter Hyperintensities in a multispot pattern)<br>4 rmn_encefalo___4 Siderosi corticale superficiale (CSS)<br>5 rmn_encefalo___5 Spazi perivascolari del centro semiovale in numero > 20 (CSP-PVSs)<br>6 rmn_encefalo___6 Lacune ischemiche<br>7 rmn_encefalo___7 Restrizione in DWI
126	[rmn_libera_emorr]	La RMN è libera da emorragie in sede profonda?	radio<br>1 Sì<br>0 No<br>Custom alignment: RH
127	[svd_score]	SVD score	radio<br>0 0<br>1 1

			2 2<br>3 3<br>4 4<br><br>Custom alignment: RH
128	[dwi]	DWI	radio<br>1 Focale<br>2 Disseminata<br><br>Custom alignment: RH
129	[mri_encefalo_complete]	Section Header: *Form Status*<br>Complete?	dropdown<br>0 Incomplete<br>1 Unverified<br>2 Complete
**Instrument: Angiografia Encefalo (angiografia_encefalo)**			
130	[data_angiografia]	Section Header: *Si intende l'ultima angiografia encefalo disponibile al momento della valutazione*<br>Data di esecuzione	text (date_dmy)
131	[angiografia_encefalo]	Angiografia encefalo	checkbox<br>1 angiografia_encefalo___1 Stenosi<br>2 angiografia_encefalo___2 Occlusione M1<br>3 angiografia_encefalo___3 Network di vasi collaterali
132	[specifica_stenosi]<br>Show the field ONLY if: [angiografia_encefalo(1)] = '1'	Specifica Stenosi	checkbox<br>1 specifica_stenosi___1 M1<br>2 specifica_stenosi___2 M2<br>3 specifica_stenosi___3 M3<br>4 specifica_stenosi___4 ACA<br>5 specifica_stenosi___5 ACP<br>6 specifica_stenosi___6 ACoA<br>7 specifica_stenosi___7 ACoP<br>8 specifica_stenosi___8 BA<br>9 specifica_stenosi___9 V<br>10 specifica_stenosi___10 SCA<br>11 specifica_stenosi___11 AICA<br>12 specifica_stenosi___12 PICA
133	[specifica_stenosi_m1]<br>Show the field ONLY if: [specifica_stenosi(1)] = '1'	Specifica Stenosi M1	radio<br>1 Dx<br>2 Sx<br><br>Custom alignment: RH
134	[specifica_occlusione]<br>Show the field ONLY if: [angiografia_encefalo(2)] = '1'	Specifica Occlusione	checkbox<br>1 specifica_occlusione___1 M1<br>2 specifica_occlusione___2 M2<br>3 specifica_occlusione___3 M3

			4 specifica_occlusione___4 ACA<br>5 specifica_occlusione___5 ACP<br>6 specifica_occlusione___6 ACoA<br>7 specifica_occlusione___7 ACoP<br>8 specifica_occlusione___8 BA<br>9 specifica_occlusione___9 V<br>10 specifica_occlusione___10 SCA<br>11 specifica_occlusione___11 AICA<br>12 specifica_occlusione___12 PICA
135	[angiografia_encefalo_complete]	Section Header: *Form Status*<br>Complete?	dropdown<br>0 Incomplete<br>1 Unverified<br>2 Complete
