# Supplementary material for: ALIGNED Network for rare cerebrovascular diseases: methodology and preliminary results
Source: Neurol Sci. 2026 Jun 22;47(7):584. doi: 10.1007/s10072-026-09183-1 (PMC13287270; doi:10.1007/s10072-026-09183-1)
Supplement: Supplementary file 3 — Supplementary file3 (PDF 893 KB) [file 10072_2026_9183_MOESM3_ESM.pdf]

Data Dictionary Codebook

04-10-2025 14:38

#	Variable / Field Name	Field Label<br>*Field Note*	Field Attributes (Field Type, Validation, Choices, Calculations, etc.)
**Instrument: Anagrafica (anagrafica)**			
1	[record_id]	Record ID	text
2	[nome_ospedale]	Nome Ospedale	dropdown (autocomplete)<br>1 IRCCS ISNB UOC Neurologia e Rete Stroke- Ospedale Maggiore<br>2 Policlinico Universitario Campus Bio-medico di Roma<br>3 Ospedale di Pisa<br>4 Ospedale Apuane di Massa<br>5 IRCCS ISNB UOC Neuromet<br>6 Ospedale della Murgia, Altamura (Ba)<br>7 Ospedale San Francesco - ASL Nuoro<br>8 AUSL IRCCS di Reggio Emilia<br>9 Ospedale S. Eugenio ASL ROMA 2<br>10 Ospedale "Jazzolino" - Azienda Sanitaria Provinciale di Vibo Valentia<br>11 Fondazione Policlinico Universitario Agostino Gemelli, IRCCS, Roma<br>12 Udine University Hospital<br>13 AORN A. Cardarelli, Napoli<br>14 S.M. Goretti Hospital - Latina<br>15 Ospedale Vito Fazzi, Lecce<br>16 AOOR Villa Sofia- Cervello, Palermo<br>17 Ospedale Di Venere Bari<br>18 Ospedale Santa Maria delle Croci, Ravenna<br>19 IRCCS Neurolesi Bonino-Pulejo, Messina. U.O. Neurologia<br>20 Ospedale Santa Maria della Misericordia, Perugia<br>21 A.O. San Giovanni Addolorata - Roma<br>22 Ospedale Careggi -Firenze<br>23 Ospedale Dimiccoli - Barletta<br>24 Azienda Ospedaliera Universitaria di Modena<br>25 AOU G. Martino - Messina<br>26 SS Filippo & Nicola Hospital - Avezzano (L'Aquila)<br>27 ASST Papa Giovanni XXIII, Bergamo<br>28 IRCCS Humanitas Research Hospital, Rozzano

			29 IRCCS Mondino, Pavia<br>30 ASST degli Spedali Civili, Brescia<br>31 ASST Ospedale Maggiore di Crema<br>32 IRCCS Ospedale Policlinico San Martino di Genova<br>33 ASST Lariana<br>34 IRCCS Policlinico San Matteo, Pavia<br>35 Policlinico Tor Vergata, UOSD Stroke Unit<br>36 Ospedale Morgagni-Pierantoni, Forlì<br>37 Ospedale Bufalini, Cesena<br>38 PO Levante Asl 2 Savonese- Ospedale San Paolo Savona<br>39 ASST Rhodense<br>40 Ospedale Sant'Andrea, La Spezia<br>41 Fondazione IRCCS Ca' Granda Ospedale Maggiore Policlinico di Milano<br>42 Castrovillari ASP- Cosenza<br>43 Ospedale San Gerardo- Monza<br>44 Ospedale Sandro Pertini - ASL Roma2<br>45 Ospedale "Spaziani" di Frosinone<br>46 Ospedale di Pescara<br>47 Ospedale Luigi Sacco, ASST Fatebenefratelli Sacco<br>48 Fondazione IRCCS "Casa Sollievo della Sofferenza" - San Giovanni Rotondo (FG)<br>49 ASST Melegnano Martesana<br>50 Fondazione Istituto G. Giglio, Cefalù<br>51 ASST di Cremona<br>52 Ospedale Regionale Generale "F. Miulli", Acquaviva delle Fonti (BA)<br>53 ASST Grande Ospedale Metropolitano Niguarda<br>54 IRCCS Istituto Neurologico Carlo Besta
3	[spec_ospedale]<br>Show the field ONLY if:<br>[nome_ospedale] = '4'	Specificare	text
4	[mese_nascita]	Mese di nascita	dropdown<br>1 Gennaio<br>2 Febbraio<br>3 Marzo<br>4 Aprile<br>5 Maggio<br>6 Giugno<br>7 Luglio

			8 Agosto<br>9 Settembre<br>10 Ottobre<br>11 Novembre<br>12 Dicembre
5	[anno_nascita]	Anno di nascita	text (integer, Min: 1924)
6	[data_visita]	Data visita<br>*Si intende la prima visita per sospetta Malattia di Fabry presso il centro di riferimento e che ha implicato la raccolta dei dati (può quindi non coincidere con la data di compilazione del presente form)*	text (date_dmy)
7	[data_diagnosi]	Data di diagnosi<br>*Si intende la data in cui il paziente è stato sottoposto al primo studio neuroradiologico dei vasi intracranici; qualora non sia possibile datare con esattezza né il giorno né il mese della diagnosi, si utilizzi convenzionalmente la data 2 luglio.*	text (date_dmy)
8	[eta_reclutamento]	Età al reclutamento<br>*Si intende l'età al momento in cui il paziente è giunto all'attenzione del centro per accertamenti in merito a malattia di FABRY e si è provveduto a valutazione medica con raccolta dei dati inseriti in REDCap.*	text (integer)
9	[sesso]	Sesso	radio<br>1 F<br>2 M<br><br>Custom alignment: RH
10	[regione_nascita]	Regione di nascita	dropdown<br>1 Abruzzo<br>2 Basilicata<br>3 Calabria<br>4 Campania<br>5 Emilia Romagna<br>6 Friuli Venezia Giulia<br>7 Lazio<br>8 Liguria<br>9 Lombardia<br>10 Marche<br>11 Molise<br>12 Piemonte<br>13 Puglia<br>14 Sardegna<br>15 Sicilia<br>16 Toscana<br>17 Trentino Alto Adige<br>18 Umbria<br>19 Val d'Aosta<br>20 Veneto<br><br>Custom alignment: RH
11	[regione_residenza]	Regione di residenza	dropdown

			1 Abruzzo<br>2 Basilicata<br>3 Calabria<br>4 Campania<br>5 Emilia Romagna<br>6 Friuli Venezia Giulia<br>7 Lazio<br>8 Liguria<br>9 Lombardia<br>10 Marche<br>11 Molise<br>12 Piemonte<br>13 Puglia<br>14 Sardegna<br>15 Sicilia<br>16 Toscana<br>17 Trentino Alto Adige<br>18 Umbria<br>19 Val d'Aosta<br>20 Veneto<br><br>Custom alignment: RH
12	[etnia]	Gruppo etnico	dropdown<br>1 White<br>2 Black or African American<br>3 Asian<br>4 Other<br><br>Custom alignment: RH
13	[tipo_visita]	Tipo di visita	radio<br>1 Valutazione reparto<br>2 Controllo ambulatoriale
14	[scolarita]	Scolarità<br>*Numero di anni*	text (integer)
15	[anagrafica_complete]	Section Header: *Form Status*<br>Complete?	dropdown<br>0 Incomplete<br>1 Unverified<br>2 Complete
**Instrument: Evento Indice (evento_indice)**			
16	[paziente_asintomatico]	Paziente asintomatico	radio<br>1 Sì<br>0 No<br><br>Custom alignment: RH
17	[data_evento]	Data evento Per uniformità di compilazione, con "evento indice" si è deciso di considerare la	text (date_dmy)

		condizione medica che ha indotto all'esecuzione di studio neuroradiologico. Se quindi il paziente presenta cefalea dal 2018, ma ha eseguito RMN encefalo nel 2021 per un TIA, l'evento indice è da ritenersi il TIA e la data dell'evento il 2021. La cefalea verrà inserita nel paragrafo successivo "sintomi e segni associati". Qualora non sia possibile datare con esattezza il giorno dell'evento indice, si utilizzi convenzionalmente il giorno 15 del mese in cui l'evento sarebbe occorso; qualora non sia possibile datare con esattezza né il giorno né il mese dell'evento indice, si utilizzi convenzionalmente la data 2 luglio. Qualora il paziente fosse asintomatico, si prega lasciare vuoto questo campo.	
18	[evento_indice]	Tipo di evento indice	dropdown<br>1 TIA<br>2 Ictus ischemico<br>3 Emorragia cerebrale<br>4 Decadimento cognitivo<br>5 Depressione<br>6 Cefalea con o senza aura<br>7 Sintomi psichiatrici<br>8 Epilessia<br>9 Altro
19	[spec_evento_indice]<br>Show the field ONLY if:<br>[evento_indice] = '9'	Specificare	text
20	[nihss_acuta]	NIHSS in fase acuta	text
21	[mrs_prima]	Modified Rankin Scale (MRS) prima dell'evento indice	dropdown<br>0 0: Nessun sintomo<br>1 1: Nessuna inabilità significativa, pur manifestando sintomi: svolge ogni funzione e attività usuali<br>2 2: Leggera inabilità: incapace di svolgere tutte le attività precedenti, ma capace di occuparsi di sé senza assistenza<br>3 3: Inabilità moderata: richiede aiuto, ma cammina senza assistenza<br>4 4: Inabilità moderatamente severa: cammina con assistenza e necessità di assistenza per i propri bisogni corporei<br>5 5: Inabilità severa: allettamento, incontinenza, totalmente dipendente<br>6 6: Deceduto
22	[mrs_valutazione]	Modified Rankin Scale (MRS) alla valutazione	dropdown<br>0 0: Nessun sintomo<br>1 1: Nessuna inabilità significativa, pur manifestando sintomi: svolge ogni funzione e attività usuali

			2 | 2: Leggera inabilità: incapace di svolgere tutte le attività precedenti, ma capace di occuparsi di sé senza assistenza<br>3 | 3: Inabilità moderata: richiede aiuto, ma cammina senza assistenza<br>4 | 4: Inabilità moderatamente severa: cammina con assistenza e necessità di assistenza per i propri bisogni corporei<br>5 | 5: Inabilità severa: allettamento, incontinenza, totalmente dipendente<br>6 | 6: Deceduto
23	[eta_esordio]	Età all'esordio	text (integer)
24	[altri_eventi]	Altri eventiPer uniformità di compilazione, con "altri eventii" si è deciso di considerare esclusivamente gli eventi neurologici acuti, quali TIA, ictus ischemico, emorragia cerebrale, psicosi, occorsi prima, durante o dopo l'evento indice. Se ad esempio il paziente avesse avuto un TIA a gennaio 2021, quindi una emorragia cerebrale a maggio 2022, e si fosse giunti a RMN diagnostica a seguito di quest'ultima, "TIA" sarà da intendersi quale "altro evento".	radio<br>1 | Sì<br>0 | No<br>2 | NA
25	[numero_altri_eventi]<br>Show the field ONLY if:<br>[altri_eventi] = '1'	Se sì, numero di altri eventi	radio<br>1 | 1<br>2 | 2<br>3 | 3<br>4 | Più di 3
26	[tipo_altri_eventi]<br>Show the field ONLY if:<br>[altri_eventi] = '1'	Tipo di altri eventi	checkbox<br>1 | tipo_altri_eventi___1 | TIA<br>2 | tipo_altri_eventi___2 | Ictus ischemico<br>3 | tipo_altri_eventi___3 | Emorragia cerebrale<br>4 | tipo_altri_eventi___4 | Crisi epilettica
27	[evento_indice_complete]	Section Header: *Form Status*<br>Complete?	dropdown<br>0 | Incomplete<br>1 | Unverified<br>2 | Complete
**Instrument: Sintomi e segni associati (sintomi_e_segni_associati)**			
28	[segni_associati]	Per uniformità di compilazione, si è deciso di comprendere quali "eventi e segni associati" le condizioni presenti nel corso della malattia.	radio<br>1 | Visita diretta<br>2 | Dedotto da cartella clinica
29	[cefalea]	Cefalea	radio<br>1 | Sì<br>0 | No<br><br>Custom alignment: RH
30	[anno_esordio_cefalea]	Anno di esordio	text (integer)

	Show the field ONLY if: [cefalea] = '1'		
31	[tipo_cefalea] Show the field ONLY if: [cefalea] = '1'	Tipo di cefalea	radio<br>1 Emicrania con aura<br>2 Emicrania senz'aura<br>3 Cefalea tensiva<br>4 Altro tipo
32	[patologia_renale]	Patologia renale	radio<br>1 Sì<br>0 No<br>Custom alignment: RH
33	[tipo_patologia_renale] Show the field ONLY if: [patologia_renale] = '1'	Se sì, tipo	text<br>Custom alignment: RH
34	[miocard_dilatativa_aritmia]	Miocardiopatia dilatativa o aritmia	radio<br>1 Sì<br>0 No<br>Custom alignment: RH
35	[cornea_verticillata]	Cornea verticillata	radio<br>1 Sì<br>0 No<br>Custom alignment: RH
36	[angiocheratoma]	Angiocheratoma	radio<br>1 Sì<br>0 No<br>Custom alignment: RH
37	[ipoacusia]	Ipoacusia	radio<br>1 Sì<br>0 No<br>Custom alignment: RH
38	[vertigini]	Vertigini	radio<br>1 Sì<br>0 No<br>Custom alignment: RH
39	[dolori_urenti]	Dolori urenti/ acroparestesie	radio<br>1 Sì<br>0 No<br>Custom alignment: RH
40	[polineuropatia]	Polineuropatia	radio<br>1 Sì<br>0 No

			Custom alignment: RH
41	[neuropatia_piccole_fibre]	Neuropatia piccole fibre	radio<br>1 Sì<br>0 No<br><br>Custom alignment: RH
42	[ipoanidrosi]	Ipoanidrosi	radio<br>1 Sì<br>0 No<br><br>Custom alignment: RH
43	[disturbi_sistema_vegetativo]	Disturbi sistema vegetativo	radio<br>1 Sì<br>0 No<br><br>Custom alignment: RH
44	[depressione]	Depressione	radio<br>1 Sì<br>0 No<br><br>Custom alignment: RH
45	[anno_esordio_depre]<br><br>Show the field ONLY if:<br>[depressione] = '1'	Anno di esordio	text (integer)
46	[quadro_cognitivo]	Quadro cognitivo	radio<br>1 Cognitivamente integro<br>2 MCI<br>3 Decadimento cognitivo
47	[valutazioni_cogni]	Valutazioni	checkbox<br>1 valutazioni_cogni___1 MMSE<br>2 valutazioni_cogni___2 MOCA
48	[punteggio_mmse]<br><br>Show the field ONLY if:<br>[valutazioni_cogni(1)] = '1'	Punteggio MMSE<br>*da 0 a 30*	text (integer, Min: 0, Max: 30)<br>Field Annotation: @FORCE-MINMAX
49	[data_mmse]<br><br>Show the field ONLY if:<br>[valutazioni_cogni(1)] = '1'	Eseguito in data (MMSE)	text (date_dmy)
50	[punteggio_moca]<br><br>Show the field ONLY if:<br>[valutazioni_cogni(2)] = '1'	Punteggio MOCA<br>*da 0 a 30*	text (integer, Min: 0, Max: 30)<br>Field Annotation: @FORCE-MINMAX
51	[data_moca]<br><br>Show the field ONLY if:<br>[valutazioni_cogni(2)] = '1'	Eseguito in data (MOCA)	text (date_dmy)
52	[crisi_comiziale]	Crisi comiziale	radio<br>1 Sì

			0 No<br><br>Custom alignment: RH
53	[tipo_crisi]<br><br>Show the field ONLY if:<br>[crisi_comiziale] = '1'	Tipo crisi	radio<br>1 Motoria<br>2 Non motoria
54	[specifica]<br><br>Show the field ONLY if:<br>[crisi_comiziale] = '1'	Specifica	radio<br>1 Onset focale<br>2 Onset generalizzato<br>3 Unknown onset
55	[anno_esordio_crisi]<br><br>Show the field ONLY if:<br>[crisi_comiziale] = '1'	Anno di esordio	text (integer, Min: 1924)
56	[sintomi_e_segni_associati_complete]	Section Header: *Form Status*<br>Complete?	dropdown<br>0 Incomplete<br>1 Unverified<br>2 Complete
**Instrument: Fattori di Rischio (fattori_di_rischio)**			
57	[fattori_di_rischio]	Fattori di rischio	checkbox<br>1 fattori_di_rischio___1 Ipertensione arteric<br>2 fattori_di_rischio___2 Diabete mellito<br>3 fattori_di_rischio___3 Dislipidemia<br>4 fattori_di_rischio___4 Cardiopatia ischemi (Attuale o pregresso angina e/o trattame endovascolare coro<br>5 fattori_di_rischio___5 Fibrillazione atriale considerarsi paross persistente o perma presente anche se s una singola occasio durante la vita del p<br>6 fattori_di_rischio___6 Uso attuale di estroprogestinici (sostitutiva/anticon<br>7 fattori_di_rischio___7 Abuso di sostanze
58	[ipertensione_arteriosa]	Ipertensione arteriosa	radio<br>1 Sì<br>0 No<br><br>Custom alignment: RH
59	[diabete_mellito]	Diabete mellito	radio<br>1 Sì<br>0 No<br><br>Custom alignment: RH
60	[dislipidemia]	Dislipidemia	radio<br>1 Sì<br>0 No

			Custom alignment: RH
61	[cardiopatia_ischemica]	Cardiopatia ischemicaAttuale o pregresso: IMA e/o angina e/o trattamento endovascolare coronarico	radio<br>1 Sì<br>0 No<br>Custom alignment: RH
62	[fibrillazione_atriale]	Fibrillazione atrialeDa considerarsi parossistica, persistente o permanente e presente anche se solo in una singola occasione durante la vita del paziente	radio<br>1 Sì<br>0 No<br>Custom alignment: RH
63	[fumo]	Fumo di sigarettaPer uniformità di compilazione, si definisce "attivo" chi fuma regolarmente ogni giorno (anche una sola sigaretta) oppure ha smesso da meno di 12 mesi. Si considera "non fumatore" chi non ha mai fumato (ESC)	radio<br>1 Sì, attivo<br>2 Sì, pregresso<br>0 No<br>Custom alignment: RH
64	[peso]	Peso<br>*kg*	text (number)
65	[altezza]	Altezza<br>*cm*	text (number)
66	[bmi]	BMI	text (number)
67	[sedentarieta]	SedentarietàPer "persona sedentaria" si è deciso di utilizzare la definizione dell'Istituto Superiore di Sanità: "è una persona che non fa un lavoro pesante e che, nel tempo libero, non svolge attività fisica moderata o intensa".	radio<br>1 Sì<br>0 No<br>2 NA<br>Custom alignment: RH
68	[estroprogestinici]	Uso attuale di estroprogestinici (sostitutiva/anticoncezionale)	radio<br>1 Sì<br>0 No<br>Custom alignment: RH
69	[alcol]	Abuso di alcol (> 3 unità / die per ♂ e > 2 unità / die per ♀)	radio<br>1 Sì, attuale<br>2 Sì, pregresso<br>0 No<br>Custom alignment: RH
70	[iperomocisteinemia]	Iperomocisteinemia (omocisteina ≥ 15 micromol/L)	radio<br>1 Sì<br>0 No<br>2 NA<br>Custom alignment: RH
71	[abuso_sostanze]	Abuso di sostanze	radio<br>1 Sì<br>0 No

			Custom alignment: RH
72	[riscontro_pfo]	Riscontro di forame ovale pervio (PFO)?Per forame ovale pervio si intende il riscontro di highintensity transient signals allo studio doppler transcranicocon bubble test. Si intenda per grado "lieve" (1-10 microbolle),"moderato" (11-30 microbolle), "severo" (>30 microbolle) inbasale o dopo manovra di Valsalva.	radio<br>0 No<br>1 Si lieve<br>2 Si moderato<br>3 Si severo
73	[fattori_di_rischio_complete]	Section Header: *Form Status*<br>Complete?	dropdown<br>0 Incomplete<br>1 Unverified<br>2 Complete
**Instrument: Comorbidità (comorbidit)**			
74	[deficit_coagulazione]	Deficit di coagulazione	radio<br>1 Sì<br>0 No<br>2 NA<br><br>Custom alignment: RH
75	[deficit_proteina_c]	Deficit di proteina C	radio<br>1 Sì<br>0 No<br>2 NA<br><br>Custom alignment: RH
76	[deficit_proteina_s]	Deficit di proteina S	radio<br>1 Sì<br>0 No<br>2 NA<br><br>Custom alignment: RH
77	[malattia_autoimmune]	Malattia autoimmune	radio<br>1 Sì<br>0 No<br><br>Custom alignment: RH
78	[quale_malattia_autoim m]<br><br>Show the field ONLY if: [malattia_autoimmune] = '1'	Quale	text
79	[mav_aneurisma]	MAV, aneurisma, angioma cavernoso	radio<br>1 Sì<br>0 No<br><br>Custom alignment: RH
80	[comorbidit_complete]	Section Header: *Form Status*<br>Complete?	dropdown<br>0 Incomplete<br>1 Unverified

			2 Complete
**Instrument: Familiarità (familiarit)**			
81	[malattia_fabry]	Malattia di Fabry	radio<br>1 Sì<br>0 No<br><br>Custom alignment: RH
82	[parentela_fabry]<br>Show the field ONLY if:<br>[malattia_fabry] = '1'	Grado di parentela	checkbox<br>1 parentela_fabry___1 Madre<br>2 parentela_fabry___2 Padre<br>3 parentela_fabry___3 Zio o zia o zii materni<br>4 parentela_fabry___4 Zio o zia o zii paterni<br>5 parentela_fabry___5 Nonna o nonno o nonni materni<br>6 parentela_fabry___6 Nonna o nonno o nonni paterni<br>7 parentela_fabry___7 fratello o sorella o fratelli
83	[ictus_ischemico]	Section Header:<br>Ictus ischemico	radio<br>1 Sì<br>0 No<br><br>Custom alignment: RH
84	[parentela_ictus_ische]<br>Show the field ONLY if:<br>[ictus_ischemico] = '1'	Grado di parentela	checkbox<br>1 parentela_ictus_ische___1 Madre<br>2 parentela_ictus_ische___2 Padre<br>3 parentela_ictus_ische___3 Zio o zia o zii materni<br>4 parentela_ictus_ische___4 Zio o zia o zii paterni<br>5 parentela_ictus_ische___5 Nonna o nonno o nonni materni<br>6 parentela_ictus_ische___6 Nonna o nonno o nonni paterni<br>7 parentela_ictus_ische___7 fratello o sorella o fratelli
85	[ictus_emorragico]	Section Header:<br>Ictus emorragico	radio<br>1 Sì<br>0 No<br><br>Custom alignment: RH
86	[parentela_ictus_emorr]<br>Show the field ONLY if:	Grado di parentela	checkbox<br>1 parentela_ictus_emorr___1 Madre

	[ictus_emorragico] = '1'		2 parentela_ictus_emorr___2 Padre<br>3 parentela_ictus_emorr___3 Zio o zia o zii materni<br>4 parentela_ictus_emorr___4 Zio o zia o zii paterni<br>5 parentela_ictus_emorr___5 Nonna o nonno o nonni materni<br>6 parentela_ictus_emorr___6 Nonna o nonno o nonni paterni<br>7 parentela_ictus_emorr___7 fratello o sorella o fratelli
87	[cefalea_emicrania]	Section Header:<br>Cefalea / emicrania con o senza aura	radio<br>1 Sì<br>0 No<br>Custom alignment: RH
88	[parentela_cefalea]<br>Show the field ONLY if:<br>[cefalea_emicrania] = '1'	Grado di parentela	checkbox<br>1 parentela_cefalea___1 Madre<br>2 parentela_cefalea___2 Padre<br>3 parentela_cefalea___3 Zio o zia o zii materni<br>4 parentela_cefalea___4 Zio o zia o zii paterni<br>5 parentela_cefalea___5 Nonna o nonno o nonni materni<br>6 parentela_cefalea___6 Nonna o nonno o nonni paterni<br>7 parentela_cefalea___7 fratello o sorella o fratelli
89	[decadimento_cognitivo]	Section Header:<br>Decadimento cognitivo	radio<br>1 Sì<br>0 No<br>Custom alignment: RH
90	[parentela_decadimento]<br>Show the field ONLY if:<br>[decadimento_cognitivo] = '1'	Grado di parentela	checkbox<br>1 parentela_decadimento___1 Madre<br>2 parentela_decadimento___2 Padre<br>3 parentela_decadimento___3 Zio o zia o zii materni<br>4 parentela_decadimento___4 Zio o zia o zii paterni

			5 parentela_decadimento___5 Nonna o nonno o nonni materni<br>6 parentela_decadimento___6 Nonna o nonno o nonni paterni<br>7 parentela_decadimento___7 fratello o sorella o fratelli
91	[disturbo_psichiatrico]	Section Header:<br>Disturbo psichiatrico	radio<br>1 Sì<br>0 No<br>Custom alignment: RH
92	[parentela_dist_psic]<br>Show the field ONLY if:<br>[disturbo_psichiatrico] = '1'	Grado di parentela	checkbox<br>1 parentela_dist_psic___1 Madre<br>2 parentela_dist_psic___2 Padre<br>3 parentela_dist_psic___3 Zio o zia o zii materni<br>4 parentela_dist_psic___4 Zio o zia o zii paterni<br>5 parentela_dist_psic___5 Nonna o nonno o nonni materni<br>6 parentela_dist_psic___6 Nonna o nonno o nonni paterni<br>7 parentela_dist_psic___7 fratello o sorella o fratelli
93	[epilessia]	Section Header:<br>Epilessia	radio<br>1 Sì<br>0 No<br>Custom alignment: RH
94	[parentela_epilessia]<br>Show the field ONLY if:<br>[epilessia] = '1'	Grado di parentela	checkbox<br>1 parentela_epilessia___1 Madre<br>2 parentela_epilessia___2 Padre<br>3 parentela_epilessia___3 Zio o zia o zii materni<br>4 parentela_epilessia___4 Zio o zia o zii paterni<br>5 parentela_epilessia___5 Nonna o nonno o nonni materni<br>6 parentela_epilessia___6 Nonna o nonno o nonni paterni

			7 | parentela_epilessia___7 | fratello o sorella o fratelli
95	[familiarit_complete]	Section Header: *Form Status*<br>Complete?	dropdown<br>0 | Incomplete<br>1 | Unverified<br>2 | Complete
**Instrument: Terapia Medica (terapia_medica)**			
96	[acido_acetilsalicilico]	Acido acetilsalicilico	radio<br>1 | Sì<br>0 | No<br>Custom alignment: RH
97	[specifica_acido_acetil]<br>Show the field ONLY if: [acido_acetilsalicilico] = '1'	Specifica	text
98	[clopidogrel]	Section Header:<br>Clopidogrel	radio<br>1 | Sì<br>0 | No<br>Custom alignment: RH
99	[altro_antiaggregante]	Section Header:<br>Altro antiaggregante	radio<br>1 | Sì<br>0 | No<br>Custom alignment: RH
100	[tipo_antiaggreg]<br>Show the field ONLY if: [altro_antiaggregante] = '1'	Tipo	text
101	[dose_antiaggreg]<br>Show the field ONLY if: [altro_antiaggregante] = '1'	Dose	text (number)
102	[antipertensivi]	Section Header:<br>Antipertensivi	radio<br>1 | Sì<br>0 | No<br>Custom alignment: RH
103	[tipo_antipertens]<br>Show the field ONLY if: [antipertensivi] = '1'	Tipo	text
104	[dose_antipertens]<br>Show the field ONLY if: [antipertensivi] = '1'	Dose	text (number)
105	[nao]	Section Header:<br>NAO	radio<br>1 | Sì

			0 No<br><br>Custom alignment: RH
106	[nome_nao]<br><br>Show the field ONLY if:<br>[nao] = '1'	Specificare il nome	text
107	[tao]	Section Header:<br>TAO	radio<br>1 Sì<br>0 No<br><br>Custom alignment: RH
108	[altro_anticoagulante]	Section Header:<br>Altro anticoagulante	radio<br>1 Sì<br>0 No<br><br>Custom alignment: RH
109	[statina]	Section Header:<br>Statina	radio<br>1 Sì<br>0 No<br><br>Custom alignment: RH
110	[tipo_statina]<br><br>Show the field ONLY if:<br>[statina] = '1'	Tipo	text
111	[dose_statina]<br><br>Show the field ONLY if:<br>[statina] = '1'	Dose	text (number)
112	[antiepilettico]	Section Header:<br>Antiepilettico	radio<br>1 Sì<br>0 No<br><br>Custom alignment: RH
113	[tipo_antipilettico]<br><br>Show the field ONLY if:<br>[antiepilettico] = '1'	Tipo	text
114	[dose_antipilettico]<br><br>Show the field ONLY if:<br>[antiepilettico] = '1'	Dose	text (number)
115	[ssri]	Section Header:<br>SSRI	radio<br>1 Sì<br>0 No<br><br>Custom alignment: RH
116	[tipo_ssri]<br><br>Show the field ONLY if:<br>[ssri] = '1'	Tipo	text
117	[dose_ssri]<br><br>Show the field ONLY if:<br>[ssri] = '1'	Dose	text

118	[snri]	Section Header: SNRI	radio<br>1 Sì<br>0 No<br>Custom alignment: RH
119	[tipo_snri]<br>Show the field ONLY if: [snri] = '1'	Tipo	text
120	[dose_snri]<br>Show the field ONLY if: [snri] = '1'	Dose	text (number)
121	[triptano]	Section Header: Triptano	radio<br>1 Sì<br>0 No<br>Custom alignment: RH
122	[tipo_triptano]<br>Show the field ONLY if: [triptano] = '1'	Tipo	text
123	[dose_triptano]<br>Show the field ONLY if: [triptano] = '1'	Dose	text (number)
124	[profilassi_cefalea_cron]	Section Header: Profilassi per cefalea cronica?	radio<br>1 Sì<br>0 No<br>Custom alignment: RH
125	[tipo_profilassi]<br>Show the field ONLY if: [profilassi_cefalea_cron] = '1'	Tipo	text
126	[dose_profilassi]<br>Show the field ONLY if: [profilassi_cefalea_cron] = '1'	Dose	text (number)
127	[terapia_chaperonica]	Section Header: Terapia chaperonica orale?	radio<br>1 Sì<br>0 No<br>Custom alignment: RH
128	[tipo_terapia_chaperonica]<br>Show the field ONLY if: [terapia_chaperonica] = '1'	Tipo	text
129	[dose_terapia_chaperonica]<br>Show the field ONLY if: [terapia_chaperonica] = '1'	Dose	text

130	[enzyme_replacement_tp]	Section Header:<br>Enzyme replacement therapy?	radio<br>1 Sì<br>0 No<br><br>Custom alignment: RH
131	[tipo_enzyme_repl_tp]<br><br>Show the field ONLY if:<br>[enzyme_replacement_tp] = '1'	Tipo	text
132	[dose_enzyme_repl_tp]<br><br>Show the field ONLY if:<br>[enzyme_replacement_tp] = '1'	Dose	text (number)
133	[terapia_medica_complete]	Section Header: *Form Status*<br>Complete?	dropdown<br>0 Incomplete<br>1 Unverified<br>2 Complete
**Instrument: Esami Diagnostico Strumentali (esami_diagnostico_strumentali)**			
134	[info]	Si intende l'ultima RMN encefalo disponibile al momento della valutazione	descriptive
135	[data_mri]	Data di esecuzione	text (date_dmy)
136	[tipo_rmn]	Tipo di RMN	radio<br>1 1 Tesla<br>2 1.5 Tesla<br>3 3 Tesla<br><br>Custom alignment: RH
137	[sequenze_rmn]	Sequenze presenti nella RMN	checkbox<br>1 sequenze_rmn___1 T2<br>2 sequenze_rmn___2 SWI<br>3 sequenze_rmn___3 FLAIR<br><br>Custom alignment: RH
138	[rmn_encefalo]	RMN encefalo	checkbox<br>1 rmn_encefalo___1 Emorragia lobare (l-ICH)<br>2 rmn_encefalo___2 Microemorragie lobari (CMBs)<br>3 rmn_encefalo___3 Iperintensità della sostanza bianca multifocali (White Matter Hyperintensities in a multispot pattern)<br>4 rmn_encefalo___4 Lesioni ischemiche situate lungo le zone di confine tra due territori vascolari (Watershed infarcts)

			5 rmn_encefalo___5 Spazi perivascolari del centro semiovale in numero > 20 (CSP-PVSs)<br>6 rmn_encefalo___6 Lacune ischemiche<br>7 rmn_encefalo___7 Restrizione in DWI
139	[coninvolgimento_monolat]	Il coinvolgimento vascolare è monolaterale?	radio<br>1 Sì<br>0 No<br>Custom alignment: RH
140	[specificare_coinvolg]<br>Show the field ONLY if: [coninvolgimento_monolat] = '1'	Specificare	text
141	[rachicentesi]	Section Header: *RACHICENTESI*<br>Rachicentesi	radio<br>1 Sì<br>0 No<br>Custom alignment: RH
142	[data_rachicentesi]<br>Show the field ONLY if: [rachicentesi] = '1'	Data di esecuzione	text (date_dmy)
143	[cellule]<br>Show the field ONLY if: [rachicentesi] = '1'	Cellule	text (number)
144	[proteine]<br>Show the field ONLY if: [rachicentesi] = '1'	Proteine	text (number)
145	[bande_oligoclonali]	Bande oligoclonali	radio<br>1 Sì<br>0 No<br>Custom alignment: RH
146	[tipo_bande_oligo]<br>Show the field ONLY if: [bande_oligoclonali] = '1'	Tipo	radio<br>1 Type 1 (no bands in CSF and serum)<br>2 Type 2 (oligoclonal IgG bands in CSF)<br>3 Type 3 (oligoclonal bands in CSF and serum with additional bands in CSF)<br>4 Type 4 (identical oligoclonal bands in CSF and serum)<br>5 Type 5 (monoclonal bands in CSF and serum)
147	[altro_rachi]	Altro	text
148	[anomalie_epilettiformi]	Section Header: *ALTRI ESAMI*<br>Sono evidenti anomalie epilettiformi all'EEG?	radio<br>1 Sì<br>0 No

			Custom alignment: RH
149	[specificare_anomalie]<br>Show the field ONLY if:<br>[anomalie_epilettiformi] = '1'	Specificare	text
150	[alterazioni_neuroftalm]	Alterazioni alla valutazione neuroftalmologica?	radio<br>1 Sì<br>0 No<br>Custom alignment: RH
151	[specificare_alterazioni]<br>Show the field ONLY if:<br>[alterazioni_neuroftalm] = '1'	Specificare	text
152	[altro_neuroftalm]<br>Show the field ONLY if:<br>[alterazioni_neuroftalm] = '1'	Altro	text
153	[alterazione_nefrologica]	Alterazioni alla valutazione nefrologica?	radio<br>1 Sì<br>0 No<br>Custom alignment: RH
154	[specificare_nefro]<br>Show the field ONLY if:<br>[alterazione_nefrologica] = '1'	Specificare	text
155	[altro_nefro]<br>Show the field ONLY if:<br>[alterazione_nefrologica] = '1'	Altro	text
156	[alterazioni_orl]	Alterazioni alla valutazione ORL?	radio<br>1 Sì<br>0 No<br>Custom alignment: RH
157	[specificare_orl]<br>Show the field ONLY if:<br>[alterazioni_orl] = '1'	Specificare	text
158	[alterazioni_snp]	Alterazioni del SNP/vegetativo?	radio<br>1 Sì<br>0 No<br>Custom alignment: RH
159	[specificare_snp]<br>Show the field ONLY if:<br>[alterazioni_snp] = '1'	Specificare	text
160	[alterazioni_cardio]	Alterazioni alla valutazione cardiologica?	radio

			1 Sì<br>0 No<br><br>Custom alignment: RH
161	[specificare_cardio]<br><br>Show the field ONLY if:<br>[alterazioni_cardio] = '1'	Specificare	text
162	[alterazioni_gastro]	Alterazioni alla valutazione gastrointestinale?	radio<br>1 Sì<br>0 No<br><br>Custom alignment: RH
163	[specificare_gastro]<br><br>Show the field ONLY if:<br>[alterazioni_gastro] = '1'	Specificare	text
164	[alteraz_biopsia_cutanea]	Alterazioni alla biopsia cutanea	radio<br>1 Sì<br>0 No<br><br>Custom alignment: RH
165	[specificare_biopsia]<br><br>Show the field ONLY if:<br>[alteraz_biopsia_cutanea] = '1'	Specificare	text
166	[alterazioni_alla_emg_eng]	Alterazioni alla EMG/ENG?	radio<br>1 Sì<br>0 No<br><br>Custom alignment: RH
167	[specificare_emg_eng]<br><br>Show the field ONLY if:<br>[alterazioni_alla_emg_eng] = '1'	Specificare	text
168	[esami_diagnostico_strumentali_complete]	Section Header: *Form Status*<br>Complete?	dropdown<br>0 Incomplete<br>1 Unverified<br>2 Complete
