# Supplementary material for: ALIGNED Network for rare cerebrovascular diseases: methodology and preliminary results
Source: Neurol Sci. 2026 Jun 22;47(7):584. doi: 10.1007/s10072-026-09183-1 (PMC13287270; doi:10.1007/s10072-026-09183-1)
Supplement: Supplementary file 4 — Supplementary file4 (PDF 949 KB) [file 10072_2026_9183_MOESM4_ESM.pdf]

Data Dictionary Codebook

04-10-2025 14:39

#	Variable / Field Name	Field Label *Field Note*	Field Attributes (Field Type, Validation, Choices, Calculations, etc.)
**Instrument: Anagrafica (anagrafica)**			
1	[record_id]	Record ID	text
2	[nome_ospedale]	Nome Ospedale	dropdown (autocomplete)<br>1 IRCCS ISNB UOC Neurologia e Rete Stroke- Ospedale Maggiore<br>2 Policlinico Universitario Campus Bio-medico di Roma<br>3 Ospedale di Pisa<br>4 Ospedale Apuane di Massa<br>5 IRCCS ISNB UOC Neuromet<br>6 Ospedale della Murgia, Altamura (Ba)<br>7 Ospedale San Francesco - ASL Nuoro<br>8 AUSL IRCCS di Reggio Emilia<br>9 Ospedale S. Eugenio ASL ROMA 2<br>10 Ospedale "Jazzolino" - Azienda Sanitaria Provinciale di Vibo Valentia<br>11 Fondazione Policlinico Universitario Agostino Gemelli, IRCCS, Roma<br>12 Udine University Hospital<br>13 AORN A. Cardarelli, Napoli<br>14 S.M. Goretti Hospital - Latina<br>15 Ospedale Vito Fazzi, Lecce<br>16 AOOR Villa Sofia- Cervello, Palermo<br>17 Ospedale Di Venere Bari<br>18 Ospedale Santa Maria delle Croci, Ravenna<br>19 IRCCS Neurolesi Bonino-Pulejo, Messina. U.O. Neurologia<br>20 Ospedale Santa Maria della Misericordia, Perugia<br>21 A.O. San Giovanni Addolorata - Roma<br>22 Ospedale Careggi -Firenze<br>23 Ospedale Dimiccoli - Barletta<br>24 Azienda Ospedaliera Universitaria di Modena<br>25 AOU G. Martino - Messina<br>26 SS Filippo & Nicola Hospital - Avezzano (L'Aquila)<br>27 ASST Papa Giovanni XXIII, Bergamo<br>28 IRCCS Humanitas Research Hospital, Rozzano<br>29 IRCCS Mondino, Pavia<br>30 ASST degli Spedali Civili, Brescia<br>31 ASST Ospedale Maggiore di Crema

			32 IRCCS Ospedale Policlinico San Martino di Genova<br>33 ASST Lariana<br>34 IRCCS Policlinico San Matteo, Pavia<br>35 Policlinico Tor Vergata, UOSD Stroke Unit<br>36 Ospedale Morgagni-Pierantoni, Forlì<br>37 Ospedale Bufalini, Cesena<br>38 PO Levante Asl 2 Savonese- Ospedale San Paolo Savona<br>39 ASST Rhodense<br>40 Ospedale Sant'Andrea, La Spezia<br>41 Fondazione IRCCS Ca' Granda Ospedale Maggiore Policlinico di Milano<br>42 Castrovillari ASP- Cosenza<br>43 Ospedale San Gerardo- Monza<br>44 Ospedale Sandro Pertini - ASL Roma2<br>45 Ospedale "Spaziani" di Frosinone<br>46 Ospedale di Pescara<br>47 Ospedale Luigi Sacco, ASST Fatebenefratelli Sacco<br>48 Fondazione IRCCS "Casa Sollievo della Sofferenza" - San Giovanni Rotondo (FG)<br>49 ASST Melegnano Martesana<br>50 Fondazione Istituto G. Giglio, Cefalù<br>51 ASST di Cremona<br>52 Ospedale Regionale Generale "F. Miulli", Acquaviva delle Fonti (BA)<br>53 ASST Grande Ospedale Metropolitano Niguarda<br>54 IRCCS Istituto Neurologico Carlo Besta
3	[spec_ospedale]<br>Show the field ONLY if:<br>[nome_ospedale] = '4'	Specificare	text
4	[mese_nascita]	Mese di nascita	dropdown<br>1 Gennaio<br>2 Febbraio<br>3 Marzo<br>4 Aprile<br>5 Maggio<br>6 Giugno<br>7 Luglio<br>8 Agosto<br>9 Settembre<br>10 Ottobre<br>11 Novembre<br>12 Dicembre
5	[anno_nascita]	Anno di nascita	text (integer, Min: 1924)
6	[data_visita]	Data visita<br>*Si intende la prima visita per sospetta MOYAMOYA presso il centro di riferimento e che ha implicato la raccolta dei dati (può quindi non coincidere con la data di compilazione del presente form)*	text (date_dmy)

7	[data_diagnosi]	Data di diagnosi<br>*Si intende la data in cui il paziente è stato sottoposto al primo studio neuroradiologico dei vasi intracranici; qualora non sia possibile datare con esattezza né il giorno né il mese della diagnosi, si utilizzi convenzionalmente la data 2 luglio.*	text (date_dmy)
8	[eta_reclutamento]	Età al reclutamento<br>*Si intende l'età al momento in cui il paziente è giunto all'attenzione del centro per accertamenti in merito a MOYAMOYA e si è provveduto a valutazione medica con raccolta dei dati inseriti in REDCap.*	text (integer)
9	[sesso]	Sesso	radio<br>1 F<br>2 M<br><br>Custom alignment: RH
10	[regione_nascita]	Regione di nascita	dropdown<br>1 Abruzzo<br>2 Basilicata<br>3 Calabria<br>4 Campania<br>5 Emilia Romagna<br>6 Friuli Venezia Giulia<br>7 Lazio<br>8 Liguria<br>9 Lombardia<br>10 Marche<br>11 Molise<br>12 Piemonte<br>13 Puglia<br>14 Sardegna<br>15 Sicilia<br>16 Toscana<br>17 Trentino Alto Adige<br>18 Umbria<br>19 Val d'Aosta<br>20 Veneto<br><br>Custom alignment: RH
11	[regione_residenza]	Regione di residenza	dropdown<br>1 Abruzzo<br>2 Basilicata<br>3 Calabria<br>4 Campania<br>5 Emilia Romagna<br>6 Friuli Venezia Giulia<br>7 Lazio<br>8 Liguria<br>9 Lombardia<br>10 Marche<br>11 Molise<br>12 Piemonte<br>13 Puglia<br>14 Sardegna

			15 Sicilia<br>16 Toscana<br>17 Trentino Alto Adige<br>18 Umbria<br>19 Val d'Aosta<br>20 Veneto<br><br>Custom alignment: RH
12	[etnia]	Gruppo etnico	dropdown<br>1 White<br>2 Black or African American<br>3 Asian<br>4 Other<br><br>Custom alignment: RH
13	[tipo_visita]	Tipo di visita	radio<br>1 Valutazione reparto<br>2 Controllo ambulatoriale
14	[scolarita]	Scolarità<br>*Numero di anni*	text (integer)
15	[anagrafica_complete]	Section Header: *Form Status*<br>Complete?	dropdown<br>0 Incomplete<br>1 Unverified<br>2 Complete
**Instrument: Genetica (genetica)**			
16	[studio_genetico]	Il paziente è stato sottoposto a studio genetico?	radio<br>1 Sì<br>0 No<br><br>Custom alignment: RH
17	[mutaz_rnf213]	È stata riscontrata una mutazione sul gene RNF213?	radio<br>1 Sì<br>0 No<br><br>Custom alignment: RH
18	[ulteriori_mutazioni]	Sono state rilevate ulteriori mutazioni?	radio<br>1 Sì<br>0 No<br><br>Custom alignment: RH
19	[specificare_geni]<br>Show the field ONLY if:<br>[ulteriori_mutazioni] = '1'	Specificare su quali geni	text
20	[genetica_complete]	Section Header: *Form Status*<br>Complete?	dropdown<br>0 Incomplete<br>1 Unverified<br>2 Complete
**Instrument: Evento Indice (evento_indice)**			
21	[data_evento]	Data evento Per uniformità di compilazione, con "evento indice" si è deciso di considerare la condizione medica che ha indotto all'esecuzione di studio neuroradiologico. Se quindi il paziente presenta cefalea	text (date_dmy)

		dal 2018,ma ha eseguito RMN encefalo/ AngioRMN/ AngioTC/AGF nel 2021 per un TIA, l'evento indice è da ritenersi il TIA e la data dell'evento il 2021. La cefalea verrà inserita nel paragrafo successivo "sintomi e segni associati". Qualora non sia possibile datare con esattezza il giorno dell'evento indice, si utilizzi convenzionalmente il giorno 15 del mese in cui l'evento sarebbe occorso; qualora non sia possibile datare con esattezza né il giorno né il mese dell'evento indice, si utilizzi convenzionalmente la data 2 luglio. Qualora il paziente fosse asintomatico, si prega lasciare vuoto questo campo.	
22	[evento_indice]	Tipo di evento indice	dropdown<br>1 TIA<br>2 Ictus ischemico<br>3 Emorragia cerebrale<br>4 Decadimento cognitivo<br>5 Depressione<br>6 Cefalea con o senza aura<br>7 Sintomi psichiatrici<br>8 Epilessia<br>9 Altro
23	[spec_evento_indice]<br>Show the field ONLY if:<br>[evento_indice] = '9'	Specificare	text
24	[nihss_acuta]	NIHSS in fase acuta	text
25	[mrs_prima]	Modified Rankin Scale (MRS) prima dell'evento indice	dropdown<br>0 0: Nessun sintomo<br>1 1: Nessuna inabilità significativa, pur manifestando sintomi: svolge ogni funzione e attività usuali<br>2 2: Leggera inabilità: incapace di svolgere tutte le attività precedenti, ma capace di occuparsi di sé senza assistenza<br>3 3: Inabilità moderata: richiede aiuto, ma cammina senza assistenza<br>4 4: Inabilità moderatamente severa: cammina con assistenza e necessità di assistenza per i propri bisogni corporei<br>5 5: Inabilità severa: allettamento, incontinenza, totalmente dipendente<br>6 6: Deceduto
26	[mrs_valutazione]	Modified Rankin Scale (MRS) alla valutazione	dropdown<br>0 0: Nessun sintomo<br>1 1: Nessuna inabilità significativa, pur manifestando sintomi: svolge ogni funzione e attività usuali<br>2 2: Leggera inabilità: incapace di svolgere tutte le attività precedenti, ma capace di occuparsi di sé senza assistenza<br>3 3: Inabilità moderata: richiede aiuto, ma cammina senza assistenza<br>4 4: Inabilità moderatamente severa: cammina con assistenza e necessità di assistenza per i propri bisogni corporei<br>5 5: Inabilità severa: allettamento, incontinenza, totalmente dipendente

			6 | 6: Deceduto
27	[eta_esordio]	Età all'esordio	text (integer)
28	[altri_eventi]	Altri EventiPer uniformità di compilazione, con "eventi precedenti" si è deciso di considerare esclusivamente gli eventi neurologici acuti, quali TIA, ictus ischemico, emorragia cerebrale, crisi epilettiche, occorsi prima dell'evento indice. Se ad esempio il paziente avesse avuto un TIA a gennaio 2021, quindi una emorragia cerebrale a maggio 2022, e si fosse giunti a RMN diagnostica a seguito di quest'ultima, "TIA" sarà da intendersi quale "evento precedente".	radio<br>1 | Sì<br>0 | No<br>2 | NA<br><br>Custom alignment: RH
29	[numero_altri_eventi]<br><br>Show the field ONLY if:<br>[altri_eventi] = '1'	Se sì, numero di altri eventi	radio<br>1 | 1<br>2 | 2<br>3 | 3<br>4 | Più di 3
30	[tipo_altri_eventi]<br><br>Show the field ONLY if:<br>[altri_eventi] = '1'	Tipo di altri eventi	checkbox<br>1 | tipo_altri_eventi___1 | TIA<br>2 | tipo_altri_eventi___2 | Ictus ischemico<br>3 | tipo_altri_eventi___3 | Emorragia cerebrale<br>4 | tipo_altri_eventi___4 | Crisi epilettica
31	[evento_indice_complete]	Section Header: *Form Status*<br>Complete?	dropdown<br>0 | Incomplete<br>1 | Unverified<br>2 | Complete
**Instrument: Sintomi e segni associati (sintomi_e_segni_associati)**			
32	[segni_associati]	Per uniformità di compilazione, si è deciso di comprendere quali "eventi e segni associati" le condizioni presenti nel corso della malattia.	radio<br>1 | Visita diretta<br>2 | Dedotto da cartella clinica
33	[cefalea]	Cefalea	radio<br>1 | Sì<br>0 | No<br><br>Custom alignment: RH
34	[anno_esordio_cefalea]<br><br>Show the field ONLY if:<br>[cefalea] = '1'	Anno di esordio	text (integer)
35	[tipo_cefalea]<br><br>Show the field ONLY if:<br>[cefalea] = '1'	Tipo di cefalea	radio<br>1 | Emicrania con aura<br>2 | Emicrania senz'aura<br>3 | Cefalea tensiva<br>4 | Altro tipo
36	[depressione]	Depressione	radio<br>1 | Sì<br>0 | No<br><br>Custom alignment: RH
37	[anno_esordio_depre]<br><br>Show the field ONLY if:<br>[depressione] = '1'	Anno di esordio	text (integer)
38	[quadro_cognitivo]	Quadro cognitivo	radio

			1 Cognitivamente integro<br>2 MCI<br>3 Decadimento cognitivo
39	[valutazioni_cogni]	Valutazioni	checkbox<br>1 valutazioni_cogni___1 MMSE<br>2 valutazioni_cogni___2 MOCA
40	[punteggio_mmse]<br>Show the field ONLY if:<br>[valutazioni_cogni(1)] = '1'	Punteggio MMSE<br>*da 0 a 30*	text (integer, Min: 0, Max: 30)<br>Field Annotation: @FORCE-MINMAX
41	[data_mmse]<br>Show the field ONLY if:<br>[valutazioni_cogni(1)] = '1'	Eseguito in data (MMSE)	text (date_dmy)
42	[punteggio_moca]<br>Show the field ONLY if:<br>[valutazioni_cogni(2)] = '1'	Punteggio MOCA<br>*da 0 a 30*	text (integer, Min: 0, Max: 30)<br>Field Annotation: @FORCE-MINMAX
43	[data_moca]<br>Show the field ONLY if:<br>[valutazioni_cogni(2)] = '1'	Eseguito in data (MOCA)	text (date_dmy)
44	[crisi_comiziale]	Crisi comiziale	radio<br>1 Sì<br>0 No<br>Custom alignment: RH
45	[tipo_crisi]<br>Show the field ONLY if:<br>[crisi_comiziale] = '1'	Tipo crisi	radio<br>1 Motoria<br>2 Non motoria
46	[specifica]<br>Show the field ONLY if:<br>[crisi_comiziale] = '1'	Specifica	radio<br>1 Onset focale<br>2 Onset generalizzato<br>3 Unknown onset
47	[anno_esordio_crisi]<br>Show the field ONLY if:<br>[crisi_comiziale] = '1'	Anno di esordio	text (integer, Min: 1924)
48	[sintomi_e_segni_associati_complete]	Section Header: *Form Status*<br>Complete?	dropdown<br>0 Incomplete<br>1 Unverified<br>2 Complete
**Instrument: Fattori di Rischio (fattori_di_rischio)**			
49	[fattori_di_rischio]	Fattori di rischio	checkbox<br>1 fattori_di_rischio___1 Ipertensione arteriosa<br>2 fattori_di_rischio___2 Diabete mellito<br>3 fattori_di_rischio___3 Dislipidemia<br>4 fattori_di_rischio___4 Cardiopatia ischemica (Attuale o pregresso: IM angina e/o trattamento endovascolare coronari<br>5 fattori_di_rischio___5 Fibrillazione atriale (Da considerarsi parossistic persistente o permaner presente anche se solo una singola occasione durante la vita del pazie

			6 | fattori_di_rischio___6 | Uso attuale di estroprogestinici (sostitutiva/anticoncezi<br>7 | fattori_di_rischio___7 | Abuso di sostanze
50	[ipertensione_arteriosa]	Ipertensione arteriosa	radio<br>1 | Sì<br>0 | No<br>Custom alignment: RH
51	[diabete_mellito]	Diabete mellito	radio<br>1 | Sì<br>0 | No<br>Custom alignment: RH
52	[dislipidemia]	Dislipidemia	radio<br>1 | Sì<br>0 | No<br>Custom alignment: RH
53	[cardiopatia_ischemica]	Cardiopatia ischemicaAttuale o pregresso: IMA e/o angina e/o trattamento endovascolare coronarico	radio<br>1 | Sì<br>0 | No<br>Custom alignment: RH
54	[fibrillazione_atriale]	Fibrillazione atrialeDa considerarsi parossistica, persistente o permanente e presente anche se solo in una singola occasione durante la vita del paziente	radio<br>1 | Sì<br>0 | No<br>Custom alignment: RH
55	[fumo]	Fumo di sigarettaPer uniformità di compilazione, si definisce "attivo" chi fuma regolarmente ogni giorno (anche una sola sigaretta) oppure ha smesso da meno di 12 mesi. Si considera "non fumatore" chi non ha mai fumato (ESC)	radio<br>1 | Sì, attivo<br>2 | Sì, pregresso<br>0 | No
56	[peso]	Peso<br>*kg*	text (number)
57	[altezza]	Altezza<br>*cm*	text (integer)
58	[bmi]	BMI	text (number)
59	[sedentarieta]	SedentarietàPer "persona sedentaria" si è deciso di utilizzare la definizione dell'Istituto Superiore di Sanità: "è una persona che non fa un lavoro pesante e che, nel tempo libero, non svolge attività fisica moderata o intensa".	radio<br>1 | Sì<br>0 | No<br>2 | NA<br>Custom alignment: RH
60	[estroprogestinici]	Uso attuale di estroprogestinici (sostitutiva/anticoncezionale)	radio<br>1 | Sì<br>0 | No<br>Custom alignment: RH
61	[alcol]	Abuso di alcol (> 3 unità / die per ♂ e > 2 unità / die per ♀)	radio<br>1 | Sì, attuale<br>2 | Sì, pregresso<br>0 | No

62	[iperomocisteinemia]	Iperomocisteinemia (omocisteina ≥ 15 micromol/L)	radio<br>1 Sì<br>0 No<br>2 NA<br>Custom alignment: RH
63	[abuso_sostanze]	Abuso di sostanze	radio<br>1 Sì<br>0 No<br>Custom alignment: RH
64	[riscontro_pfo]	Riscontro di forame ovale pervio (PFO)?Per forame ovale pervio si intende il riscontro di highintensity transient signals allo studio doppler transcranicocon bubble test. Si intenda per grado "lieve" (1-10 microbolle),"moderato" (11-30 microbolle), "severo" (>30 microbolle) inbasale o dopo manovra di Valsalva.	radio<br>0 No<br>1 Si lieve<br>2 Si moderato<br>3 Si severo
65	[fattori_di_rischio_complete]	Section Header: *Form Status*<br>Complete?	dropdown<br>0 Incomplete<br>1 Unverified<br>2 Complete
**Instrument: Comorbidità (comorbidit)**			
66	[deficit_coagulazione]	Deficit di coagulazione	radio<br>1 Sì<br>0 No<br>2 NA<br>Custom alignment: RH
67	[deficit_proteina_c]	Deficit di proteina C	radio<br>1 Sì<br>0 No<br>2 NA<br>Custom alignment: RH
68	[deficit_proteina_s]	Deficit di proteina S	radio<br>1 Sì<br>0 No<br>2 NA<br>Custom alignment: RH
69	[malattia_autoimmune]	Malattia autoimmune	radio<br>1 Sì<br>0 No<br>Custom alignment: RH
70	[quale_malattia_autoimm]<br>Show the field ONLY if: [malattia_autoimmune] = '1'	Quale	text
71	[mav_aneurisma]	MAV, aneurisma, angioma cavernoso	radio<br>1 Sì<br>0 No

			Custom alignment: RH
72	[sindrome_di_down]	Sindrome di Down	radio<br>1 Sì<br>0 No<br>2 NA<br>Custom alignment: RH
73	[radio_cerebrale]	Pregressa radioterapia cerebrale	radio<br>1 Sì<br>0 No<br>2 NA<br>Custom alignment: RH
74	[neurofibromatosi_1]	Neurofibromatosi tipo I	radio<br>1 Sì<br>0 No<br>2 NA<br>Custom alignment: RH
75	[anamnesi_neopl_cerebr]	Anamnesi positiva per neoplasia cerebrale	radio<br>1 Sì<br>0 No<br>Custom alignment: RH
76	[pregressa_meningite]	Pregressa meningite	radio<br>1 Sì<br>0 No<br>Custom alignment: RH
77	[anemia_falciforme]	Anemia falciforme	radio<br>1 Sì<br>0 No<br>Custom alignment: RH
78	[altre_comorbidita]	Altro	text
79	[sindrome_moyamoya]	Diagnosi di Sindrome di Moyamoya (o Quasi-moyamoya)?	radio<br>1 Sì<br>0 No<br>Custom alignment: RH
80	[comorbidit_complete]	Section Header: *Form Status*<br>Complete?	dropdown<br>0 Incomplete<br>1 Unverified<br>2 Complete
**Instrument: Familiarità (familiarit)**			
81	[arteriopatia_moyamoya]	Arteriopatia Moyamoya	radio<br>1 Sì<br>0 No<br>Custom alignment: RH
82	[parentela_moyamoya]<br>Show the field ONLY if:	Grado di parentela	checkbox<br>1 parentela_moyamoya___1 Madre

	[arteriopatia_moyamoya] = '1'		2 parentela_moyamoya___2 Padre<br>3 parentela_moyamoya___3 Zio o zia o zii materni<br>4 parentela_moyamoya___4 Zio o zia o zii paterni<br>5 parentela_moyamoya___5 Nonna o nonno o nonni materni<br>6 parentela_moyamoya___6 Nonna o nonno o nonni paterni<br>7 parentela_moyamoya___7 fratello o sorella o fratelli
83	[ictus_ischemico]	Section Header:<br>Ictus ischemico	radio<br>1 Sì<br>0 No<br>Custom alignment: RH
84	[parentela_ictus_ische]<br>Show the field ONLY if: [ictus_ischemico] = '1'	Grado di parentela	checkbox<br>1 parentela_ictus_ische___1 Madre<br>2 parentela_ictus_ische___2 Padre<br>3 parentela_ictus_ische___3 Zio o zia o zii materni<br>4 parentela_ictus_ische___4 Zio o zia o zii paterni<br>5 parentela_ictus_ische___5 Nonna o nonno o nonni materni<br>6 parentela_ictus_ische___6 Nonna o nonno o nonni paterni<br>7 parentela_ictus_ische___7 fratello o sorella o fratelli
85	[ictus_emorragico]	Section Header:<br>Ictus emorragico	radio<br>1 Sì<br>0 No<br>Custom alignment: RH
86	[parentela_ictus_emorr]<br>Show the field ONLY if: [ictus_emorragico] = '1'	Grado di parentela	checkbox<br>1 parentela_ictus_emorr___1 Madre<br>2 parentela_ictus_emorr___2 Padre<br>3 parentela_ictus_emorr___3 Zio o zia o zii materni<br>4 parentela_ictus_emorr___4 Zio o zia o zii paterni<br>5 parentela_ictus_emorr___5 Nonna o nonno o nonni materni<br>6 parentela_ictus_emorr___6 Nonna o nonno o nonni paterni<br>7 parentela_ictus_emorr___7 fratello o sorella o fratelli

87	[cefalea_emicrania]	Section Header:<br>Cefalea / emicrania con o senza aura	radio<br>1 Sì<br>0 No<br><br>Custom alignment: RH
88	[parentela_cefalea]<br>Show the field ONLY if:<br>[cefalea_emicrania] = '1'	Grado di parentela	checkbox<br>1 parentela_cefalea___1 Madre<br>2 parentela_cefalea___2 Padre<br>3 parentela_cefalea___3 Zio o zia o zii materni<br>4 parentela_cefalea___4 Zio o zia o zii paterni<br>5 parentela_cefalea___5 Nonna o nonno o nonni materni<br>6 parentela_cefalea___6 Nonna o nonno o nonni paterni<br>7 parentela_cefalea___7 fratello o sorella o fratelli
89	[decadimento_cognitivo]	Section Header:<br>Decadimento cognitivo	radio<br>1 Sì<br>0 No<br><br>Custom alignment: RH
90	[parentela_decadimento]<br>Show the field ONLY if:<br>[decadimento_cognitivo] = '1'	Grado di parentela	checkbox<br>1 parentela_decadimento___1 Madre<br>2 parentela_decadimento___2 Padre<br>3 parentela_decadimento___3 Zio o zia o zii materni<br>4 parentela_decadimento___4 Zio o zia o zii paterni<br>5 parentela_decadimento___5 Nonna o nonno o nonni materni<br>6 parentela_decadimento___6 Nonna o nonno o nonni paterni<br>7 parentela_decadimento___7 fratello o sorella o fratelli
91	[disturbo_psichiatrico]	Section Header:<br>Disturbo psichiatrico	radio<br>1 Sì<br>0 No<br><br>Custom alignment: RH
92	[parentela_dist_psic]<br>Show the field ONLY if:<br>[disturbo_psichiatrico] = '1'	Grado di parentela	checkbox<br>1 parentela_dist_psic___1 Madre<br>2 parentela_dist_psic___2 Padre<br>3 parentela_dist_psic___3 Zio o zia o zii materni<br>4 parentela_dist_psic___4 Zio o zia o zii paterni<br>5 parentela_dist_psic___5 Nonna o nonno o nonni materni

			6 | parentela_dist_psic___6 | Nonna o nonno o nonni paterni<br>7 | parentela_dist_psic___7 | fratello o sorella o fratelli
93	[epilessia]	Section Header:<br>Epilessia	radio<br>1 | Sì<br>0 | No<br>Custom alignment: RH
94	[parentela_epilessia]<br>Show the field ONLY if:<br>[epilessia] = '1'	Grado di parentela	checkbox<br>1 | parentela_epilessia___1 | Madre<br>2 | parentela_epilessia___2 | Padre<br>3 | parentela_epilessia___3 | Zio o zia o zii materni<br>4 | parentela_epilessia___4 | Zio o zia o zii paterni<br>5 | parentela_epilessia___5 | Nonna o nonno o nonni materni<br>6 | parentela_epilessia___6 | Nonna o nonno o nonni paterni<br>7 | parentela_epilessia___7 | fratello o sorella o fratelli
95	[familiarit_complete]	Section Header: *Form Status*<br>Complete?	dropdown<br>0 | Incomplete<br>1 | Unverified<br>2 | Complete
**Instrument: Terapia (terapia)**			
96	[acido_acetilsalicilico]	Acido acetilsalicilico	radio<br>1 | Sì<br>0 | No<br>Custom alignment: RH
97	[specifica_acido_acetil]<br>Show the field ONLY if:<br>[acido_acetilsalicilico] = '1'	Specifica	text
98	[clopidogrel]	Section Header:<br>Clopidogrel	radio<br>1 | Sì<br>0 | No<br>Custom alignment: RH
99	[altro_antiaggregante]	Section Header:<br>Altro antiaggregante	radio<br>1 | Sì<br>0 | No<br>Custom alignment: RH
100	[tipo_antiaggreg]<br>Show the field ONLY if:<br>[altro_antiaggregante] = '1'	Tipo	text
101	[dose_antiaggreg]<br>Show the field ONLY if:<br>[altro_antiaggregante] = '1'	Dose	text (number)

102	[antipertensivi]	Section Header:<br>Antipertensivi	radio<br>1 Sì<br>0 No<br><br>Custom alignment: RH
103	[tipo_antipertens]<br><br>Show the field ONLY if:<br>[antipertensivi] = '1'	Tipo	text
104	[dose_antipertens]<br><br>Show the field ONLY if:<br>[antipertensivi] = '1'	Dose	text (number)
105	[nao]	Section Header:<br>NAO	radio<br>1 Sì<br>0 No<br><br>Custom alignment: RH
106	[nome_nao]<br><br>Show the field ONLY if:<br>[nao] = '1'	Specificare il nome	text
107	[tao]	Section Header:<br>TAO	radio<br>1 Sì<br>0 No<br><br>Custom alignment: RH
108	[altro_anticoagulante]	Section Header:<br>Altro anticoagulante	radio<br>1 Sì<br>0 No<br><br>Custom alignment: RH
109	[statina]	Section Header:<br>Statina	radio<br>1 Sì<br>0 No<br><br>Custom alignment: RH
110	[tipo_statina]<br><br>Show the field ONLY if:<br>[statina] = '1'	Tipo	text
111	[dose_statina]<br><br>Show the field ONLY if:<br>[statina] = '1'	Dose	text (number)
112	[antiepilettico]	Section Header:<br>Antiepilettico	radio<br>1 Sì<br>0 No<br><br>Custom alignment: RH
113	[tipo_antipilettico]<br><br>Show the field ONLY if:<br>[antiepilettico] = '1'	Tipo	text
114	[dose_antipilettico]<br><br>Show the field ONLY if:<br>[antiepilettico] = '1'	Dose	text (number)
115	[ssri]	Section Header:<br>SSRI	radio<br>1 Sì

			0 No<br><br>Custom alignment: RH
116	[tipo_ssri]<br><br>Show the field ONLY if:<br>[ssri] = '1'	Tipo	text
117	[dose_ssri]<br><br>Show the field ONLY if:<br>[ssri] = '1'	Dose	text (number)
118	[snri]	Section Header:<br>SNRI	radio<br>1 Sì<br>0 No<br><br>Custom alignment: RH
119	[tipo_snri]<br><br>Show the field ONLY if:<br>[snri] = '1'	Tipo	text
120	[dose_snri]<br><br>Show the field ONLY if:<br>[snri] = '1'	Dose	text (number)
121	[triptano]	Section Header:<br>Triptano	radio<br>1 Sì<br>0 No<br><br>Custom alignment: RH
122	[tipo_triptano]<br><br>Show the field ONLY if:<br>[triptano] = '1'	Tipo	text
123	[dose_triptano]<br><br>Show the field ONLY if:<br>[triptano] = '1'	Dose	text (number)
124	[profilassi_cefalea_cron]	Section Header:<br>Profilassi per cefalea cronica?	radio<br>1 Sì<br>0 No<br><br>Custom alignment: RH
125	[tipo_profilassi]<br><br>Show the field ONLY if:<br>[profilassi_cefalea_cron] = '1'	Tipo	text
126	[dose_profilassi]<br><br>Show the field ONLY if:<br>[profilassi_cefalea_cron] = '1'	Dose	text (number)
127	[tratt_rivascol_sin]	Section Header: *Trattamento chirurgico*<br>Trattamento di rivascolarizzazione dell'emisfero sinistro?	radio<br>1 Sì<br>0 No<br><br>Custom alignment: RH
128	[tipo_rivascol_sin]<br><br>Show the field ONLY if:<br>[tratt_rivascol_sin] = '1'	Tipo di rivascolarizzazione	radio<br>1 Diretta<br>2 Indiretta<br><br>Custom alignment: RH

129	[spec_rivasc_ind_sin]<br><br>Show the field ONLY if:<br>[tipo_rivascol_sin] = '2'	Specificare	checkbox<br>1 spec_rivasc_ind_sin___1 Encefalomiosinangio<br>2 spec_rivasc_ind_sin___2 Encefaloduroarterios<br>3 spec_rivasc_ind_sin___3 Encefaloduroarterior
130	[data_rivasc_sin]<br><br>Show the field ONLY if:<br>[tratt_rivascol_sin] = '1'	Data di esecuzione	text (date_dmy)
131	[tratt_rivascol_destro]	Section Header:<br>Trattamento di rivascolarizzazione dell'emisfero destro?	radio<br>1 Sì<br>0 No<br><br>Custom alignment: RH
132	[tipo_rivascol_destro]<br><br>Show the field ONLY if:<br>[tratt_rivascol_destro] = '1'	Tipo di rivascolarizzazione	radio<br>1 Diretta<br>2 Indiretta<br><br>Custom alignment: RH
133	[spec_rivasc_ind_destro]<br><br>Show the field ONLY if:<br>[tipo_rivascol_destro] = '2'	Specificare	checkbox<br>1 spec_rivasc_ind_destro___1 Encefalomiosinar<br>2 spec_rivasc_ind_destro___2 Encefaloduroarte<br>3 spec_rivasc_ind_destro___3 Encefaloduroarte
134	[data_rivasc_destro]<br><br>Show the field ONLY if:<br>[tratt_rivascol_destro] = '1'	Data di esecuzione	text (date_dmy)
135	[terapia_complete]	Section Header: *Form Status*<br>Complete?	dropdown<br>0 Incomplete<br>1 Unverified<br>2 Complete
**Instrument: Esami Diagnostico Strumentali (esami_diagnostico_strumentali)**			
136	[info]	Si intende l'ultima RMN encefalo disponibile al momento della valutazione	descriptive
137	[data_mri]	Data di esecuzione	text (date_dmy)
138	[tipo_rmn]	Tipo di RMN	radio<br>1 1 Tesla<br>2 1.5 Tesla<br>3 3 Tesla<br><br>Custom alignment: RH
139	[sequenze_rmn]	Sequenze presenti nella RMN	checkbox<br>1 sequenze_rmn___1 T2<br>2 sequenze_rmn___2 SWI<br>3 sequenze_rmn___3 FLAIR<br><br>Custom alignment: RH
140	[rmn_encefalo]	RMN encefalo	checkbox<br>1 rmn_encefalo___1 Emorragia lobare (I-ICH)<br>2 rmn_encefalo___2 Microemorragie lobari (CMBs)<br>3 rmn_encefalo___3 Iperintenistà della sostanza bianca multifocali (White

			Matter Hyperintensities in a multispot pattern)<br>4 rmn_encefalo___4 Lesioni ischemiche situate lungo le zone di confine tra due territori vascolari (Watershed infarcts)<br>5 rmn_encefalo___5 Spazi perivascolari del centro semiovale in numero > 20 (CSP-PVSs)<br>6 rmn_encefalo___6 Lacune ischemiche<br>7 rmn_encefalo___7 Restrizione in DWI
141	[coninvolgimento_monolat]	Il coinvolgimento vascolare è monolaterale?	radio<br>1 Sì<br>0 No<br><br>Custom alignment: RH
142	[specificare_coinvolg]<br><br>Show the field ONLY if:<br>[coninvolgimento_monolat] = '1'	Specificare	text
143	[riscontri]	Riscontri allo studio angiografico della RM (MRA) o all'angio-TC (CTA)	radio<br>1 ICA<br>2 MCA<br>3 ACA<br>4 PCA<br>5 Ivy-sign (segno dell'edera)<br>6 Small abnormal net-like vessels
144	[specifica_ica]<br><br>Show the field ONLY if:<br>[riscontri] = '1'	Specifica ICA	checkbox<br>1 specifica_ica___1 Normal<br>2 specifica_ica___2 Stenosis of C1<br>3 specifica_ica___3 Discontinuity of C1 signal<br>4 specifica_ica___4 Invisible
145	[specifica_mca]<br><br>Show the field ONLY if:<br>[riscontri] = '2'	Specifica MCA	checkbox<br>1 specifica_mca___1 Normal<br>2 specifica_mca___2 Stenosis of M1<br>3 specifica_mca___3 Discontinuity of M1 signal<br>4 specifica_mca___4 Invisible
146	[specifica_aca]<br><br>Show the field ONLY if:<br>[riscontri] = '3'	Specifica ACA	checkbox<br>1 specifica_aca___1 Normal A2 and its distal<br>2 specifica_aca___2 A2 and its distal signal decrease or loss<br>3 specifica_aca___3 Invisible
147	[specifica_pca]<br><br>Show the field ONLY if:<br>[riscontri] = '4'	Specifica PCA	checkbox<br>1 specifica_pca___1 Normal P2 and its distal<br>2 specifica_pca___2 P2 and its distal signal decrease or loss

			3 specifica_pca___3 Invisible
148	[rachicentesi]	Section Header: *RACHICENTESI*<br>Rachicentesi	radio<br>1 Sì<br>0 No<br>Custom alignment: RH
149	[data_rachicentesi]<br>Show the field ONLY if:<br>[rachicentesi] = '1'	Data di esecuzione	text (date_dmy)
150	[cellule]<br>Show the field ONLY if:<br>[rachicentesi] = '1'	Cellule	text (number)
151	[proteine]<br>Show the field ONLY if:<br>[rachicentesi] = '1'	Proteine	text (number)
152	[bande_oligoclonali]	Bande oligoclonali	radio<br>1 Sì<br>0 No<br>Custom alignment: RH
153	[tipo_bande_oligo]<br>Show the field ONLY if:<br>[bande_oligoclonali] = '1'	Tipo	radio<br>1 Type 1 (no bands in CSF and serum)<br>2 Type 2 (oligoclonal IgG bands in CSF)<br>3 Type 3 (oligoclonal bands in CSF and serum with additional bands in CSF)<br>4 Type 4 (identical oligoclonal bands in CSF and serum)<br>5 Type 5 (monoclonal bands in CSF and serum)
154	[altro_rachi]	Altro	text
155	[anomalie_epilettiformi]	Section Header: *ALTRI ESAMI*<br>Sono evidenti anomalie epilettiformi all'EEG?	radio<br>1 Sì<br>0 No<br>Custom alignment: RH
156	[specificare_anomalie]<br>Show the field ONLY if:<br>[anomalie_epilettiformi] = '1'	Specificare	text
157	[alterazioni_neuroftalm]	Alterazioni alla valutazione neuroftalmologica?	radio<br>1 Sì<br>0 No<br>Custom alignment: RH
158	[specificare_alterazioni]<br>Show the field ONLY if:<br>[alterazioni_neuroftalm] = '1'	Specificare	text
159	[altro_neuroftalm]<br>Show the field ONLY if:<br>[alterazioni_neuroftalm] = '1'	Altro	text

160	[data_dsa]	Section Header: *DIGITAL SUBTRACTION ANGIOGRAPHY (DSA) CEREBRALE*<br>Data di esecuzione	text (date_dmy)
161	[coinvolgimento_monolat]	Il coinvolgimento vascolare è monolaterale?	radio<br>1 Sì<br>0 No<br>Custom alignment: RH
162	[spec_coinvolg]<br>Show the field ONLY if:<br>[coinvolgimento_monolat] = '1'	Specificare	text
163	[suzuki_staging]	Stadio al Suzuki staging system:	checkbox<br>1 suzuki_staging___1 1. Narrowing of the carotid fork (i.e., ICA bifurcation)<br>2 suzuki_staging___2 2. Initiation of the moyamoya: continued narrowing of the ICA; dilation of the ACA and MCA; initial moyamoya blush<br>3 suzuki_staging___3 3. Intensification of the moyamoya: loss of proximal ACA and MCA; leptomeningeal collateralization from the PCA; increase in moyamoya blush<br>4 suzuki_staging___4 4. Minimization of the moyamoya: progressive occlusion of ICA reaching origin of PCA; reduction in moyamoya blush<br>5 suzuki_staging___5 5. Reduction of the moyamoya: complete loss of ICA, ACA, and MCA; increased collateral supply from ECA; further reduction in moyamoya blush<br>6 suzuki_staging___6 6. Disappearance of the moyamoya: disappearance of blood supply from ICA; blood supply exclusively from ECA; disappearance of moyamoya vessels<br>Custom alignment: LV
164	[esami_diagnostico_strumentali_complete]	Section Header: *Form Status*<br>Complete?	dropdown<br>0 Incomplete<br>1 Unverified<br>2 Complete
**Instrument: PET Cerebrale (pet_cerebrale)**			
165	[pet_cerebrale]	PET Cerebrale	radio<br>1 Sì

			0 No<br><br>Custom alignment: RH
166	[stimolo_acetazolamide]	Dopo stimolo con Acetazolamide?	radio<br>1 Sì<br>0 No<br><br>Custom alignment: RH
167	[data_pet]	Data di esecuzione	text (date_dmy)
168	[pet_cerebrale_complete]	Section Header: *Form Status*<br>Complete?	dropdown<br>0 Incomplete<br>1 Unverified<br>2 Complete
