# Supplementary material for: ALIGNED Network for rare cerebrovascular diseases: methodology and preliminary results
Source: Neurol Sci. 2026 Jun 22;47(7):584. doi: 10.1007/s10072-026-09183-1 (PMC13287270; doi:10.1007/s10072-026-09183-1)
Supplement: Supplementary file 5 — Supplementary file5 (PDF 848 KB) [file 10072_2026_9183_MOESM5_ESM.pdf]

Data Dictionary Codebook

04-10-2025 14:39

#	Variable / Field Name	Field Label<br>*Field Note*	Field Attributes (Field Type, Validation, Choices, Calculations, etc.)
**Instrument: Anagrafica (anagrafica)**			
1	[record_id]	Record ID	text
2	[nome_ospedale]	Nome Ospedale	dropdown<br>1 IRCCS ISNB UOC Neurologia e Rete Stroke- Ospedale Maggiore<br>2 Policlinico Universitario Campus Bio-medico di Roma<br>3 Ospedale di Pisa<br>4 Ospedale Apuane di Massa<br>5 IRCCS ISNB UOC Neuromet<br>6 Ospedale della Murgia, Altamura (Ba)<br>7 Ospedale San Francesco - ASL Nuoro<br>8 AUSL IRCCS di Reggio Emilia<br>9 Ospedale S. Eugenio ASL ROMA 2<br>10 Ospedale "Jazzolino" - Azienda Sanitaria Provinciale di Vibo Valentia<br>11 Fondazione Policlinico Universitario Agostino Gemelli, IRCCS, Roma<br>12 Udine University Hospital<br>13 AORN A. Cardarelli, Napoli<br>14 S.M. Goretti Hospital - Latina<br>15 Ospedale Vito Fazzi, Lecce<br>16 AOOR Villa Sofia- Cervello, Palermo<br>17 Ospedale Di Venere Bari<br>18 Ospedale Santa Maria delle Croci, Ravenna<br>19 IRCCS Neurolesi Bonino-Pulejo, Messina. U.O. Neurologia<br>20 Ospedale Santa Maria della Misericordia, Perugia<br>21 A.O. San Giovanni Addolorata - Roma<br>22 Ospedale Careggi -Firenze<br>23 Ospedale Dimiccoli - Barletta<br>24 Azienda Ospedaliera Universitaria di Modena<br>25 AOU G. Martino - Messina<br>26 SS Filippo & Nicola Hospital - Avezzano (L'Aquila)<br>27 ASST Papa Giovanni XXIII, Bergamo<br>28 IRCCS Humanitas Research Hospital, Rozzano

			29 IRCCS Mondino, Pavia<br>30 ASST degli Spedali Civili, Brescia<br>31 ASST Ospedale Maggiore di Crema<br>32 IRCCS Ospedale Policlinico San Martino di Genova<br>33 ASST Lariana<br>34 IRCCS Policlinico San Matteo, Pavia<br>35 Policlinico Tor Vergata, UOSD Stroke Unit<br>36 Ospedale Morgagni-Pierantoni, Forlì<br>37 Ospedale Bufalini, Cesena<br>38 PO Levante Asl 2 Savonese- Ospedale San Paolo Savona<br>39 ASST Rhodense<br>40 Ospedale Sant'Andrea, La Spezia<br>41 Fondazione IRCCS Ca' Granda Ospedale Maggiore Policlinico di Milano<br>42 Castrovillari ASP- Cosenza<br>43 Ospedale San Gerardo- Monza<br>44 Ospedale Sandro Pertini - ASL Roma2<br>45 Ospedale "Spaziani" di Frosinone<br>46 Ospedale di Pescara<br>47 Ospedale Luigi Sacco, ASST Fatebenefratelli Sacco<br>48 Fondazione IRCCS "Casa Sollievo della Sofferenza" - San Giovanni Rotondo (FG)<br>49 ASST Melegnano Martesana<br>50 Fondazione Istituto G. Giglio, Cefalù<br>51 ASST di Cremona<br>52 Ospedale Regionale Generale "F. Miulli", Acquaviva delle Fonti (BA)<br>53 ASST Grande Ospedale Metropolitano Niguarda<br>54 IRCCS Istituto Neurologico Carlo Besta
3	[spec_ospedale]<br>Show the field ONLY if:<br>[nome_ospedale] = '4'	Specificare	text
4	[mese_nascita]	Mese di nascita	dropdown<br>1 Gennaio<br>2 Febbraio<br>3 Marzo<br>4 Aprile<br>5 Maggio<br>6 Giugno<br>7 Luglio

			8 Agosto<br>9 Settembre<br>10 Ottobre<br>11 Novembre<br>12 Dicembre
5	[anno_nascita]	Anno di nascita	text (integer, Min: 1924)
6	[data_visita]	Data visita<br>*Si intende la prima visita per sospetta sindrome di Sneddon presso il centro di riferimento e che ha implicato la raccolta dei dati (può quindi non coincidere con la data di compilazione del presente form)*	text (date_dmy)
7	[data_diagnosi]	Data di diagnosi<br>*Qualora non sia possibile datare con esattezza né il giorno né il mese della diagnosi, si utilizzi convenzionalmente la data 2 luglio dell'anno della diagnosi.*	text (date_dmy)
8	[eta_reclutamento]	Età al reclutamento<br>*Si intende l'età al momento in cui il paziente è giunto all'attenzione del centro per accertamenti in merito a sindrome di Sneddon e si è provveduto a valutazione medica con raccolta dei dati inseriti in REDCap.Si intende l'età al momento in cui il paziente è giunto all'attenzione del centro per accertamenti in merito a sindrome di Sneddon e si è provveduto a valutazione medica con raccolta dei dati inseriti in REDCap.*	text (integer)
9	[sesso]	Sesso	radio<br>1 F<br>2 M<br><br>Custom alignment: RH
10	[regione_nascita]	Regione di nascita	dropdown<br>1 Abruzzo<br>2 Basilicata<br>3 Calabria<br>4 Campania<br>5 Emilia Romagna<br>6 Friuli Venezia Giulia<br>7 Lazio<br>8 Liguria<br>9 Lombardia<br>10 Marche<br>11 Molise<br>12 Piemonte<br>13 Puglia<br>14 Sardegna<br>15 Sicilia<br>16 Toscana<br>17 Trentino Alto Adige<br>18 Umbria<br>19 Val d'Aosta<br>20 Veneto<br><br>Custom alignment: RH

11	[regione_residenza]	Regione di residenza	dropdown<br>1 Abruzzo<br>2 Basilicata<br>3 Calabria<br>4 Campania<br>5 Emilia Romagna<br>6 Friuli Venezia Giulia<br>7 Lazio<br>8 Liguria<br>9 Lombardia<br>10 Marche<br>11 Molise<br>12 Piemonte<br>13 Puglia<br>14 Sardegna<br>15 Sicilia<br>16 Toscana<br>17 Trentino Alto Adige<br>18 Umbria<br>19 Val d'Aosta<br>20 Veneto<br><br>Custom alignment: RH
12	[etnia]	Gruppo etnico	dropdown<br>1 White<br>2 Black or African American<br>3 Asian<br>4 Other<br><br>Custom alignment: RH
13	[tipo_visita]	Tipo di visita	radio<br>1 Valutazione reparto<br>2 Controllo ambulatoriale
14	[scolarita]	Scolarità<br>*Numero di anni*	text (integer)
15	[anagrafica_complete]	Section Header: *Form Status*<br>Complete?	dropdown<br>0 Incomplete<br>1 Unverified<br>2 Complete
Instrument: **Evento Indice** (evento_indice)			
16	[data_evento]	Data evento Per uniformità di compilazione, con "evento indice" si è deciso di considerare la condizione medica che ha indotto all'esecuzione di RMN encefalo/ AngioRMN/ AngioTC/AGF. Se quindi il paziente presenta livedo reticularis dal 2018, ma ha eseguito RMN encefalo nel 2021 per un TIA, l'evento indice è da ritenersi il TIA e la data dell'evento il 2021. La livedo reticularis verrà	text (date_dmy)

		inserita nel paragrafo successivo "sintomi e segni associati". Qualora non sia possibile datare con esattezza il giorno dell'evento indice, si utilizzi convenzionalmente il giorno 15 del mese in cui l'evento sarebbe occorso; qualora non sia possibile datare con esattezza né il giorno né il mese dell'evento indice, si utilizzi convenzionalmente la data 2 luglio.	
17	[evento_indice]	Tipo di evento indice neurologico	dropdown<br>1 TIA<br>2 Ictus ischemico<br>3 Emorragia cerebrale<br>4 Decadimento cognitivo<br>5 Epilessia<br>6 Cefalea<br>7 Altro
18	[spec_evento_indice]<br>Show the field ONLY if:<br>[evento_indice] = '7'	Specificare	text
19	[nihss_acuta]	NIHSS in fase acuta	text
20	[mrs_prima]	Modified Rankin Scale (MRS) prima dell'evento indice	dropdown<br>0 0: Nessun sintomo<br>1 1: Nessuna inabilità significativa, pur manifestando sintomi: svolge ogni funzione e attività usuali<br>2 2: Leggera inabilità: incapace di svolgere tutte le attività precedenti, ma capace di occuparsi di sé senza assistenza<br>3 3: Inabilità moderata: richiede aiuto, ma cammina senza assistenza<br>4 4: Inabilità moderatamente severa: cammina con assistenza e necessità di assistenza per i propri bisogni corporei<br>5 5: Inabilità severa: allettamento, incontinenza, totalmente dipendente<br>6 6: Deceduto
21	[mrs_valutazione]	Modified Rankin Scale (MRS) alla valutazione	dropdown<br>0 0: Nessun sintomo<br>1 1: Nessuna inabilità significativa, pur manifestando sintomi: svolge ogni funzione e attività usuali<br>2 2: Leggera inabilità: incapace di svolgere tutte le attività precedenti, ma capace di occuparsi di sé senza assistenza<br>3 3: Inabilità moderata: richiede aiuto, ma cammina senza assistenza<br>4 4: Inabilità moderatamente severa: cammina con assistenza e necessità di assistenza per i propri bisogni corporei

			5 — 5: Inabilità severa: allettamento, incontinenza, totalmente dipendente<br>6 — 6: Deceduto
22	[eta_esordio]	Età all'esordio	text (integer)
23	[altri_eventi]	Altri EventiPer uniformità di compilazione, con "altri eventi" si è deciso di considerare esclusivamente gli eventi neurologici acuti, quali TIA, ictus ischemico, emorragia cerebrale, occorsi prima, durante o dopol'evento indice. Se ad esempio il paziente avesse avuto un TIA a gennaio 2021, quindi una emorragia cerebrale a maggio 2022, e si fosse giunti a RMN diagnostica a seguito di quest'ultima, "TIA" sarà da intendersi quale "altro evento".	radio<br>1 — Sì<br>0 — No<br>2 — NA
24	[numero_altri_eventi]<br>Show the field ONLY if: [altri_eventi] = '1'	Se sì, numero di altri eventi	radio<br>1 — 1<br>2 — 2<br>3 — 3<br>4 — Più di 3
25	[tipo_altri_eventi]<br>Show the field ONLY if: [altri_eventi] = '1'	Tipo di altri eventi	checkbox<br>1 — tipo_altri_eventi___1 — TIA<br>2 — tipo_altri_eventi___2 — Ictus ischemico<br>3 — tipo_altri_eventi___3 — Emorragia cerebrale<br>4 — tipo_altri_eventi___4 — Crisi epilettica
26	[evento_indice_complete]	Section Header: *Form Status*<br>Complete?	dropdown<br>0 — Incomplete<br>1 — Unverified<br>2 — Complete
**Instrument: Sintomi e segni associati (sintomi_e_segni_associati)**			
27	[segni_associati]	Per uniformità di compilazione, si è deciso di comprendere quali "eventi e segni associati" le condizioni presenti nel corso della malattia.	radio<br>1 — Visita diretta<br>2 — Dedotto da cartella clinica
28	[cefalea]	Cefalea	radio<br>1 — Sì<br>0 — No<br>Custom alignment: RH
29	[anno_esordio_cefalea]<br>Show the field ONLY if: [cefalea] = '1'	Anno di esordio	text (integer)
30	[tipo_cefalea]<br>Show the field ONLY if: [cefalea] = '1'	Tipo di cefalea	radio<br>1 — Emicrania con aura<br>2 — Emicrania senz'aura<br>3 — Cefalea tensiva<br>4 — Altro tipo
31	[depressione]	Depressione	radio<br>1 — Sì

			0 No<br><br>Custom alignment: RH
32	[anno_esordio_depre]<br><br>Show the field ONLY if:<br>[depressione] = '1'	Anno di esordio	text (integer)
33	[quadro_cognitivo]	Quadro cognitivo	radio<br>1 Cognitivamente integro<br>2 MCI<br>3 Decadimento cognitivo
34	[valutazioni_cogni]	Valutazioni	checkbox<br>1 valutazioni_cogni___1 MMSE<br>2 valutazioni_cogni___2 MOCA
35	[punteggio_mmse]<br><br>Show the field ONLY if:<br>[valutazioni_cogni(1)] = '1'	Punteggio MMSE<br>*da 0 a 30*	text (integer, Min: 0, Max: 30)<br>Field Annotation: @FORCE-MINMAX
36	[data_mmse]<br><br>Show the field ONLY if:<br>[valutazioni_cogni(1)] = '1'	Eseguito in data (MMSE)	text (date_dmy)
37	[punteggio_moca]<br><br>Show the field ONLY if:<br>[valutazioni_cogni(2)] = '1'	Punteggio MOCA<br>*da 0 a 30*	text (integer, Min: 0, Max: 30)<br>Field Annotation: @FORCE-MINMAX
38	[data_moca]<br><br>Show the field ONLY if:<br>[valutazioni_cogni(2)] = '1'	Eseguito in data (MOCA)	text (date_dmy)
39	[aborto_spontaneo]	Aborto spontaneo	radio<br>1 Sì<br>0 No<br><br>Custom alignment: RH
40	[sindr_anti_fosfolipidi]	Sindrome da anticorpi anti fosfolipidi	radio<br>1 Sì<br>0 No<br><br>Custom alignment: RH
41	[livedo_reticularis]	Livedo Reticularis / Racemosa	radio<br>1 Arti superiori<br>2 Arti inferiori<br>3 Volto<br>4 Tronco (addome, torace, dorso)
42	[vasoreattivita_cerebrale]	Alterata vasoreattività cerebrale allo studio doppler transcranico?Per alterata vasoreattività cerebrale si intende il riscontro di valori di breath holding index (BHI) al doppler transcranico inferiori a 0.69	radio<br>1 Si bilateralmente<br>2 Si monolateralmente<br>0 No

43	[sintomi_e_segni_associati_complete]	Section Header: *Form Status*<br>Complete?	dropdown<br>0 Incomplete<br>1 Unverified<br>2 Complete
**Instrument: Fattori di Rischio (fattori_di_rischio)**			
44	[fattori_di_rischio]	Fattori di rischio	checkbox<br>1 fattori_di_rischio___1 Ipertensione arteric<br>2 fattori_di_rischio___2 Diabete mellito<br>3 fattori_di_rischio___3 Dislipidemia<br>4 fattori_di_rischio___4 Cardiopatia ischemi (Attuale o pregressc angina e/o trattame endovascolare coro<br>5 fattori_di_rischio___5 Fibrillazione atriale considerarsi paross persistente o perma presente anche se s una singola occasio durante la vita del p<br>6 fattori_di_rischio___6 Uso attuale di estroprogestinici (sostitutiva/anticon<br>7 fattori_di_rischio___7 Abuso di sostanze
45	[ipertensione_arteriosa]	Ipertensione arteriosa	radio<br>1 Sì<br>0 No<br>Custom alignment: RH
46	[diabete_mellito]	Diabete mellito	radio<br>1 Sì<br>0 No<br>Custom alignment: RH
47	[dislipidemia]	Dislipidemia	radio<br>1 Sì<br>0 No<br>Custom alignment: RH
48	[cardiopatia_ischemica]	Cardiopatia ischemicaAttuale o pregresso: IMA e/o angina e/o trattamento endovascolare coronarico	radio<br>1 Sì<br>0 No<br>Custom alignment: RH
49	[fibrillazione_atriale]	Fibrillazione atrialeDa considerarsi parossistica, persistente o permanente e presente anche se solo in una singola occasione durante la vita del paziente	radio<br>1 Sì<br>0 No<br>Custom alignment: RH
50	[fumo]	Fumo di sigarettaPer uniformità di compilazione, si definisce "attivo" chi fuma regolarmente ogni giorno (anche una sola sigaretta) oppure ha	radio<br>1 Sì, attivo

		smesso da meno di 12 mesi. Si considera "non fumatore" chi non ha mai fumato (ESC)	2 Sì, pregresso<br>0 No
51	[peso]	Peso<br>*kg*	text (number)
52	[altezza]	Altezza<br>*cm*	text (integer)
53	[bmi]	BMI	text (number)
54	[sedentarieta]	SedentarietàPer "persona sedentaria" si è deciso di utilizzare la definizione dell'Istituto Superiore di Sanità: "è una persona che non fa un lavoro pesante e che, nel tempo libero, non svolge attività fisica moderata o intensa".	radio<br>1 Sì<br>0 No<br>2 NA<br>Custom alignment: RH
55	[estroprogestinici]	Uso attuale di estroprogestinici (sostitutiva/anticoncezionale)	radio<br>1 Sì<br>0 No<br>Custom alignment: RH
56	[alcol]	Abuso di alcol (> 3 unità / die per ♂ e > 2 unità / die per ♀)	radio<br>1 Sì, attuale<br>2 Sì, pregresso<br>0 No
57	[iperomocisteinemia]	Iperomocisteinemia (omocisteina ≥ 15 micromol/L)	radio<br>1 Sì<br>0 No<br>2 NA<br>Custom alignment: RH
58	[abuso_sostanze]	Abuso di sostanze	radio<br>1 Sì<br>0 No<br>Custom alignment: RH
59	[riscontro_pfo]	Riscontro di forame ovale pervio (PFO)?Per forame ovale pervio si intende il riscontro di highintensity transient signals allo studio doppler transcranicocon bubble test. Si intenda per grado "lieve" (1-10 microbolle),"moderato" (11-30 microbolle), "severo" (>30 microbolle) inbasale o dopo manovra di Valsalva.	radio<br>0 No<br>1 Si lieve<br>2 Si moderato<br>3 Si severo
60	[fattori_di_rischio_complete]	Section Header: *Form Status*<br>Complete?	dropdown<br>0 Incomplete<br>1 Unverified<br>2 Complete
**Instrument: Comorbidità (comorbidit)**			
61	[deficit_coagulazione]	Deficit di coagulazione	radio<br>1 Sì<br>0 No<br>2 NA

			Custom alignment: RH
62	[deficit_proteina_c]	Deficit di proteina C	radio<br>1 Sì<br>0 No<br>2 NA<br>Custom alignment: RH
63	[deficit_proteina_s]	Deficit di proteina S	radio<br>1 Sì<br>0 No<br>2 NA<br>Custom alignment: RH
64	[malattia_autoimmune]	Malattia autoimmune	radio<br>1 Sì<br>0 No<br>Custom alignment: RH
65	[quale_malattia_autoimm]<br>Show the field ONLY if:<br>[malattia_autoimmune] = '1'	Quale	text
66	[mav_aneurisma]	MAV, aneurisma, angioma cavernoso	radio<br>1 Sì<br>0 No<br>Custom alignment: RH
67	[comorbidit_complete]	Section Header: *Form Status*<br>Complete?	dropdown<br>0 Incomplete<br>1 Unverified<br>2 Complete
**Instrument: Familiarità (familiarit)**			
68	[sneddon]	Sindrome di Sneddon	radio<br>1 Sì<br>0 No<br>Custom alignment: RH
69	[parentela_sneddon]<br>Show the field ONLY if:<br>[sneddon] = '1'	Grado di parentela	checkbox<br>1 parentela_sneddon___1 Madre<br>2 parentela_sneddon___2 Padre<br>3 parentela_sneddon___3 Zio o zia o zii materni<br>4 parentela_sneddon___4 Zio o zia o zii paterni<br>5 parentela_sneddon___5 Nonna o nonno o nonni materni

			6 parentela_sneddon___6 Nonna o nonno o nonni paterni<br>7 parentela_sneddon___7 fratello o sorella o fratelli
70	[ictus_ischemico]	Ictus ischemico	radio<br>1 Sì<br>0 No<br><br>Custom alignment: RH
71	[parentela_ictus_ische]<br><br>Show the field ONLY if:<br>[ictus_ischemico] = '1'	Grado di parentela	checkbox<br>1 parentela_ictus_ische___1 Madre<br>2 parentela_ictus_ische___2 Padre<br>3 parentela_ictus_ische___3 Zio o zia o zii materni<br>4 parentela_ictus_ische___4 Zio o zia o zii paterni<br>5 parentela_ictus_ische___5 Nonna o nonno o nonni materni<br>6 parentela_ictus_ische___6 Nonna o nonno o nonni paterni<br>7 parentela_ictus_ische___7 fratello o sorella o fratelli
72	[ictus_emorragico]	Ictus emorragico	radio<br>1 Sì<br>0 No<br><br>Custom alignment: RH
73	[parentela_ictus_emorr]<br><br>Show the field ONLY if:<br>[ictus_emorragico] = '1'	Grado di parentela	checkbox<br>1 parentela_ictus_emorr___1 Madre<br>2 parentela_ictus_emorr___2 Padre<br>3 parentela_ictus_emorr___3 Zio o zia o zii materni<br>4 parentela_ictus_emorr___4 Zio o zia o zii paterni<br>5 parentela_ictus_emorr___5 Nonna o nonno o nonni materni<br>6 parentela_ictus_emorr___6 Nonna o nonno o nonni paterni

			7 | parentela_ictus_emorr___7 | fratello o sorella o fratelli
74	[cefalea_emicrania]	Cefalea / emicrania con o senza aura	radio<br>1 | Sì<br>0 | No<br><br>Custom alignment: RH
75	[parentela_cefalea]<br>Show the field ONLY if:<br>[cefalea_emicrania] = '1'	Grado di parentela	checkbox<br>1 | parentela_cefalea___1 | Madre<br>2 | parentela_cefalea___2 | Padre<br>3 | parentela_cefalea___3 | Zio o zia o zii materni<br>4 | parentela_cefalea___4 | Zio o zia o zii paterni<br>5 | parentela_cefalea___5 | Nonna o nonno o nonni materni<br>6 | parentela_cefalea___6 | Nonna o nonno o nonni paterni<br>7 | parentela_cefalea___7 | fratello o sorella o fratelli
76	[decadimento_cognitivo]	Decadimento cognitivo	radio<br>1 | Sì<br>0 | No<br><br>Custom alignment: RH
77	[parentela_decadimento]<br>Show the field ONLY if:<br>[decadimento_cognitivo] = '1'	Grado di parentela	checkbox<br>1 | parentela_decadimento___1 | Madre<br>2 | parentela_decadimento___2 | Padre<br>3 | parentela_decadimento___3 | Zio o zia o zii materni<br>4 | parentela_decadimento___4 | Zio o zia o zii paterni<br>5 | parentela_decadimento___5 | Nonna o nonno o nonni materni<br>6 | parentela_decadimento___6 | Nonna o nonno o nonni paterni<br>7 | parentela_decadimento___7 | fratello o sorella o fratelli
78	[disturbo_psichiatrico]	Disturbo psichiatrico	radio<br>1 | Sì<br>0 | No

			Custom alignment: RH
79	[parentela_dist_psic]<br>Show the field ONLY if: [disturbo_psichiatrico] = '1'	Grado di parentela	checkbox<br>1 parentela_dist_psic___1 Madre<br>2 parentela_dist_psic___2 Padre<br>3 parentela_dist_psic___3 Zio o zia o zii materni<br>4 parentela_dist_psic___4 Zio o zia o zii paterni<br>5 parentela_dist_psic___5 Nonna o nonno o nonni materni<br>6 parentela_dist_psic___6 Nonna o nonno o nonni paterni<br>7 parentela_dist_psic___7 fratello o sorella o fratelli
80	[epilessia]	Epilessia	radio<br>1 Sì<br>0 No<br>Custom alignment: RH
81	[parentela_epilessia]<br>Show the field ONLY if: [epilessia] = '1'	Grado di parentela	checkbox<br>1 parentela_epilessia___1 Madre<br>2 parentela_epilessia___2 Padre<br>3 parentela_epilessia___3 Zio o zia o zii materni<br>4 parentela_epilessia___4 Zio o zia o zii paterni<br>5 parentela_epilessia___5 Nonna o nonno o nonni materni<br>6 parentela_epilessia___6 Nonna o nonno o nonni paterni<br>7 parentela_epilessia___7 fratello o sorella o fratelli
82	[familiarit_complete]	Section Header: *Form Status*<br>Complete?	dropdown<br>0 Incomplete<br>1 Unverified<br>2 Complete
**Instrument: Terapia Medica (terapia_medica)**			
83	[acido_acetilsalicilico]	Acido acetilsalicilico	radio<br>1 Sì<br>0 No<br>Custom alignment: RH

84	[specifica_acido_acetil]<br>Show the field ONLY if: [acido_acetilsalicilico] = '1'	Specificare	text
85	[clopidogrel]	Clopidogrel	radio<br>1 Sì<br>0 No<br>Custom alignment: RH
86	[altro_antiaggregante]	Altro antiaggregante	radio<br>1 Sì<br>0 No<br>Custom alignment: RH
87	[tipo_dose_antiaggreg]<br>Show the field ONLY if: [altro_antiaggregante] = '1'	Tipo e dose	text
88	[antipertensivi]	Antipertensivi	radio<br>1 Sì<br>0 No<br>Custom alignment: RH
89	[tipo_dose_antipertens]<br>Show the field ONLY if: [antipertensivi] = '1'	Tipo e dose	text
90	[nao]	NAO	radio<br>1 Sì<br>0 No<br>Custom alignment: RH
91	[tao]	TAO	radio<br>1 Sì<br>0 No<br>Custom alignment: RH
92	[altro_anticoagulante]	Altro anticoagulante	radio<br>1 Sì<br>0 No<br>Custom alignment: RH
93	[statina]	Statina	radio<br>1 Sì<br>0 No<br>Custom alignment: RH
94	[tipo_dose_statina]<br>Show the field ONLY if:	Tipo e dose	text

	[statina] = '1'		
95	[antiepilettico]	Antiepilettico	radio<br>1 Sì<br>0 No<br><br>Custom alignment: RH
96	[tipo_dose_antipiletti co]<br><br>Show the field ONLY if:<br>[antiepilettico] = '1'	Tipo e dose	text
97	[ssri]	SSRI	radio<br>1 Sì<br>0 No<br><br>Custom alignment: RH
98	[tipo_dose_ssri]<br><br>Show the field ONLY if:<br>[ssri] = '1'	Tipo e dose	text
99	[snri]	SNRI	radio<br>1 Sì<br>0 No<br><br>Custom alignment: RH
100	[tipo_dose_snri]<br><br>Show the field ONLY if:<br>[snri] = '1'	Tipo e dose	text
101	[triptano]	Triptano	radio<br>1 Sì<br>0 No<br><br>Custom alignment: RH
102	[tipo_dose_triptano]<br><br>Show the field ONLY if:<br>[triptano] = '1'	Tipo e dose	text
103	[profilassi_cefalea_cr on]	Profilassi per cefalea cronica?	radio<br>1 Sì<br>0 No<br><br>Custom alignment: RH
104	[tipo_profilassi]<br><br>Show the field ONLY if:<br>[profilassi_cefalea_cron] = '1'	Tipo	text
105	[dose_profilassi]<br><br>Show the field ONLY if:<br>[profilassi_cefalea_cron] = '1'	Dose	text (number)
106	[terapia_medica_comple te]	Section Header: *Form Status*<br>Complete?	dropdown<br>0 Incomplete<br>1 Unverified

			2 Complete
**Instrument: Esami Diagnostico Strumentali (esami_diagnostico_strumentali)**			
107	[info]	Si intende l'ultima RMN encefalo disponibile al momento della valutazione	descriptive
108	[data_mri]	Data di esecuzione	text (date_dmy)
109	[tipo_rmn]	Tipo di RMN	radio<br>1 | 1 Tesla<br>2 | 1.5 Tesla<br>3 | 3 Tesla<br>Custom alignment: RH
110	[sequenze_rmn]	Sequenze presenti nella RMN	checkbox<br>1 | sequenze_rmn___1 | T2<br>2 | sequenze_rmn___2 | SWI<br>3 | sequenze_rmn___3 | FLAIR<br>Custom alignment: RH
111	[rmn_encefalo]	RMN encefalo	checkbox<br>1 | rmn_encefalo___1 | Emorragia lobare (l-ICH)<br>2 | rmn_encefalo___2 | Microemorragie lobari (CMBs)<br>3 | rmn_encefalo___3 | Iperintenistà della sostanza bianca multifocali (White Matter Hyperintensities in a multispot pattern)<br>4 | rmn_encefalo___4 | Siderosi corticale superficiale (CSS)<br>5 | rmn_encefalo___5 | Spazi perivascolari del centro semiovale in numero > 20 (CSP-PVSs)<br>6 | rmn_encefalo___6 | Lacune ischemiche<br>7 | rmn_encefalo___7 | Restrizione in DWI
112	[rmn_libera_emorr]	La RMN è libera da emorragie in sede profonda?	radio<br>1 | Sì<br>0 | No<br>Custom alignment: RH
113	[svd_score]	SVD score	radio<br>0 | 0<br>1 | 1<br>2 | 2<br>3 | 3<br>4 | 4
114	[dwi]	DWI	radio

			1 Focale<br>2 Disseminata
115	[riscontri]	Riscontri allo studio angiografico della RM (MRA) o all'angio-TC (CTA)	radio<br>1 ICA<br>2 MCA<br>3 ACA<br>4 PCA<br>5 Ivy-sign (segno dell'edera)<br>6 Small abnormal net-like vessels
116	[ica]<br>Show the field ONLY if:<br>[riscontri] = '1'	ICA	radio<br>1 Normal<br>2 Stenosis of C1<br>3 Discontinuity of C1 signal<br>4 Invisible
117	[mca]<br>Show the field ONLY if:<br>[riscontri] = '2'	MCA	radio<br>1 Normal<br>2 Stenosis of M1<br>3 Discontinuity of M1 signal<br>4 Invisible
118	[aca]<br>Show the field ONLY if:<br>[riscontri] = '3'	ACA	radio<br>1 Normal A2 and its distal<br>2 A2 and its distal signal decrease or loss<br>3 Invisible
119	[pca]<br>Show the field ONLY if:<br>[riscontri] = '4'	PCA	radio<br>1 Normal P2 and its distal<br>2 P2 and its distal signal decrease or loss<br>3 Invisible
120	[rachicentesi]	Section Header: *RACHICENTESI*<br>Rachicentesi	radio<br>1 Sì<br>0 No<br>Custom alignment: RH
121	[r_data_di_esecuzione]<br>Show the field ONLY if:<br>[rachicentesi] = '1'	Data di esecuzione	text (date_dmy)<br>Custom alignment: RH
122	[cellule]<br>Show the field ONLY if:<br>[rachicentesi] = '1'	Cellule	text (number)<br>Custom alignment: RH
123	[proteine]<br>Show the field ONLY if:<br>[rachicentesi] = '1'	Proteine	text (number)<br>Custom alignment: RH
124	[bande_oligoclonali]	Bande oligoclonali	radio<br>1 Sì

			0 No<br><br>Custom alignment: RH
125	[tipo]<br><br>Show the field ONLY if: [bande_oligoclonali] = '1'	Tipo	radio<br>1 Type 1 (no bands in CSF and serum)<br>2 Type 2 (oligoclonal IgG bands in CSF)<br>3 Type 3 (oligoclonal bands in CSF and serum with additional bands in CSF)<br>4 Type 4 (identical oligoclonal bands in CSF and serum)<br>5 Type 5 (monoclonal bands in CSF and serum)
126	[altro]	Altro	text
127	[anomalie_epilettiformi_eeg]	Section Header: *ALTRI ESAMI*<br><br>Sono evidenti anomalie epilettiformi all'EEG	radio<br>1 Sì<br>0 No<br><br>Custom alignment: RH
128	[specificare_anomalie]<br><br>Show the field ONLY if: [anomalie_epilettiformi_eeg] = '1'	Specificare	text<br>Custom alignment: RH
129	[alteraz_valutaz_neuroftalm]	Alterazioni alla valutazione neuroftalmologica?	radio<br>1 Sì<br>0 No<br><br>Custom alignment: RH
130	[specificare_neuroftalm]<br><br>Show the field ONLY if: [alteraz_valutaz_neuroftalm] = '1'	Specificare	text<br>Custom alignment: RH
131	[alterazioni_trombo_occlusi]	Alterazioni trombo-occlusive alla biopsia di cute?	radio<br>1 Sì<br>0 No<br><br>Custom alignment: RH
132	[specificare_alt_tromb_occl]<br><br>Show the field ONLY if: [alterazioni_trombo_occlusi] = '1'	Specificare	text<br>Custom alignment: RH
133	[altri_esami_altro]	Altro	text<br>Custom alignment: RH
134	[esami_diagnostico_strumentali_complete]	Section Header: *Form Status*<br><br>Complete?	dropdown<br>0 Incomplete<br>1 Unverified<br>2 Complete

Instrument: **Angiografia Encefalo** (angiografia_encefalo)

135	[info2]	Si intende l'ultima angiografia encefalo disponibile al momento della valutazione	descriptive
136	[data_angiografia]	Data di esecuzione	text (date_dmy)
137	[angiografia_encefalo]	Angiografia encefalo	checkbox<br>1 angiografia_encefalo___1 Stenosi<br>2 angiografia_encefalo___2 Occlusione M1<br>3 angiografia_encefalo___3 Network di vasi collaterali
138	[specifica_stenosi]<br>Show the field ONLY if: [angiografia_encefalo(1)] = '1'	Specifica Stenosi	checkbox<br>1 specifica_stenosi___1 M1<br>2 specifica_stenosi___2 M2<br>3 specifica_stenosi___3 M3<br>4 specifica_stenosi___4 ACA<br>5 specifica_stenosi___5 ACP<br>6 specifica_stenosi___6 ACoA<br>7 specifica_stenosi___7 ACoP<br>8 specifica_stenosi___8 BA<br>9 specifica_stenosi___9 V<br>10 specifica_stenosi___10 SCA<br>11 specifica_stenosi___11 AICA<br>12 specifica_stenosi___12 PICA
139	[specifica_stenosi_m1]<br>Show the field ONLY if: [specifica_stenosi(1)] = '1'	Specifica Stenosi M1	radio<br>1 Dx<br>2 Sx<br>Custom alignment: RH
140	[specifica_occlusione]<br>Show the field ONLY if: [angiografia_encefalo(2)] = '1'	Specifica Occlusione	checkbox<br>1 specifica_occlusione___1 M1<br>2 specifica_occlusione___2 M2<br>3 specifica_occlusione___3 M3<br>4 specifica_occlusione___4 ACA<br>5 specifica_occlusione___5 ACP<br>6 specifica_occlusione___6 ACoA<br>7 specifica_occlusione___7 ACoP<br>8 specifica_occlusione___8 BA<br>9 specifica_occlusione___9 V<br>10 specifica_occlusione___10 SCA<br>11 specifica_occlusione___11 AICA<br>12 specifica_occlusione___12 PICA
141	[angiografia_encefalo_complete]	Section Header: *Form Status*<br>Complete?	dropdown<br>0 Incomplete<br>1 Unverified<br>2 Complete
