# Supplementary material for: ALIGNED Network for rare cerebrovascular diseases: methodology and preliminary results
Source: Neurol Sci. 2026 Jun 22;47(7):584. doi: 10.1007/s10072-026-09183-1 (PMC13287270; doi:10.1007/s10072-026-09183-1)
Supplement: Supplementary file 6 — Supplementary file6 (PDF 65.2 KB) [file 10072_2026_9183_MOESM6_ESM.pdf]

Survey ALIGNED

Please complete the survey below.

Thank you!

Ospedale

- ◯ Ospedale Maggiore IRCCS Ist Scienze Neurologiche di Bologna
- ◯ Policlinico Universitario Campus Bio-medico di Roma
- ◯ Ospedale di Pisa
- ◯ Ospedale Apuane di Massa
- ◯ UOS Neurologia-AOU-Clinica Neurologica rete Neurologica Metropolitana-Neuro-Met IRCCS Istituto delle Scienze Neurologiche di Bologna Italia
- ◯ Ospedale della Murgia Altamura (Ba)
- ◯ Ospedale San Francesco - ASL Nuoro
- ◯ AUSL IRCCS di Reggio Emilia
- ◯ Ospedale S. Eugenio ASL ROMA 2
- ◯ Ospedale "Jazzolino" - Azienda Sanitaria Provinciale di Vibo Valentia
- ◯ Fondazione Policlinico Universitario Agostino Gemelli IRCCS Roma
- ◯ Udine University Hospital
- ◯ AORN A. Cardarelli Napoli
- ◯ S.M. Goretti Hospital - Latina
- ◯ Ospedale Vito Fazzi Lecce
- ◯ AOOR Villa Sofia- Cervello Palermo
- ◯ Ospedale Di Venere Bari
- ◯ Ospedale Santa Maria delle Croci Ravenna
- ◯ IRCCS Neurolesi Bonino-Pulejo Messina. U.O. Neurologia
- ◯ Ospedale Santa Maria della Misericordia Perugia
- ◯ A.O. San Giovanni Addolorata - Roma
- ◯ Ospedale Careggi - Firenze
- ◯ Ospedale Dimiccoli - Barletta
- ◯ Azienda Ospedaliera Universitaria di Modena
- ◯ AOU G. Martino - Messina
- ◯ SS Filippo & Nicola Hospital - Avezzano (L'Aquila)
- ◯ ASST Papa Giovanni XXIII Bergamo
- ◯ IRCCS Humanitas Research Hospital Rozzano
- ◯ IRCCS Mondino Pavia
- ◯ ASST degli Spedali Civili Brescia
- ◯ ASST Ospedale Maggiore di Crema
- ◯ IRCCS Ospedale Policlinico San Martino di Genova
- ◯ ASST Lariana
- ◯ IRCCS Policlinico San Matteo Pavia
- ◯ Policlinico Tor Vergata UOSD Stroke Unit
- ◯ Ospedale Morgagni-Pierantoni Forlì
- ◯ Ospedale Bufalini Cesena
- ◯ PO Levante Asl 2 Savonese- Ospedale San Paolo Savona
- ◯ ASST Rhodense
- ◯ Ospedale Sant'Andrea La Spezia
- ◯ Fondazione IRCCS Ca' Granda Ospedale Maggiore Policlinico di Milano
- ◯ Castrovillari ASP - Cosenza
- ◯ Ospedale San Gerardo - Monza
- ◯ Ospedale Sandro Pertini - ASL Roma2
- ◯ Ospedale "Spaziani" di Frosinone
- ◯ Ospedale di Pescara
- ◯ Ospedale Luigi Sacco ASST Fatebenefratelli Sacco
- ◯ Fondazione IRCCS "Casa Sollievo della Sofferenza" - San Giovanni Rotondo (FG)
- ◯ ASST Melegnano Martesana
- ◯ Fondazione Istituto G. Giglio Cefalù
- ◯ ASST di Cremona
- ◯ Ospedale Regionale Generale "F. Miulli" Acquaviva delle Fonti (BA)
- ◯ ASST Grande Ospedale Metropolitano Niguarda
- ◯ IRCCS Istituto Neurologico Carlo Besta

Analisi di II livello disponibili

ENA	◯ Sì ◯ No
ANA	◯ Sì ◯ No
ds DNA	◯ Sì ◯ No
C3, C4	◯ Sì ◯ No
LAC	◯ Sì ◯ No
Ab anti cardiolipina	◯ Sì ◯ No
beta2glicoproteina	◯ Sì ◯ No
es liquor	◯ Sì ◯ No
Markers di neurodegenerazione (tau, p-tau,beta1-40;beta1-42, 14-3-3)	◯ Sì ◯ No
Ab anti cervelletto	◯ Sì ◯ No
Markers neoplastici	◯ Sì ◯ No
Pannello per encefaliti autoimmuni	◯ Sì ◯ No
ab anti R per Ach e anti MUSK	◯ Sì ◯ No
Screening genetico RNF213	◯ Sì ◯ No
Screening genetico Notch3	◯ Sì ◯ No
Screening genetico COl4A1/A2	◯ Sì ◯ No
Screening genetico GLA	◯ Sì ◯ No
Attività dell'enzima alfa-galattosidasi (alfa-GAL)	◯ Sì ◯ No
ANCA	◯ Sì ◯ No
Ricerca mutazione fattore V Leiden	◯ Sì ◯ No
Ricerca mutazione MTHFR	◯ Sì ◯ No
Ricerca mutazione fattore II	◯ Sì ◯ No
Screening genetico HTRA1	◯ Sì ◯ No

Consulenze

Visita neurochirurgica	◯ Sì ◯ No
Visita cardiologica	◯ Sì ◯ No
Visita oftalmologica	◯ Sì ◯ No
Visita dermatologica	◯ Sì ◯ No
Visita reumatologica	◯ Sì ◯ No
Visita ORL	◯ Sì ◯ No
Consulto internistico	◯ Sì ◯ No
Visita epatologica	◯ Sì ◯ No
Visita nefrologica	◯ Sì ◯ No
Valutazione neuropsicologica	◯ Sì ◯ No
Visita psichiatrica	◯ Sì ◯ No
Visita diabetologica	◯ Sì ◯ No
Visita fisiatrica	◯ Sì ◯ No
Visita ematologica	◯ Sì ◯ No
Consulto genetico	◯ Sì ◯ No
Nutrizionista	◯ Sì ◯ No
Neuroradiologo interventista	◯ Sì ◯ No

Procedure

Impianto di loop recorder	◯ Sì ◯ No

Esami strumentali disponibili nel centro

Esecuzione di ecocolordoppler dei vasi epiaortici	◯ Sì ◯ No
Esecuzione di ecocardiogramma transtoracico	◯ Sì ◯ No
Esecuzione di elettrocardiogramma	◯ Sì ◯ No
Esecuzione di elettrocardiogramma Holter	◯ Sì ◯ No
Esecuzione RM cuore	◯ Sì ◯ No

Esecuzione di coronarografia	◯ Sì	◯ No
Esecuzione di ecocardiogramma transesofageo	◯ Sì	◯ No
Esecuzione di TC torace	◯ Sì	◯ No
Esecuzione di angioTC torace	◯ Sì	◯ No
Esecuzione di angioTC torace	◯ Sì	◯ No
Esecuzione di TC addome	◯ Sì	◯ No
Esecuzione di angioTC addome	◯ Sì	◯ No
Esecuzione di TC cervicale (ad es. per politrauma)	◯ Sì	◯ No
Esecuzione di angioTC cervicale (ad es. per politrauma)	◯ Sì	◯ No
Esecuzione di RM encefalo	◯ Sì	◯ No
Esecuzione di angioRM dei vasi cerebrali	◯ Sì	◯ No
Esecuzione di RM perfusione	◯ Sì	◯ No
Tecniche di RM avanzate (ASL, BOLD, etc)	◯ Sì	◯ No
Esecuzione di TC perfusione	◯ Sì	◯ No
Esecuzione di angiografia diagnostica cerebrale	◯ Sì	◯ No
Studio riserva vasomotoria	◯ Sì	◯ No
Esecuzione di doppler transcranico	◯ Sì	◯ No
Esecuzione di doppler tronchi sovraortici	◯ Sì	◯ No
Esecuzione di test vegetativi	◯ Sì	◯ No
Esecuzione di EMG/ENG	◯ Sì	◯ No
Esecuzione di potenziali evocati	◯ Sì	◯ No
Esecuzione di polisonnogramma	◯ Sì	◯ No
Esecuzione di elettroencefalogramma	◯ Sì	◯ No
Esecuzione di gastroscopia	◯ Sì	◯ No
Esecuzione di colonscopia	◯ Sì	◯ No

Esecuzione di biopsia di cute (superficiale e profonda)	◯ Sì ◯ No
Esecuzione di biopsia di muscolo	◯ Sì ◯ No
Esecuzione di eco addome	◯ Sì ◯ No
Esecuzione di SPECT acetazolamide	◯ Sì ◯ No
PET total body	◯ Sì ◯ No
Esecuzione di PET cerebrale con tracciante per amiloide	◯ Sì ◯ No

Percorsi diagnostico terapeutici /PDTA

Moyamoya	◯ Sì ◯ No
CADASIL	◯ Sì ◯ No
Fabry	◯ Sì ◯ No
COL4A1/A2	◯ Sì ◯ No
Sneddon	◯ Sì ◯ No

Moyamoya

Tipo campioni disponibili Sangue intero ______
Plasma-EDTA ______
Plasma-citrato ______
Siero ______
Liquor ______
Cellule (PBMCs) ______
Linee cellulari ______
Tessuti ______
DNA ______
RNA ______

Tempistica raccolta campione Fase acuta ______
Fase cronica ______

Modalità prelievo Sangue intero ______
Plasma-EDTA ______
Plasma-citrato ______
Siero ______
Liquor ______
Cellule (PBMCs) ______
Linee cellulari ______
Tessuti ______
DNA ______
RNA ______

Modalità processamento Sangue intero ______
Plasma-EDTA ______
Plasma-citrato ______
Siero ______
Liquor ______
Cellule (PBMCs) ______
Linee cellulari ______
Tessuti ______

DNA ______
RNA ______

Temperatura di conservazione campione tra prelievo e centrifugazione (per plasma, liquor) Plasma ______
Liquor ______

Condizioni di centrifugazione campione (per plasma, liquor) Plasma ______
Liquor ______

Volume delle aliquote per stoccaggio (in ml) Plasma ______
Liquor ______

Tipo di provette per stoccaggio Plasma ______
Liquor ______
Tessuti ______

Temperatura di stoccaggio Sangue intero ______
Plasma-EDTA ______
Plasma-citrato ______
Siero ______
Liquor ______
Cellule (PBMCs) ______
Linee cellulari ______
Tessuti ______
DNA ______
RNA ______

CADASIL

Tipo campioni disponibili Sangue intero ______
Plasma-EDTA ______
Plasma-citrato ______
Siero ______
Liquor ______
Cellule (PBMCs) ______
Linee cellulari ______
Tessuti ______
DNA ______
RNA ______

Tempistica raccolta campione Fase acuta ______
Fase cronica ______

Modalità prelievo Sangue intero ______
Plasma-EDTA ______
Plasma-citrato ______
Siero ______
Liquor ______
Cellule (PBMCs) ______
Linee cellulari ______
Tessuti ______
DNA ______
RNA ______

Modalità processamento Sangue intero ______
Plasma-EDTA ______
Plasma-citrato ______
Siero ______
Liquor ______
Cellule (PBMCs) ______
Linee cellulari ______
Tessuti ______
DNA ______
RNA ______

Temperatura di conservazione campione tra prelievo e centrifugazione (per plasma, liquor) Plasma ______
Liquor ______

Condizioni di centrifugazione campione (per plasma, liquor) Plasma ______
Liquor ______

Volume delle aliquote per stoccaggio (in ml) Plasma ______
Liquor ______

Tipo di provette per stoccaggio Plasma ______
Liquor ______
Tessuti ______

Temperatura di stoccaggio Sangue intero ______
Plasma-EDTA ______
Plasma-citrato ______
Siero ______
Liquor ______
Cellule (PBMCs) ______
Linee cellulari ______
Tessuti ______
DNA ______
RNA ______

Fabry

Tipo campioni disponibili Sangue intero ______
Plasma-EDTA ______
Plasma-citrato ______
Siero ______
Liquor ______
Cellule (PBMCs) ______
Linee cellulari ______
Tessuti ______
DNA ______
RNA ______

Tempistica raccolta campione Fase acuta ______
Fase cronica ______

Modalità prelievo Sangue intero ______
Plasma-EDTA ______
Plasma-citrato ______
Siero ______
Liquor ______
Cellule (PBMCs) ______
Linee cellulari ______
Tessuti ______
DNA ______
RNA ______

Modalità processamento Sangue intero ______
Plasma-EDTA ______
Plasma-citrato ______
Siero ______
Liquor ______
Cellule (PBMCs) ______
Linee cellulari ______
Tessuti ______
DNA ______
RNA ______

Temperatura di conservazione campione tra prelievo e centrifugazione (per plasma, liquor) Plasma ______
Liquor ______

Condizioni di centrifugazione campione (per plasma, liquor) Plasma ______
Liquor ______

Volume delle aliquote per stoccaggio (in ml) Plasma ______
Liquor ______

Tipo di provette per stoccaggio Plasma ______
Liquor ______
Tessuti ______

Temperatura di stoccaggio Sangue intero ______
Plasma-EDTA ______
Plasma-citrato ______
Siero ______
Liquor ______
Cellule (PBMCs) ______
Linee cellulari ______
Tessuti ______
DNA ______
RNA ______

COL4A1/A2

Tipo campioni disponibili Sangue intero ______
Plasma-EDTA ______
Plasma-citrato ______
Siero ______
Liquor ______
Cellule (PBMCs) ______
Linee cellulari ______
Tessuti ______
DNA ______
RNA ______

Tempistica raccolta campione Fase acuta ______
Fase cronica ______

Modalità prelievo Sangue intero ______
Plasma-EDTA ______
Plasma-citrato ______
Siero ______
Liquor ______
Cellule (PBMCs) ______
Linee cellulari ______
Tessuti ______
DNA ______
RNA ______

Modalità processamento Sangue intero ______
Plasma-EDTA ______
Plasma-citrato ______
Siero ______
Liquor ______
Cellule (PBMCs) ______
Linee cellulari ______
Tessuti ______
DNA ______
RNA ______

Temperatura di conservazione campione tra prelievo e centrifugazione (per plasma, liquor) Plasma ______
Liquor ______

Condizioni di centrifugazione campione (per plasma, liquor) Plasma ______
Liquor ______

Volume delle aliquote per stoccaggio (in ml) Plasma ______
Liquor ______

Tipo di provette per stoccaggio Plasma ______
Liquor ______
Tessuti ______

Temperatura di stoccaggio Sangue intero ______
Plasma-EDTA ______
Plasma-citrato ______
Siero ______
Liquor ______
Cellule (PBMCs) ______
Linee cellulari ______
Tessuti ______
DNA ______
RNA ______

Sneddon

Tipo campioni disponibili Sangue intero ______
Plasma-EDTA ______
Plasma-citrato ______
Siero ______
Liquor ______
Cellule (PBMCs) ______
Linee cellulari ______
Tessuti ______
DNA ______
RNA ______

Tempistica raccolta campione Fase acuta ______
Fase cronica ______

Modalità prelievo Sangue intero ______
Plasma-EDTA ______
Plasma-citrato ______
Siero ______
Liquor ______
Cellule (PBMCs) ______
Linee cellulari ______
Tessuti ______
DNA ______
RNA ______

Modalità processamento Sangue intero ______
Plasma-EDTA ______
Plasma-citrato ______
Siero ______
Liquor ______
Cellule (PBMCs) ______
Linee cellulari ______
Tessuti ______
DNA ______
RNA ______

Temperatura di conservazione campione tra prelievo e centrifugazione (per plasma, liquor) Plasma ______
Liquor ______

Condizioni di centrifugazione campione (per plasma, liquor) Plasma ______
Liquor ______

Volume delle aliquote per stoccaggio (in ml) Plasma ______
Liquor ______

Tipo di provette per stoccaggio Plasma ______
Liquor ______
Tessuti ______

Temperatura di stoccaggio Sangue intero ______
Plasma-EDTA ______
Plasma-citrato ______
Siero ______
Liquor ______
Cellule (PBMCs) ______
Linee cellulari ______
Tessuti ______
DNA ______
RNA ______

Susac

Tipo campioni disponibili Sangue intero ______
Plasma-EDTA ______
Plasma-citrato ______
Siero ______
Liquor ______
Cellule (PBMCs) ______
Linee cellulari ______
Tessuti ______
DNA ______
RNA ______

Tempistica raccolta campione Fase acuta ______
Fase cronica ______

Modalità prelievo Sangue intero ______
Plasma-EDTA ______
Plasma-citrato ______
Siero ______
Liquor ______
Cellule (PBMCs) ______
Linee cellulari ______
Tessuti ______
DNA ______
RNA ______

Modalità processamento Sangue intero ______
Plasma-EDTA ______
Plasma-citrato ______
Siero ______
Liquor ______
Cellule (PBMCs) ______
Linee cellulari ______
Tessuti ______
DNA ______
RNA ______

Temperatura di conservazione campione tra prelievo e centrifugazione (per plasma, liquor) Plasma ______
Liquor ______

Condizioni di centrifugazione campione (per plasma, liquor) Plasma ______
Liquor ______

Volume delle aliquote per stoccaggio (in ml) Plasma ______
Liquor ______

Tipo di provette per stoccaggio Plasma ______
Liquor ______
Tessuti ______

Temperatura di stoccaggio Sangue intero ______
Plasma-EDTA ______
Plasma-citrato ______
Siero ______
Liquor ______
Cellule (PBMCs) ______
Linee cellulari ______
Tessuti ______
DNA ______
RNA ______

Tecnologie analitiche

ELISA convenzionale	◯ Sì ◯ No
LUMIPULSE (Fujirebio)	◯ Sì ◯ No
Elecsys (Roche)	◯ Sì ◯ No
Luminex Xmap (Luminex)	◯ Sì ◯ No
SiMoA (Quanterix)	◯ Sì ◯ No
Mesoscale Discovery (MSD)	◯ Sì ◯ No
ELLA (Bio-techne)	◯ Sì ◯ No
Single Molecule Counting (SMC, Merck)	◯ Sì ◯ No
Pro-quantum Immunoassay (ThermoFisher)	◯ Sì ◯ No
Olink-PEA (Olink proteomics)	◯ Sì ◯ No

Altre metodiche multiparametriche, ultrasensibili

____

Database disponibili (formato)

Excel	◯ Sì ◯ No
Redcap	◯ Sì ◯ No
Access	◯ Sì ◯ No
Altro	◯ Sì ◯ No
Specificare database	__________________________
