# Supplementary material for: ALIGNED Network for rare cerebrovascular diseases: methodology and preliminary results
Source: Neurol Sci. 2026 Jun 22;47(7):584. doi: 10.1007/s10072-026-09183-1 (PMC13287270; doi:10.1007/s10072-026-09183-1)
Supplement: Supplementary file 7 — Supplementary file7 (PDF 290 KB) [file 10072_2026_9183_MOESM7_ESM.pdf]

Supplementary file 7:

List of the second-tier analyses currently available in the 31 centers from the Northern regions of Italy and in 13 centers located in the Southern ones.

	CENTERS FROM CENTRAL-NORTHERN REGIONS OF ITALY. N=31		CENTERS FROM SOUTHERN REGIONS OF ITALY. N=13		
**Available 2nd tier analyses**	**Yes (n/N; %)**	**No (n/N; %)**	**Yes (n/N; %)**	**No (n/N; %)**	**p-value**
**ENA**	31; (100)	0; (0)	13; (100)	0; (0)	-
**ANA**	31; (100)	0; (0)	12; (92.3)	1; (7.7)	-
**ds DNA**	30; (96.8)	1; (3.2)	12; (92.3)	1; (7.7)	0 .516375
**C3. C4**	31; (100)	0; (0)	13; (100)	0; (0)	-
**ANCA**	31; (100)	0; (0)	0; (0)	13; (100)	-
**LAC**	31; (100)	0; (0)	12; (92.3)	1; (7.7)	-
**Anti-cardiolipin Antibodies**	31; (100)	0; (0)	13; (100)	0; (0)	-
**Anti- β2 glicoprotein antibodies [a]**	30; (96.8)	0; (0)	12; (92.3)	1; (7.7)	-
**Anti-cerebellar antibodies**	21; (67.7)	10; (32.3)	7; (53.8)	6; (46.2)	0.381996
**Panel for autoimmune encephalitis**	24; (77.4)	7; (22.6)	7; (53.8)	6; (46.2)	0.117895
**Anti-AchR and anti-MUSK antibodies**	20; (64.5)	11; (35.5)	8; (61.5)	5; (38.5)	0.851399
**Neurodegeneration markers***	23; (74.2)	8; (25.8)	5; (38.5)	8; (61.5)	**0.024576**
**Neoplastic markers**	30; (96.8)	1; (3.2)	13; (100)	0; (0)	-
**CSF examination**	31; (100)	0; (0)	13; (100)	0; (0)	-
**RNF213 mutation [b]**	8; (25.8)	22; (71)	3; (23.1)	10; (76.9)	0.804308

**Notch3 mutation**	19; (61.3)	12; (38.7)	10; (76.9)	3; (23.1)	0.318236
**COl4A1 mutation**	15; (48.4)	16; (51.6)	9; (69.2)	4; (30.8)	0.205201
**GLA mutation [c]**	17; (54.8)	14; (45.2)	4; (30.8)	8; (61.5)	0.205724
**HTRA1 mutation [d]**	13; (41.9)	18; (58.1)	3; (23.1)	9; (69.2)	0.30276.

*****tau. p-tau. beta1-40; beta1-42. 14-3-3. One value is missing. and its status (available or unavailable) is unknown for centres from both Central-Northern regions ([a. b]) and Southern regions ([c. d]). Abbreviations: Antinuclear antibodies (ANA); anti-neutrophil cytoplasmic antibodies (ANCA); cerebrospinal fluid (CSF); extractable nuclear antigen (ENA); lupus anti-coagulant (LAC). Statistical significance - has been estimated through Chi Square test. when applicable. P value <0.05 is considered statistically relevant.
