# Supplementary material for: ALIGNED Network for rare cerebrovascular diseases: methodology and preliminary results
Source: Neurol Sci. 2026 Jun 22;47(7):584. doi: 10.1007/s10072-026-09183-1 (PMC13287270; doi:10.1007/s10072-026-09183-1)
Supplement: Supplementary file 8 — Supplementary file8 (PDF 193 KB) [file 10072_2026_9183_MOESM8_ESM.pdf]

Supplementary file 8: List of the main multidisciplinary consultancies currently available in the 31 centers from the Northern regions of Italy and in the 13 centers located in the Southern ones.

	**CENTERS FROM NORTHERN REGIONS OF ITALY. N=31**		**CENTERS FROM SOUTHERN REGIONS OF ITALY. N=13**		
**Consultancy**	**Yes (n/N; %)**	**No (n/N; %)**	**Yes (n/N; %)**	**No (n/N; %)**	**p-value**
**Neurosurgical consultation**	28; (90.3)	3; (9.7)	11; (84.6)	2; (15.4)	0.586282
**Cardiological consultation**	31; (100)	0; (0)	13; (100)	0; (0)	-
**Ophthalmological consultation**	31; (100)	0; (0)	11; (85.6)	2; (15.4)	-
**Dermatological consultation**	30; (96.8)	1; (3.2)	12; (92.3)	1; (7.7)	0.516375
**Rheumatology consultation**	31; (100)	0; (0)	8; (61.5)	5; (38.5)	-
**Ear-nose-throat consultation**	31; (100)	0; (0)	12; (92.3)	1; (7.7)	-
**Internal Medicine consultation**	31; (100)	0; (0)	13; (100)	0; (0)	-
**Hepatology consultation**	30; (96.8)	1; (3.2)	10; (76.9)	3; (23.1)	**0.036636**
**Nephrology consultation**	31; (100)	0; (0)	12; (92.3)	1; (7.7)	-
**Neuropsychological evaluation**	28; (90.3)	3; (9.7)	10; (76.9)	3; (23.1)	0.237332
**Psychiatric consultation**	31; (100)	0; (0)	10; (76.9)	3; (23.1)	-
**Diabetological examination**	31; (100)	0; (0)	13; (100)	0; (0)	-
**Physiatric consultation**	31; (100)	0; (0)	12; (92.3)	1; (7.7)	-
**Haematological consultation**	31; (100)	0; (0)	11; (85.6)	2; (15.4)	-
**Genetic consultation**	25; (80.6)	6; (19.4)	8; (61.5)	5; (38.5)	0.181746

**Nutritionist consultation**	30; (96.8)	1; (3.2)	7; (53.8)	6; (46.2)	**0.000382**

Chi Square test has been used to assess statistical significance. when applicable. P value <0.05 is considered statistically relevant.
