# Supplementary material for: ALIGNED Network for rare cerebrovascular diseases: methodology and preliminary results
Source: Neurol Sci. 2026 Jun 22;47(7):584. doi: 10.1007/s10072-026-09183-1 (PMC13287270; doi:10.1007/s10072-026-09183-1)
Supplement: Supplementary file 9 — Supplementary file9 (PDF 182 KB) [file 10072_2026_9183_MOESM9_ESM.pdf]

Supplementary file 9: Availability of rCVD diagnostic and treatment protocols assessed in the 31 centers from the Northern regions of Italy and in the 13 centers located in the Southern ones.

	CENTERS FROM NORTHERN REGIONS OF ITALY, N=31		CENTERS FROM SOUTHERN REGIONS OF ITALY, N=13		
**Availabilty of diagnostic and treatment protocols**	**Yes (n/N; %)**	**No (n/N; %)**	**Yes (n/N; %)**	**No (n/N; %)**	**p-value**
**CADASIL**	6; (19,4)	25; (80,6)	2; (15,4)	11; (84,6)	0,755399
**COL4A1/A2**	3; (9,7)	28; (90,3)	1; (7,7)	12; (92,3)	0,834464
**Fabry**	10; (32,3)	21; (67,7)	2; (15,4)	11; (84,6)	0,251541
**Sneddon**	3; (9,7)	28; (90,3)	1; (7,7)	12; (92,3)	0,834464
**Moyamoya**	3; (9,7)	28; (90,3)	1; (7,7)	12; (92,3)	0,834464
