# Supplementary material for: ALIGNED Network for rare cerebrovascular diseases: methodology and preliminary results
Source: Neurol Sci. 2026 Jun 22;47(7):584. doi: 10.1007/s10072-026-09183-1 (PMC13287270; doi:10.1007/s10072-026-09183-1)
Supplement: Supplementary file 10 — Supplementary file10 (PDF 292 KB) [file 10072_2026_9183_MOESM10_ESM.pdf]

ALIGNED project

Andrea Zini[1], Vincenzo Di Lazzaro[2], Michelangelo Mancuso[3], Alberto Chiti[4], Maria Guarino[5], Domenico Di Noia[6], Marialuisa Zedde[7], Letizia Maria Cupini[8], Giovanni Frisullo[9], Giovanni Merlino[10], Vincenzo Andreone[11], Antonio Ciacciarelli[12], Marcella Caggiula[13], Marina Mannino[14], Giuseppe Rinaldi[15], Elena Minguzzi[16], Giulia Fiume[17], Maurizio Paciaroni[18], Maria Stella Aniello[19], Maria Luisa Dell'Acqua[20], Antonio Toscano[21], Dario Alimonti[22], Simona Marcheselli[23], Federico Mazzacane[24], Alessandro Padovani[25], Luigi Caputi[26], Del Sette Massimo[27], Luisa Chiapparini[28], Marina Diomedi[29], Marco Longoni[30], Michele Romoli[30], Cinzia Finocchi[31], Massimiliano Godani[32], Sara Bonato[33], Susanna Diamanti[34] Francesca Gragnani[35], Roberto De Simone[36], Maria Vittoria De Angelis[37, 38], Leonardo Pantoni[39], Carla Zanferrari[40], Luigi Maria Edoardo Grimaldi[41], Alessia Giossi[42], Gaspare Scaglione[43], Cristina Motto[44], Maria Valeria Saddi[45], Patrizia Nencini[46], Francesca Pescini[46], Laura Fusi[47], Maria Grazia Riggio[48]

[1] IRCCS Istituto delle Scienze Neurologiche di Bologna Maggiore Hospital Bologna Italy
[2] Università Campus Bio-Medico di Roma: Roma, Lazio, Italy
[3] Neurological Clinic, AOUP & University of Pisa, Italy
[4] Neurology Unit Apuane Hospital Massa Italy, Italy
[5] IRCCS Istituto Delle Scienze Neurologiche di Bologna: Bologna, Emilia-Romagna, Italy
[6] Institute of Cardiovascular Diseases, University of Bari, Bari, Italy
[7] Neurology Unit, Stroke Unit, Istituto di Ricovero e Cura a Carattere Scientifico, Arcispedale Santa Maria Nuova, Reggio Emilia, Italy
[8] Stroke Unit S. Eugenio Hospital Rome, Roma, Italy
[9] Neuroscienze, Organi di Senso e Torace Fondazione Policlinico Universitario Agostino Gemelli Rome, Roma, Italy
[10] Department of Neurosciences, Santa Maria della Misericordia University Hospital, Udine, Italy
[11] Stroke Unit AORN Antonio Cardarelli Naples Italy
[12] Policlinico Umberto I: Rome, Lazio, Italy
[13] Department of Neurology Vito Fazi Hospital Lecce Italy
[14] Department of Neurology AOOR Villa Sofia-Cervello Palermo Italy
[15] Department of Neurology Di Venere Hospital Bari, Italy
[16] Ospedale "Santa Maria delle Croci" di Ravenna: Ravenna, Emilia-Romagna, Italy
[17] Centro Neurolesi Bonino Pulejo: Messina, Italy
[18] Università degli Studi di Ferrara: Ferrara, Emilia-Romagna, Italy
[19] Department of Neurological and Psychiatric Sciences, University of Bari, Italy
[20] Azienda Ospedaliero-Universitaria di Modena: Modena, Emilia-Romagna, Italy
[21] Department of Clinical and Experimental Medicine, Reference Center for Rare Neuromuscular Disorders, University of Messina, Messina, Italy
[22] Aziende Socio Sanitarie Territoriale Papa Giovanni XXIII: Bergamo, Lombardia, Italy
[23] IRCCS Humanitas Research Hospital: Rozzano, Italy
[24] Department of Emergency Neurology and Stroke Unit, IRCCS Mondino Foundation, Pavia, Italy
[25] Dipartimento di Scienze Cliniche e Sperimentali, Clinica Neurologica, Università degli Studi di Brescia, P le Spedali Civili 1, Brescia, Italy
[26] Department of Cardiocerebrovascular diseases ASST Ospedale Maggiore di Crema Crema, Italy
[27] Struttura Complessa di Neurologia, Ente Ospedaliero Ospedali Galliera, Genoa, Italy
[28] Radiodiagnostic Department, Fondazione IRCCS Policlinico San Matteo, Pavia, Italy
[29] Stroke Unit, Department of Systems Medicine, University of Tor Vergata, Rome, Italy
[30] Neurology and Stroke Unit, Department of Neuroscience, Bufalini Hospital, Cesena, Italy

[31] Department of Neurosciences and Neurorehabilitation, University of Genova, Italy
[32] Neurological Department, S. Andrea Hospital, La Spezia, Italy
[33] Fondazione IRCCS Ca' Granda Ospedale Maggiore Policlinico, Stroke Unit, Milan, Italy
[34] Stroke Unit, Department of Medical and Surgical Sciences, San Gerardo Hospital, Milan Center for Neuroscience, University of Milano-Bicocca, Milano, Italy
[35] Sandro Pertini Hospital, Neurology, Rome, Italy
[36] Neurology Unit, Hospital F. Spaziani, Frosinone, Italy
[37] Stroke Unit "S.Spirito" Hospital Pescara, Italy
[38] Department of Neurology SS Annunziata Hospital Chieti, Italy
[39] Neurology, L. Sacco Department of Biomedical and Clinical Sciences, University of Milan, Italy
[40] ASST Santi Paolo e Carlo, Milano, Italy
[41] Fondazione Istituto G. Giglio di Cefalù, Italy
[42] U.O.Neurologia, Istituti Ospitalieri, ASST Cremona, Cremona, Italy
[43] Department of Neurology General Regional Hospital "F. Miulli" Acquaviva delle Fonti Italy
[44] Stroke Unit, Department of Neurological Sciences, Azienda Ospedaliera Niguarda Ca Granda, Milan, Italy
[45] Neurology Department, San Francesco Hospital, Nuoro, Italy
[46] Department of Neurological and Psychiatric Sciences, University of Florence, Italy
[47] Neurology Department of ASST Lariana, Ospedale Sant'Anna, San Fermo della Battaglia, Italy
[48] Neurology Unit, ASST Rhodense, V.le Forlanini, 95, Garbagnate Milanese, Italy
